# Chitosan modulates *Pochonia chlamydosporia* gene expression during nematode egg parasitism

**DOI:** 10.1111/1462-2920.15408

**Published:** 2021-02-05

**Authors:** Marta Suarez‐Fernandez, Christine Sambles, Federico Lopez‐Moya, María J. Nueda, David J. Studholme, Luis Vicente Lopez‐Llorca

**Affiliations:** ^1^ Laboratory of Plant Pathology, Department of Marine Sciences and Applied Biology University of Alicante Alicante 03080 Spain; ^2^ Multidisciplinary Institute for Environmental Studies (MIES) Ramon Margalef, University of Alicante Alicante 03080 Spain; ^3^ School of Biosciences, University of Exeter Exeter UK; ^4^ Mathematics Department University of Alicante Alicante 03080 Spain

## Abstract

Climate change makes plant‐parasitic nematodes (PPN) an increasing threat to commercial crops. PPN can be managed sustainably by the biocontrol fungus *Pochonia chlamydosporia* (Pc). Chitosan generated from chitin deacetylation enhances PPN parasitism by Pc. In this work, we investigate the molecular mechanisms of Pc for chitosan resistance and root‐knot nematode (RKN) parasitism, using transcriptomics. Chitosan and RKN modify the expression of Pc genes, mainly those involved in oxidation–reduction processes. Both agents significantly modify the expression of genes associated to 113 GO terms and 180 Pc genes. Genes encoding putative glycoproteins (Pc adhesives) to nematode eggshell, as well as genes involved in redox, carbohydrate and lipid metabolism trigger the response to chitosan. We identify genes expressed in both the parasitic and endophytic phases of the Pc lifecycle; these include proteases, chitosanases and transcription factors. Using the Pathogen—Host Interaction database (PHI‐base), our previous RNA‐seq data and RT‐PCR of Pc colonizing banana we have investigated genes expressed both in the parasitic and endophytic phases of Pc lifecycle.

## Introduction

Root‐knot nematodes (RKN) are a persistent problem in fruit and vegetable crops (Ralmi and Khandaker, 2016). Biological control is used to reduce and avoid the use of toxic chemical nematicides and fumigants, introducing non‐harmful organisms for plants that can manage pests and diseases in a sustainable way (Mankau, 1980).

*Pochonia chlamydosporia* (=*Metacordyceps chlamydosporia*) (Goddard) Zare and Gams (Pc) is a nematophagous fungus used for biocontrol of RKN (*Meloidogyne* spp.) (Forghani and Hajihassani, 2020), cyst nematodes (*Heterodera* spp. and *Globodera* spp.) (Willcox and Tribe, 1974; Manzanilla‐Lopez et al., 2011) and false RKN (*Nacobbus* spp.) (Flores‐Camacho *et al*., 2008). Pc is distributed worldwide and may also adopt saprotrophic and endophytic lifestyles (Bordallo *et al*., 2002; Maciá‐Vicente *et al*., 2009; Manzanilla‐López *et al*., 2011; Zavala‐Gonzalez *et al*., 2017).

Chitosan is a linear polymer of β‐(1‐4)‐linked *N*‐acetyl‐2‐amino‐2‐deoxy‐d‐glucose (acetylated) and 2‐amino‐2‐deoxy‐d‐glucose (deacetylated) (Kaur and Dhillon, 2014). This polymer is an elicitor of plant defences (Benhamou and Thériault, 1992; Lafontaine and Benhamou, 1996; Yin *et al*., 2016; Suarez‐Fernandez *et al*., 2020) and has antifungal activity (Shih *et al*., 2019), inhibiting or killing fungal pathogens. Chitosan also promotes the growth of resistant fungi such as Pc and entomopathogenic fungi (Palma‐Guerrero *et al*., 2007). The molecular mechanisms that determine whether a fungus is resistant or sensitive to chitosan remain to be determined. Pc is resistant to chitosan and can use it as a nutrient source (Palma‐Guerrero *et al*., 2010). Chitosan‐resistant fungi produce valuable bioproducts from chitosan degradation due to their chitinases and chitosanases (Kaczmarek *et al*., 2019). The Pc genome encodes a high number of chitosanases that are induced during nematode egg parasitism (Aranda‐Martinez *et al*., 2016). Chitosan improves efficiency in reducing nematode pests by nematophagous fungi (Escudero *et al*., 2017; Mwaheb *et al*., 2017). Therefore, combining Pc and chitosan could be a good strategy to manage PPN infections in plants.

Global unbiased transcriptomic analyses are a useful tool for determining genes involved in the response of fungi to elicitors such as chitosan (Zhang *et al*., 2020). These analyses also show which genes are involved in biological processes, such as pathogenicity to nematodes (Balestrini *et al*., 2019). The activation of specific genes can trigger the transition from endophytism to parasitism and vice versa in fungi (Fesel and Zuccaro, 2016; Zhang *et al*., 2018). In view of the multitrophic interaction of chitosan and Pc with plants and PPN, it is relevant to study genes in common between endophytism and pathogenicity. This could reveal which genes are key for interacting with other organisms.

This work aims to analyse the molecular mechanisms that are activated in Pc when interacting with chitosan, RKN or both as well as to unravel which mechanisms would increase RKN parasitism by Pc. These mechanisms could explain what makes a fungus resistant or sensitive to chitosan. In this work we focus on identifying which genes are shared in both lifestyles: RKN parasitism and plant root endophytism.

## Results

### Chitosan and RKN eggs modify Pc gene expression

**Fig 1:**
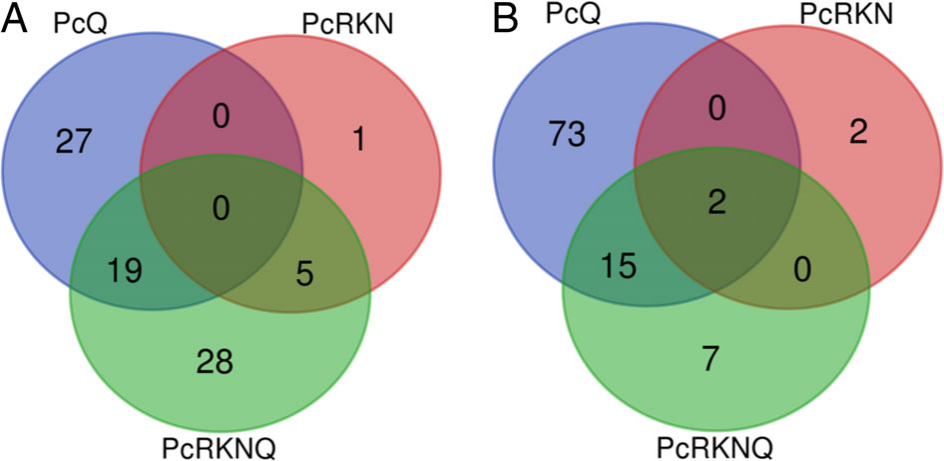
Chitosan and RKN modify *P*. *chlamydosporia* gene expression. Venn diagrams show a total of 80 upregulated (A) and 99 downregulated (B) genes when a threshold of ±2 in log_2_ fold change is set and adjusted *P*‐value is taken into account (44.7% vs. 55.3%). Treatments: PcQ, *P*. *chlamydosporia* with chitosan (1 mg·ml^−1^); PcRKN, *P*. *chlamydosporia* with *M*. *javanica* eggs (1 egg·μl^−1^); PcRKNQ, *P*. *chlamydosporia* with *M*. *javanica* eggs (1 egg·μl^−1^) and chitosan (1 mg ml^−1^). All treatments were applied for 4 days.

Twelve samples were processed (four treatments per triplicate), with average total read bases (total reads × read length) of 17 894 300 929 yielding approximately 100 GB of transcriptomic raw data. When using DESeq2 in a genome‐guided analysis to Pc123 genome 39 009 transcripts in 20 502 loci were found. In addition, 5746, 710 and 6595 genes were expressed in Pc treated with chitosan (PcQ), Pc treated with nematodes (PcRKN) and Pc treated with chitosan and nematode eggs (PcRKNQ) respectively (all data are available in Supplementary Information File). Considering all treatments together, 80 upregulated and 99 downregulated genes can be found with a threshold of ±2 in log_2_ fold change values (Fig. 1; Tables 1, 2, 3). In PcQ there are 136 differentially expressed genes (DEG, up and downregulated), in PcRKNQ 75 DEG, while in PcRKN only 10. Therefore, chitosan is a stronger modulator of Pc gene expression than RKN eggs are. Two genes encoding proteins (RZR63781.1, ribonuclease H‐like protein and RZR63940.1, ankyrin repeat protein) are downregulated in all treatments. These results are supported by qRT‐PCR (Supplementary Fig. 1).

**Table 1:** Differentially expressed genes in PcQ treatment.

GenBank accession	Sequence description	Log_2_ fold change	Adj. *P*‐value
RZR66026.1	Secreted aspartic proteinase precursor	8.155	6.36E‐13
RZR63431.1	Peptidase S8/S53, subtilisin/kexin/sedolisin	8.029	5.38E‐12
RZR59126.1	Hypothetical protein I1G_00009219	7.792	3.35E‐04
RZR63795.1	Glycoside hydrolase family 75	6.745	3.92E‐07
RZR63158.1	Peptidase A1	6.520	3.68E‐11
RZR61856.1	Glucokinase	6.489	1.27E‐09
RZR62208.1	Peptidase A4 family protein	6.016	3.61E‐11
RZR61845.1	Glycoside hydrolase family 75 protein	5.858	5.70E‐05
RZR66080.1	CipC1 protein, concanamycin induced protein C	5.738	NA
RZR62018.1	Metallo‐endopeptidase	5.064	4.21E‐05
RZR61625.1	Cytochrome P450 ClCP1	5.053	7.12E‐06
RZR61472.1	Oligopeptide transporter OPT‐like protein	4.947	6.16E‐04
RZR70313.1	Chitosanase CSN1	4.664	2.15E‐06
RZR61846.1	Major facilitator superfamily domain, general substrate transporter	4.641	8.25E‐08
RZR65223.1	WSC domain‐containing protein	4.559	1.09E‐04
RZR68359.1	Nucleotide‐binding, alpha‐beta plait	4.427	5.32E‐07
RZR60329.1	Carboxyl‐terminal proteinase	3.907	5.36E‐04
RZR61987.1	Tripeptidyl‐peptidase 1 precursor	3.851	6.55E‐06
RZR62940.1	Glycoside hydrolase family 75	3.801	9.84E‐04
RZR66604.1	Hypothetical protein VFPPC_00169	3.368	2.91E‐03
RZR68451.1	Maltose permease	3.141	5.70E‐05
RZR62148.1	P‐loop containing nucleoside triphosphate hydrolase protein	3.132	4.50E‐03
RZR69895.1	Putative polyketide synthase	2.968	4.94E‐03
RZR70338.1	Glycoside hydrolase, subgroup, catalytic core	2.916	4.59E‐03
RZR66611.1	Hypothetical protein I1G_00003511	2.630	1.15E‐02
RZR66588.1	AtmA protein	2.622	2.81E‐07
RZR70289.1	Extracellular soluble lytic transglycosylase	2.599	7.85E‐06
RZR60800.1	Hypothetical protein VFPPC_03295	2.575	2.39E‐03
RZR64639.1	Hypothetical protein I1G_00010772	2.561	2.28E‐04
RZR59556.1	Solid‐state culture specific ATP‐grasp domain protein	2.530	4.53E‐03
RZR68085.1	Sugar transporter family protein	2.526	3.84E‐03
RZR64446.1	Cytochrome P450 6A1	2.469	1.28E‐03
RZR67153.1	Hypothetical protein I1G_00011003	2.454	1.85E‐02
RZR65901.1	l‐amino‐acid oxidase	2.447	1.39E‐02
RZR64948.1	Fungal chitosanase	2.411	4.53E‐03
RZR69865.1	Cytochrome P450 oxidoreductase	2.383	2.99E‐03
RZR63081.1	Glycoside hydrolase family 2 protein	2.379	4.99E‐03
RZR63277.1	l‐ascorbate oxidase	2.333	9.16E‐03
RZR63926.1	Lactonase, 7‐bladed beta‐propeller domain‐containing protein	2.280	1.76E‐02
RZR63275.1	Cytochrome b561, eukaryote	2.169	2.16E‐03
RZR69240.1	APSES transcription factor	2.140	7.08E‐03
RZR64143.1	Flavin‐binding monooxygenase‐like family protein	2.104	2.06E‐02
RZR59143.1	Hypothetical protein I1G_00011200	2.094	1.37E‐02
RZR63080.1	Nuclear distribution protein pac‐1a	2.049	1.31E‐02
RZR69181.1	Hypothetical protein I1G_00010741	2.034	1.36E‐03
RZR67141.1	Killer toxin, Kp4/SMK‐like, core	2.008	1.02E‐02
RZR65937.1	Nitrate reductase (NADH)	−2.024	2.37E‐02
RZR60029.1	Aldo/keto reductase	−2.034	8.26E‐03
RZR63940.1	Ankyrin repeat protein	−2.040	1.36E‐03
RZR67896.1	MFS transporter, SP family, general alpha glucoside:H+ symporter	−2.051	4.65E‐03
RZR66454.1	Hypothetical protein I1G_00004264	−2.055	9.55E‐03
RZR65015.1	Nitrate reductase‐like protein	−2.056	4.99E‐03
RZR69659.1	Integral membrane protein	−2.057	5.70E‐05
RZR66259.1	Cytochrome P450	−2.068	2.61E‐03
RZR62313.1	Hypothetical protein I1G_00005303	−2.097	1.24E‐02
RZR67230.1	NADP‐dependent alcohol dehydrogenase C	−2.103	7.72E‐03
RZR64929.1	FMN‐dependent alpha‐hydroxy acid dehydrogenase	−2.104	1.08E‐02
RZR59541.1	Hypothetical protein I1G_00010383	−2.107	2.21E‐02
RZR66613.1	Oxidoreductase	−2.114	7.04E‐03
RZR67320.1	Related to double substrate‐specificity short chain dehydrogenase/reductase 2	−2.149	2.72E‐02
RZR65483.1	MFS transporter	−2.157	1.89E‐02
RZR62047.1	Cell surface flocculin, putative	−2.178	1.24E‐02
RZR61511.1	Reductase	−2.181	2.16E‐03
RZR59404.1	Transcription factor	−2.183	1.03E‐02
RZR66789.1	Predicted protein	−2.185	2.18E‐02
RZR66794.1	Hypothetical protein I1G_00009684	−2.193	2.55E‐02
RZR67810.1	l‐isoaspartate O‐methyltransferase	−2.220	9.55E‐03
RZR61034.1	Thioredoxin domain‐containing protein	−2.232	2.91E‐03
RZR64973.1	NA	−2.234	0.01
RZR70090.1	Zinc transporter protein	−2.240	2.35E‐02
RZR66702.1	QI74 protein	−2.258	2.49E‐02
RZR70243.1	MUS38‐like protein	−2.274	4.08E‐04
RZR59616.1	Oxidoreductase	−2.275	1.15E‐02
RZR62422.1	Related to short‐chain alcohol dehydrogenase	−2.277	4.10E‐03
RZR66328.1	S‐(hydroxymethyl)glutathione dehydrogenase	−2.283	9.35E‐03
RZR70238.1	Predicted protein	−2.283	1.36E‐03
RZR62423.1	Catalase A	−2.301	9.61E‐03
RZR64023.1	Hypothetical protein I1G_00011265	−2.304	1.35E‐02
RZR64512.1	MIP transporter	−2.324	1.32E‐02
RZR67229.1	3‐dehydroshikimate dehydratase protein	−2.328	6.62E‐03
RZR67902.1	Alpha/beta hydrolase domain‐containing protein	−2.340	1.87E‐02
RZR59365.1	SUR7 protein	−2.359	6.50E‐03
RZR65734.1	Related to diacylglycerol pyrophosphate phosphatase DPP1	−2.380	2.56E‐03
RZR70207.1	Major facilitator superfamily domain, general substrate transporter	−2.386	NA
RZR67256.1	Oligosaccharide translocation protein RFT1	−2.412	6.87E‐03
RZR60041.1	Ribonuclease H‐like protein	−2.448	1.88E‐02
RZR67257.1	Putative phosphatidylinositol phosphate kinase	−2.470	3.98E‐04
RZR69040.1	Double‐stranded RNA binding motif domain‐containing protein	−2.471	2.39E‐03
RZR68713.1	BTB domain transcription factor	−2.478	1.69E‐03
RZR65017.1	Short‐chain dehydrogenase/reductase family protein	−2.486	5.58E‐03
RZR66003.1	Potassium channel	−2.496	1.40E‐03
RZR65013.1	3‐oxoacyl‐(acyl‐carrier‐protein) reductase	−2.522	1.04E‐02
RZR67228.1	6‐phosphogluconate dehydrogenase, decarboxylating	−2.539	5.23E‐03
RZR65468.1	Hypothetical protein I1G_00002210	−2.550	2.91E‐03
RZR67249.1	Hypothetical protein I1G_00007967	−2.594	1.34E‐04
RZR65014.1	Lactamase_B domain‐containing protein	−2.619	1.40E‐02
RZR65332.1	Hypothetical protein I1G_00001300	−2.625	1.65E‐04
RZR62987.1	C6 transcription factor	−2.667	1.20E‐02
RZR66334.1	Protein kinase domain protein	−2.711	6.50E‐03
RZR64692.1	Hypothetical protein I1G_00007179	−2.717	3.78E‐03
RZR64363.1	ATP synthase protein 9 (Lipid‐binding protein)	−2.745	4.58E‐03
RZR67237.1	Choline and nitrogen mustard permease	−2.795	5.61E‐04
RZR61033.1	Glucose repressible protein Grg1	−2.810	8.46E‐07
RZR69039.1	DUF1929 multi‐domain protein	−2.824	1.18E‐03
RZR69535.1	NAD(P)‐binding domain protein	−2.844	8.91E‐04
RZR66687.1	l‐amino acid oxidase	−2.848	1.64E‐02
RZR64977.1	Related to molybdopterin biosynthesis protein moeA	−2.849	4.99E‐03
RZR69169.1	Transcriptional regulatory protein GAL4	−2.866	3.21E‐03
RZR67231.1	d‐xylulose 5‐phosphate/d‐fructose 6‐phosphate phosphoketolase	−2.874	2.56E‐03
RZR66951.1	Hypothetical protein I1G_00006786	−2.876	1.26E‐02
RZR60802.1	Siderophore iron transporter mirB	−3.029	1.50E‐02
RZR60798.1	Alcohol acetyltransferase	−3.046	1.50E‐02
RZR63453.1	Protein bli‐3	−3.067	1.92E‐03
RZR66605.1	Beta‐lactamase‐like protein	−3.069	5.20E‐04
RZR62135.1	MFS transporter	−3.134	5.81E‐04
RZR68164.1	MARVEL‐like domain protein	−3.165	5.35E‐04
RZR61718.1	Ctr copper transporter	−3.188	1.39E‐02
RZR59602.1	30 kDa heat shock protein	−3.333	1.42E‐04
RZR60799.1	Transferase family protein	−3.417	1.21E‐02
RZR67450.1	Histone acetylase complex subunit	−3.523	3.28E‐03
RZR65938.1	HHE domain containing protein	−3.575	1.31E‐03
RZR62210.1	Major allergen Asp f 2‐like protein	−4.322	3.45E‐05
RZR62374.1	Ctr copper transporter family protein	−4.413	7.43E‐03
RZR63781.1	Ribonuclease H‐like protein	−4.450	8.42E‐04
RZR63947.1	Cycloheximide resistance protein	−4.655	3.53E‐05
RZR68445.1	FAD binding domain protein	−5.118	5.70E‐05
RZR60696.1	Siderophore iron transporter	−5.628	3.78E‐03
RZR61719.1	Ferric‐chelate reductase	−5.658	4.11E‐03
RZR66686.1	Glutamyl‐tRNA(Gln) amidotransferase	−6.049	4.79E‐04
RZR63736.1	Monocarboxylate permease‐like protein	−6.675	1.61E‐03
RZR61425.1	Hypothetical protein I1G_00001446	−6.766	1.26E‐06
RZR66909.1	Aldo/keto reductase	−9.653	9.66E‐11
RZR60251.1	Cysteine synthase B	−10.592	3.46E‐04
RZR60252.1	MFS drug transporter	−10.824	2.28E‐04
RZR69808.1	Symbiotic chitinase	−21.784	1.45E‐11
RZR58608.1	Hypothetical protein I1G_00008738	−26.011	2.10E‐07

Log_2_ fold change value for upregulated genes >2. Log_2_ fold change value for downregulated genes < −2.

**Table 2:** Differentially expressed genes in PcRKN treatment.

GenBank accession	Sequence description	Log_2_ fold change	Adj. *P*‐value
RZR69242.1	Floculation protein FLO1	7.501	5.75E‐11
RZR67544.1	Isochorismatase family protein	4.199	6.31E‐04
RZR68026.1	Putative som1 protein	3.157	3.60E‐04
RZR64799.1	Floculation protein FLO1	3.078	4.35E‐06
RZR59618.1	Hypothetical protein I1G_00011582	2.634	2.30E‐03
RZR64511.1	CRAL/TRIO domain protein	2.099	1.85E‐03
RZR63940.1	Ankyrin repeat protein	−2.148	1.35E‐03
RZR64281.1	Zinc finger, C2H2‐like protein	−2.605	1.09E‐04
RZR63781.1	Ribonuclease H‐like protein	−2.729	7.32E‐03
RZR64322.1	Hypothetical protein I1G_00000198	−2.934	1.35E‐03

Log_2_ fold change value for upregulated genes >2. Log_2_ fold change value for downregulated genes < −2.

**Table 3:** Differentially expressed genes in PcQRKN treatment.

GenBank accession	Sequence description	Log_2_ fold change	Adj. *P*‐value
RZR63795.1	Glycoside hydrolase family 75	9.036	5.45E‐15
RZR69242.1	Floculation protein FLO1	8.287	1.43E‐17
RZR61856.1	Glucokinase	8.287	5.87E‐18
RZR61845.1	Glycoside hydrolase family 75 protein	8.263	1.50E‐10
RZR62940.1	Glycoside hydrolase family 75	7.067	8.19E‐11
RZR61846.1	Major facilitator superfamily domain, general substrate transporter	6.368	2.80E‐17
RZR70395.1	Acid phosphatase	5.598	2.36E‐04
RZR65223.1	WSC domain‐containing protein	5.234	4.47E‐07
RZR70313.1	Chitosanase CSN1	5.180	1.53E‐09
RZR62842.1	FAD‐dependent monooxygenase	5.073	6.19E‐05
RZR63081.1	Glycoside hydrolase family 2 protein	4.696	6.21E‐10
RZR68451.1	Maltose permease	4.380	1.44E‐10
RZR68026.1	Putative som1 protein	4.259	1.37E‐08
RZR64799.1	Floculation protein FLO1	4.011	1.31E‐12
RZR70420.1	Floculation protein FLO1	3.419	2.62E‐03
RZR66026.1	Secreted aspartic proteinase precursor	3.373	4.89E‐04
RZR64948.1	Fungal chitosanase	3.260	1.31E‐05
RZR61625.1	Cytochrome P450 ClCP1	3.111	7.79E‐04
RZR68359.1	Nucleotide‐binding, alpha‐beta plait	3.097	5.56E‐05
RZR70289.1	Extracellular soluble lytic transglycosylase	3.002	1.26E‐09
RZR68649.1	Glycoside hydrolase family 75	2.988	1.91E‐03
RZR59618.1	Hypothetical protein I1G_00011582	2.929	7.41E‐05
RZR70338.1	Glycoside hydrolase, subgroup, catalytic core	2.862	2.22E‐03
RZR64159.1	Major facilitator superfamily domain, general substrate transporter	2.790	2.64E‐03
RZR64864.1	Hydrophobic surface binding protein A domain‐containing protein	2.686	3.46E‐03
RZR59400.1	Alpha‐l‐rhamnosidase A	2.672	7.69E‐03
RZR68019.1	Glycoside hydrolase, family 29	2.660	5.33E‐03
RZR59412.1	Cell wall protein	2.629	5.88E‐03
RZR65779.1	Aromatic‐ring hydroxylase‐like protein	2.565	7.38E‐03
RZR66703.1	Major facilitator superfamily domain, general substrate transporter	2.552	3.44E‐03
RZR70254.1	Thioredoxin‐like protein	2.543	3.53E‐04
RZR64866.1	Antigenic cell wall galactomannoprotein	2.518	5.77E‐03
RZR66682.1	Cell wall protein	2.456	5.88E‐03
RZR67148.1	Purple acid phosphatase‐lik	2.455	1.21E‐04
RZR59293.1	General substrate transporter	2.450	5.47E‐03
RZR63431.1	Peptidase S8/S53, subtilisin/kexin/sedolisin	2.405	7.09E‐03
RZR61740.1	Extracellular serine‐rich protein	2.381	3.98E‐03
RZR60307.1	Cytochrome P450	2.368	5.67E‐04
RZR69862.1	Cell wall galactomannoprotein	2.363	7.81E‐03
RZR66588.1	AtmA protein	2.350	3.02E‐07
RZR62045.1	EF‐hand calcium‐binding domain‐containing protein	2.198	5.47E‐03
RZR62051.1	Lipase 5	2.182	7.81E‐03
RZR65709.1	Exo‐beta‐d‐glucosaminidase	2.180	6.40E‐04
RZR62208.1	Peptidase A4 family protein	2.165	5.88E‐03
RZR59617.1	Galactose oxidase	2.145	1.27E‐07
RZR64865.1	Hydrophobic surface binding protein A domain‐containing protein	2.141	1.01E‐02
RZR65777.1	Related to glutathione S‐transferase GST‐6.0	2.112	1.30E‐02
RZR64511.1	CRAL/TRIO domain protein	2.096	3.05E‐04
RZR62042.1	Helix–loop–helix DNA‐binding protein	2.090	6.40E‐03
RZR67153.1	Hypothetical protein I1G_00011003	2.068	1.51E‐02
RZR62046.1	Polyketide synthase	2.054	7.69E‐03
RZR61204.1	Cell wall protein	2.037	1.19E‐02
RZR66605.1	Beta‐lactamase‐like protein	−2.001	8.76E‐03
RZR67311.1	Phosphoglycerate mutase family protein	−2.028	3.07E‐03
RZR67249.1	Hypothetical protein I1G_00007967	−2.046	1.32E‐03
RZR66003.1	Potassium channel	−2.047	3.80E‐03
RZR65332.1	Hypothetical protein I1G_00001300	−2.075	1.46E‐03
RZR58491.1	Hypothetical protein I1G_00003346	−2.088	6.40E‐03
RZR66328.1	S‐(hydroxymethyl)glutathione dehydrogenase	−2.090	7.81E‐03
RZR59616.1	Oxidoreductase	−2.097	8.76E‐03
RZR69857.1	Protein SERAC1	−2.104	1.23E‐03
RZR61435.1	Repetitive proline‐rich cell wall protein	−2.130	5.56E‐05
RZR61534.1	Trehalose synthase (Ccg‐9)	−2.148	9.96E‐10
RZR68989.1	Zn(2)‐C6 fungal‐type DNA‐binding domain protein	−2.149	8.76E‐03
RZR61511.1	Reductase	−2.153	5.91E‐04
RZR63940.1	Ankyrin repeat protein	−2.212	5.71E‐05
RZR63259.1	Hypothetical protein I1G_00011648	−2.240	6.80E‐04
RZR69039.1	DUF1929 multidomain protein	−2.336	2.91E‐03
RZR63453.1	Protein bli‐3	−2.365	5.70E‐03
RZR61033.1	Glucose repressible protein Grg1	−2.502	1.10E‐06
RZR68164.1	MARVEL‐like domain protein	−2.566	1.73E‐03
RZR66334.1	Protein kinase domain protein	−2.677	2.64E‐03
RZR62135.1	MFS transporter	−2.845	4.60E‐04
RZR59365.1	SUR7 protein	−2.876	2.57E‐04
RZR63781.1	Ribonuclease H‐like protein	−2.944	3.80E‐03
RZR66909.1	Aldo/keto reductase	−3.348	2.91E‐03

### Chitosan favours oxidation–reduction and associated processes in Pc

**Fig 2:**
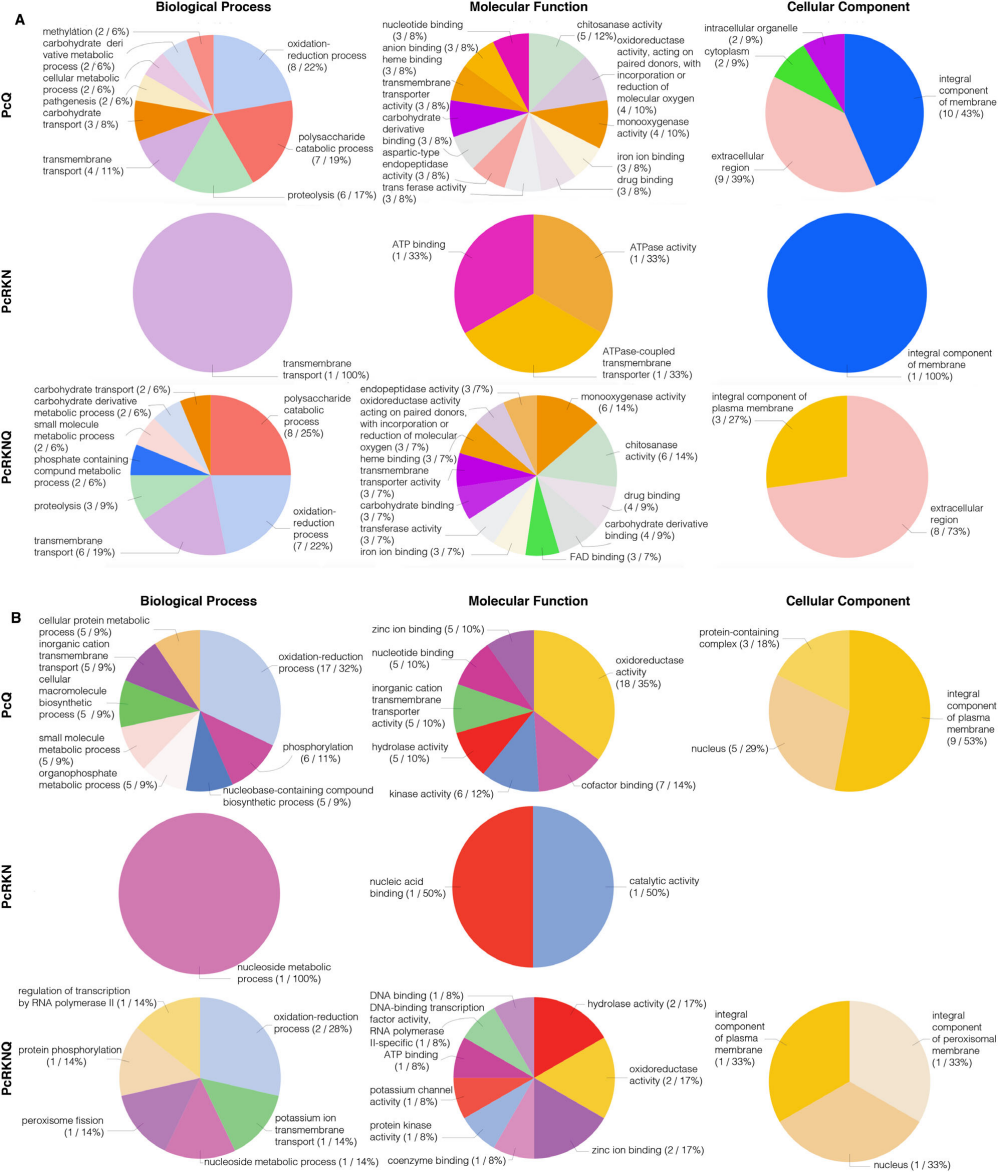
Chitosan favours redox processes in Pc. Gene ontology annotation of differentially expressed Pc genes with nematode eggs and chitosan using a threshold of ±2 in log_2_ fold change value. A. Upregulated genes, B. downregulated genes. Treatments: PcQ, *P*. *chlamydosporia* with chitosan (1 mg ml^−1^); PcRKN, *P*. *chlamydosporia* with *M*. *javanica* eggs (1 egg μl^−1^); PcRKNQ, *P*. *chlamydosporia* with *M*. *javanica* eggs (1 egg μl^−1^) and chitosan (1 mg ml^−1^). All treatments were applied for 4 days.

We assessed the sets of differentially expressed Pc genes for enrichment in Gene Ontology (GO) terms (Fig. 2). We considered terms from all three GO domains (Biological Processes, BP; Molecular Function, MF; Cellular Component, CC) for upregulated (Fig. 2A) and downregulated genes (Fig. 2B). For upregulated genes, ‘oxidation–reduction’ (GO:0055114) and ‘polysaccharide catabolism’ (GO:0000272) are the most enriched BP in chitosan treatments. The importance of oxidation–reduction and polysaccharide catabolism is also reflected in MF (e.g. ‘oxidoreductase activity acting on paired donors, with incorporation or reduction of molecular oxygen’ (GO:0016705) and ‘monooxygenase activity’ (GO:0004497) for oxidation–reduction processes; and ‘chitosanase activity’ (GO:0004568) for polysaccharide metabolism). Proteolysis (reflected in MF in ‘aspartic‐type endopeptidase’ (GO:0004190) and ‘endopeptidase’ (GO:0004175) activities) is also an enriched GO term in chitosan treatments. Transmembrane transport is a BP enriched in all treatments. ‘Carbohydrate transport’ (GO:0008643) and ‘carbohydrate derivative metabolic process’ (GO:1901135) are also enriched GO terms in chitosan treatments. Taken together, this would indicate a high chitosan turnover by Pc.

The presence of RKN represses nucleoside metabolic process (e.g. reflected in MF by ‘nucleic acid binding’ (GO:0003676), ‘catalytic activity’ (GO:0003824) and ‘DNA binding’ (GO:0003677)). These GO terms are enriched in both PcRKN and PcRKNQ treatments. ‘Oxidation–reduction process’ (GO:0055114) is the most enriched GO term for downregulated genes in chitosan treatments (reflected in MF by ‘oxidoreductase’ (GO:0016491) and ‘hydrolase’ (GO:0016787) activities). ‘Plasma membrane’ (GO:0005886) is the most enriched CC GO term for downregulated genes associated with chitosan treatments. PcRKN does not display any GO term associated with downregulated genes linked to CC GO domain.

### Chitosan and RKN significantly modify the expression of genes associated with energy, lipid and chitosan catabolism and proteolysis

**Fig 3:**
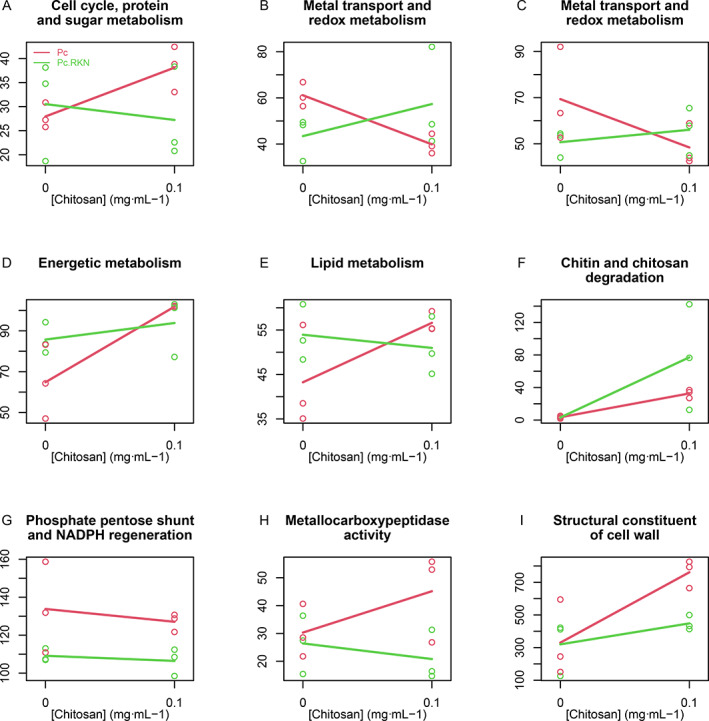
Chitosan and RKN modify the expression of genes associated to 113 GO terms. A cluster of the median profile of 113 GO terms analysis generated nine groups. These GOs are enriched and show a significant behaviour. A. Cluster related to cell cycle and associated processes. B. Redox and transport. C. Redox and transport metabolism. D. Includes GO terms related to the previous step to energy production. E. Lipid metabolism. F. All GO terms related to chitin/chitosan degradation. G. Phosphate pentose shunt and NADPH regeneration GO terms. H. This cluster is represented by one GO term: metallocarboxypeptidase activity. I. Associated with cell wall–associated processes. Legend is shown in Fig. 3A.

Statistical analyses of GO terms associated with Pc DEG show 113 GOs enriched in all treatments (Supplementary Table 1). These can be classified into nine clusters according to their behaviour with chitosan (Fig. 3). Pc genes included in these significantly enriched GO terms are shown in Supplementary Table 2. Behaviour of the individual GO term is shown in Supplementary Fig. 2. Chitosan promotes the expression of Pc genes associated with 38 GO terms (Fig. 3A) when nematodes are absent. Twenty‐four of them are related to the cell cycle, 12 to protein synthesis and modification and two to sugar metabolism. In the absence of nematodes, chitosan represses Pc genes involved in metal transport and redox metabolism GO terms (Fig. 3B and C; Supplementary Table 1). Nematode eggs reverse this behaviour. Conversely, chitosan induces genes associated with energy GO terms (Fig. 3D). Chitosan also induces the expression of genes associated with lipid metabolism GO terms (specially sphingomyelin (GO:0004767, GO:0006684 and GO:0006685) metabolism; Fig. 3E). Chitosan increases expression of genes in GO terms related to chitin and chitosan degradation (‘chitosanase activity’ (GO:0016977) and ‘exo‐1,4‐beta‐d‐glucosaminidase’ activity (GO:0052761)). This is further enhanced when RKN eggs are present (Fig. 3F). ‘Phosphate pentose shunt’ (GO:0006098) and ‘NADPH regeneration’ (GO:0006740) (Fig. 3G) are not affected by chitosan. Both are repressed by the presence of nematodes. ‘Metallocarboxypeptidase activity’ (GO:0004181) is overexpressed with chitosan in the absence of nematode eggs (Fig. 3H). ‘Structural constituent of cell wall’ (GO:0005199) is induced with chitosan (Fig. 3I).

### Chitosan and RKN significantly modify Pc gene expression dynamics

**Fig 4:**
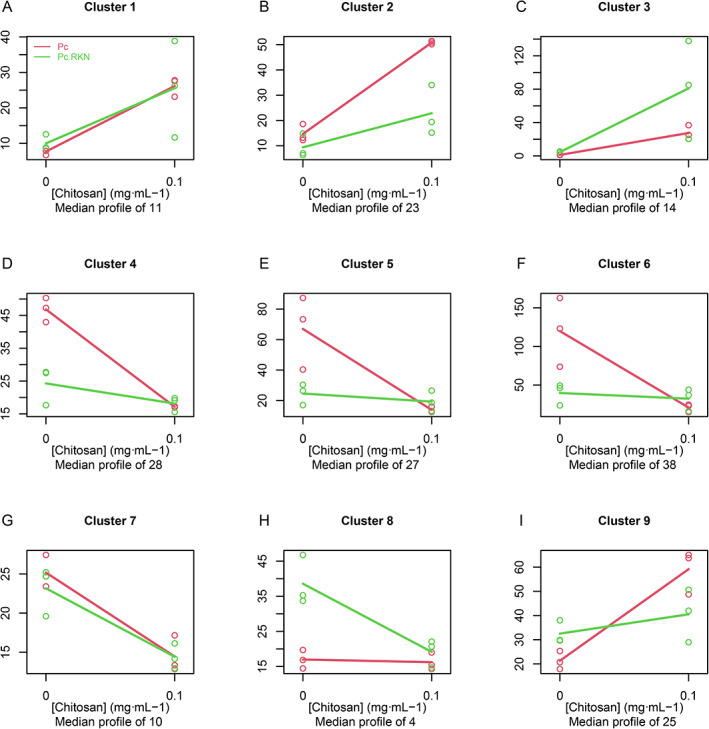
Chitosan and RKN significantly modify the expression of 180 genes. A median profile of 180 genes clustered in nine groups according to their trends with chitosan. Legend is shown in Fig. 4A.

In response to nematodes and chitosan, 180 Pc genes cluster into nine significant unique gene trends (Fig. 4, Supplementary Table 3). Individual gene behaviour is shown in Supplementary Fig. 3. Chitosan activates the expression of the genes associated with clusters 1–3 (Fig. 4A, B and C). Cluster 1 includes, among others, genes encoding proteins related with reactive oxygen species (ROS) metabolism, such as cytochrome P450 ClCP1 (RZR61625.1) and thioredoxin‐like protein (RZR70254.1). ROS metabolisms play a key role in fungal response to chitosan inducing oxidative metabolism in chitosan‐sensitive fungi such as *Neurospora crassa* (Lopez‐Moya *et al*., 2016). In Cluster 2, the presence of nematodes mitigates the increase in gene expression. This can be confirmed in Tables 1 and 3. Genes encoding peptidases (RZR60329.1, RZR61987.1, RZR62018.1, RZR62208.1, RZR62805.1, RZR63158.1, RZR63431.1, RZR66026.1 and RZR69491.1) exhibit this behaviour. RKN and chitosan induce gene expression in Cluster 3. This cluster includes genes encoding chitosanases (RZR61845.1, RZR62940.1, RZR63795.1, RZR64948.1 and RZR70313.1), adhesives (FLO1; RZR64799.1 and RZR69242.1) and sugar catabolism proteins (RZR61856.1, RZR63081.1, RZR65709.1 and RZR66181.1). Differential expression of genes in this cluster may explain why chitosan increases RKN egg parasitism by Pc, since chitosanases and polysaccharide degrading enzymes are involved in RKN egg parasitism (Aranda‐Martinez *et al*., 2016) and chitosan assimilation. Genes in Clusters 4–6 (Fig. 4D, E and F) are repressed with chitosan when nematodes are absent. When nematodes are present chitosan does not modify gene expression dynamics. Cluster 7 (Fig. 4G) includes genes repressed by chitosan regardless of the presence of nematodes. Some membrane transporters show this trend. Nematode eggs increase expression of genes in Cluster 8 (Fig. 4H), but chitosan represses them. The most significant encodes a salicylate hydroxylase (RZR59707.1). Nematode eggs and chitosan induce the expression of genes in Cluster 9 (Fig. 4I). Genes encoding the following proteins belong to this cluster: RZR65223.1 WSC domain‐containing protein, RZR69865.1 Cytochrome P450, RZR64444.1 GMC oxidoreductase, RZR64079.1 3‐beta hydroxysteroid dehydrogenase/isomerase. These genes would play a crucial role for RKN egg parasitism by Pc with chitosan.

### Eight Pc genes are expressed in endophytism, pathogenicity and response to chitosan

**Fig 5:**
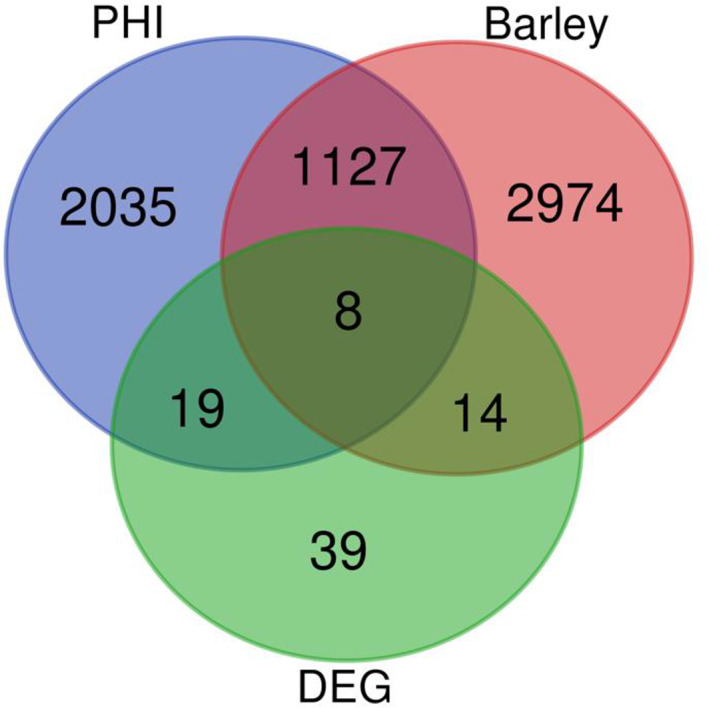
Eight Pc genes are common to endophytism, pathogenicity and chitosan response. Venn diagram showing the intersection of genes expressed when Pc colonizes barley (Larriba *et al*., 2014), PHI database data and differentially expressed genes (DEG; log_2_ fold change >2) in this study. Eight genes are found common to all studies.

To investigate Pc genes shared between endophytism and parasitism, we combined PHI‐base (Urban *et al*., 2020; www.phi-base.org), Pc upregulated genes found in this work (RKN parasitism and chitosan metabolism) and Pc genes expressed during Barley colonization (Larriba *et al*., 2014). The intersection between these datasets includes eight genes encoding the following proteins (Fig. 5): RZR66026.1 (secreted aspartic proteinase precursor, overexpressed in PcQ and PcRKNQ), RZR62042.1 (helix–loop–helix DNA‐binding protein, overexpressed in PcRKNQ), RZR63158.1 (peptidase A1, overexpressed in PcQ), RZR64159.1 (major facilitator superfamily domain, general substrate transporter, overexpressed in PcRKNQ), RZR62046.1 (polyketide synthase (PKS), overexpressed in PcRKNQ), RZR61625.1 (cytochrome P450 ClCP1, overexpressed in PcQ and PcRKNQ), RZR69240.1 (APSES transcription factor, overexpressed in PcQ) and RZR61845.1 (glycoside hydrolase family 75 protein (chitosanase) overexpressed in PcQ and PcRKNQ). We have investigated the expression of these genes in banana plants colonized by Pc. Banana plants modify the expression of these Pc genes (Supplementary Fig. 4). Both barley and banana are monocots, and the former has been widely used for endophytism studies (Maciá‐Vicente *et al*., 2009; Murphy *et al*., 2014a; Murphy *et al*., 2014b; Larriba *et al*., 2015).

Pc acidic peptidases modify their expression when banana plants are present. Secreted aspartic proteinase precursor gene is induced, while the A1 peptidase gene is slightly repressed. A chitosanase gene is expressed whenever chitosan is present. The fungus does not modify the expression of this gene when it grows endophytically on bananas, but the maximum expression occurs in the ‘artificial rhizosphere’ (liquid surrounding roots) with chitosan. One Pc PKS protein, involved in the secondary metabolism of the fungus, is slightly induced by the presence of the plant. Secondary metabolism plays an essential role during plant–host interaction (Macheleidt *et al*., 2016). Endophytism in Pc also modifies the expression of transcription factors. Helix–loop–helix DNA binding protein is overexpressed in all treatments. Cytochrome P450 ClCP1 is related to oxidative metabolism, which is also involved in this process.

## Discussion

In this work, we have carried out a transcriptomic study, using RNA‐seq, to determine Pc genes involved in the response to chitosan and RKN parasitism. We have found that chitosan on its own modifies the expression of more Pc genes than RKN does. This may be because chitosan permeabilizes plasma membrane in fungi triggering the activation of reactive oxygen species (ROS) and cell death (Lopez‐Moya *et al*., 2019). Besides, chitosan solutions reach all fungal cells, whereas nematodes are in contact with only specific parts (mainly appressoria) of the fungus. Chitosan enhances redox processes, proteolysis and carbohydrate metabolism in Pc. Redox metabolism is the most affected process by chitosan. To this respect, the gene encoding CipC1 (concanamycin induced protein C), a ROS‐related protein, is the most induced by chitosan. This protein has also been found induced in citrus fruits infected by the fungal pathogen *Penicillium digitatum* (López‐Pérez *et al*., 2015). CipC1 is involved in hyphal branching and upregulated in *Laccaria bicolor* in response to *Pseudomonas fluorescens* (Deveau *et al*., 2007). This gene could be overexpressed in Pc to overcome chitosan‐induced ROS. Other genes overexpressed in chitosan‐induced ROS are Cytochromes P450 and b561, l‐ascorbate oxidase and aldo/keto reductase. This enhanced activation of redox metabolism–related genes connects resistance with chitosan‐sensitive fungi (Jaime *et al*., 2012; Lopez‐Moya *et al*., 2016). Pc has to fight plasma membrane oxidation generated by chitosan‐induced ROS. Chitosan permeabilizes the plasma membrane of fungi in an energy‐dependent manner (Palma‐Guerrero *et al*., 2009). In our work, Pc activates energy and lipid metabolism genes with chitosan. This was previously found on chitosan‐sensitive yeasts and filamentous fungi (Jaime *et al*., 2012; Lopez‐Moya *et al*., 2015; Lopez‐Moya *et al*., 2016). Lipid oxidation generates oxylipins (Gabbs *et al*., 2015), which can modify fungal morphology, promoting appressoria differentiation (Niu *et al*., 2020). We then may speculate that Pc oxylipins could be involved in chitosan induction of Pc appressoria found previously (Escudero *et al*., 2016). ROS increase in the cell could lead to Pc methylation (Wu and Ni, 2015), a highly represented GO term in chitosan treatments. This indicates chitosan may also trigger Pc epigenetic mechanisms (Razin and Cedar, 1991; Phillips, 2008). ROS also causes damage to proteins, probably activating protease induction (Schieber and Chandel, 2014). To this respect, chitosan induces Pc genes encoding all families of peptidases. In our study, chitosan enhances Pc subtilisin S8/S53 expression. This protease may help the fungus to adapt to the ecological niches, facilitating nutrition (Li *et al*., 2017). Pc subtilisins have been detected in both the parasitic (Escudero *et al*., 2016) and endophytic (Lopez‐Llorca *et al*., 2010) phases of the fungal lifestyle. Peptidases A1 and A4 (fungal family G1), involved in plant parasitism and host adaptation (Kirshnan *et al*., 2018), are also upregulated in Pc with chitosan. Finally, Pc metallo‐endopeptidases are also upregulated with chitosan. This wide range of biological processes and molecular functions affected by chitosan indicates the effort made by the cellular machinery of Pc to overcome stress. Further work should quantify the response of individual Pc cells against chitosan, since antibiotics generate dead, dying and resistant active and inactive cells (Bamford *et al*., 2017).

Pc has mechanisms to efficiently degrade chitosan and thus reduce its damage (mainly caused by ROS) on cells. Pc overexpresses chitosanases (GH75) with chitosan, degrading it into monosaccharides. One WSC domain‐containing protein, a stress responsory related to carbohydrate binding (Tong *et al*., 2016; Oide *et al*., 2019), is also upregulated with chitosan. Genes coding for membrane transporters, such as the major facilitator superfamily domain, general substrate transporter, oligopeptide transporter OPT‐like protein, maltose permease and sugar transporter family protein, are also overexpressed with chitosan. They may be involved in the assimilation of monosaccharides generated upon chitosan degradation by Pc. *Neurospora crassa*, a chitosan‐sensitive fungus, also shows the activation of monosaccharide transport genes (Lopez‐Moya *et al*., 2016) upon chitosan treatment but not as many as Pc. This may be a further reason to explain why Pc is more resistant to chitosan than *N*. *crassa*. Overexpression of Pc glucokinase in the presence of chitosan is probably related to the final catabolism of this polymer (Maitra and Lobo, 1983). Other glycoside hydrolases (GH2 and 3) are also overexpressed in chitosan treatments. This may indicate the high metabolic potential of Pc to degrade and assimilate chitosan.

RKN eggs stimulate Pc to overexpress rather than repress genes. RKN also induce gene expression of nematode‐trapping fungi, such as *Arthrobotrys conoides* (Pandit *et al*., 2017), indicating the activation of parasitic pathways. In this work, the addition of chitosan to nematode eggs displays the highest values of Pc gene expression in all treatments tested. This shows that chitosan is a strong elicitor of genes potentially involved in RKN egg parasitism by Pc. In cluster analyses, genes encoding proteins for adhesion (FLO1), chitosan and sugar degradation (GH2, GH3, GH75), membrane transport (MFS‐transporters) and carbohydrate metabolism (glucokinase) are overexpressed in chitosan and RKN treatments. In a previous study (Lin *et al*., 2018), RKN eggs in Minimal Medium (MM) were found to induce Pc adhesives (CFEM), GHs enzymes and proteases. In our GO cluster analysis, we have found GO terms related to chitin/chitosan degradation with an enhanced expression upon addition of nematode eggs and chitosan. Genes that share this behaviour may explain why RKN eggs parasitism by Pc increases in the presence of chitosan. The trend pattern (increase or decrease in expression by adding chitosan to treatments) is best observed in a gene rather than in GO cluster analyses. This is because GOs share genes and when some have opposing trends, the average GO behaviour does not show up in the cluster analysis. Pc deploys its machinery to putatively attach to RKN eggshell by binding peptides, lipids and carbohydrates. FLO1 proteins are flocculation proteins present in yeasts, related to adhesion to hyphae (Moreno‐García *et al*., 2018). FLO1 is a mannose‐binding glycoprotein, which could be a determinant for hyphal adhesion to the nematode eggshell. A CRAL/TRIO domain protein is upregulated when Pc is in contact with RKN. This domain is related to the binding to small lipophilic molecules (Panagabko *et al*., 2003). It could also be involved to the attachment to the eggshell lipid layer (Johnston and Dennis, 2012). We hypothesize that once the fungus is attached to the eggshell, its degradation begins by transforming the RKN egg chitin layer (Johnston and Dennis, 2012) to chitosan using chitindeacetylases (Aranda‐Martinez *et al*., 2016) and degrading the resulting chitosan mainly using chitosanases (GH75). Other glycosyl hydrolases (GH2 and GH3) may also contribute to degradation. Sugars may be introduced to the cells of the fungus through transporters (MFS). All molecular processes involved in egg parasitism are enhanced by the addition of chitosan. The increase in ROS could explain this behaviour.

Pc can act both as an endophyte (Bordallo *et al*., 2002) or RKN egg parasite (Lopez‐Llorca *et al*., 2002). In this work, we have explored the common ‘gene toolbox’ involved in endophytism (Larriba *et al*., 2014), pathogenicity (PHI‐base) and response to chitosan. We have found eight candidate genes, among them proteases, chitosanases, redox‐related proteins and transcription factors.

Proteases modify their expression in endophytic processes. This is related to parasitism in other fungi (Druzhinina *et al*., 2012) and to endophytism in insect pathogenic fungi (Moonjely *et al*., 2016). In works of gene expression during endophytism, it has been found that Pc overexpresses ribosomal proteins, proteases, secreted proteins and heat shock proteins, among others probably related to transitions in the lifestyle of the fungus (Pentimone *et al*., 2019). The fact that chitosan increases the secretion of Pc proteases could explain why the fungus colonizes the plant efficiently when chitosan is present. Pc does not modify the expression of GH75 when it grows endophytically on bananas, but the maximum expression of this gene occurs in the rhizosphere with chitosan. Endophytic fungi have enzymes that modify chitin and chitosan (Govinda Rajulu *et al*., 2011; Venkatachalam *et al*., 2015). This could mean that the fungus enhances its chitin‐ and chitosanolytic metabolism in order to start plant root colonization. Chitosan and its derivatives are highly related to redox metabolism (Sarangapani *et al*., 2018; Ivanova and Yaneva, 2020), as we commented before. Cytochrome P450 ClCP1 is also involved in oxidative metabolism (Korzekwa, 2014), and it is expressed in endophytes (Chadha *et al*., 2018). This is consistent with studies which prove that root colonization processes activate ROS (Segal and Wilson, 2018). One Pc PKS protein is slightly induced by the presence of the plant. PKS are expressed in biocontrol fungus such as *Clonostachys rosea* during fungal–fungal interactions (Fatema *et al*., 2018) and virulence events (Tsai *et al*., 1998). This gene is also related to melatonin biosynthesis (Knapp *et al*., 2018). Melatonin is a precursor of plant hormones (Arnao and Hernández‐Ruiz, 2018). It has been shown that endophytic organisms secrete melatonin and derivatives during root colonization (Jiao *et al*., 2016). This could mean that Pc is secreting metabolites homologous to plant hormones in order to facilitate plant root colonization. This could be one of the reasons why Pc increases banana plant growth and development (Mingot‐Ureta *et al*., 2020). Finally, endophytism in Pc modifies the expression of transcription factors. Helix–loop–helix DNA binding protein is overexpressed in all treatments. Related to previous results, Pc seems to be activating metabolic routes in order to colonize the plant properly hiding from plant defences.

In conclusion, Pc modifies its gene expression to parasite RKN eggs, colonize plant roots or resist chitosan. When Pc is growing with chitosan, it activates metabolic pathways to avoid chitosan‐induced ROS damage. Pc enzymes capable of degrading it into sugars. Pc sugar carriers introduce them into the cell for catabolism. This process increases ROS content, and Pc activates lipid metabolism. Metal membrane transporters putatively carry out these reduction and oxidation reactions. During this process genes related to plant endophytism or nematode egg parasitism are activated, which could indicate that, in combination with the real stimulus, chitosan could be a non‐toxic additive to increase plant colonization by Pc and sustainably reduce plant‐parasitic nematodes, such as RKN, in banana and other agroecosystems.

## Experimental procedures

### Fungi, chitosan, nematodes and plants

*Pochonia chlamydosporia* var. *chlamydosporia* (=*Metacordyceps chlamydosporia* var. *chlamydosporium*) isolate 123 (Pc) (ATCC No. MYA‐4875; CECT No. 20929) was used in this study. Pc was obtained from *Heterodera avenae*–infected eggs (Olivares and López‐Llorca, 2002). Chitosan T8 (70 kDa and 80.5% deacetylation degree) was obtained from Marine Bioproducts GmbH (Bremerhaven, Germany). Chitosan solutions were prepared as described in Palma‐Guerrero *et al*. (2010). *Meloidogyne javanica* was a kind gift from Dr. Caridad Ros (IMIDA, Murcia, Spain). It was maintained in tomato susceptible plants (*Solanum lycopersicum* Mill cv. Marglobe). RKN egg masses were hand‐picked from infected tomato roots and surface‐sterilized with 1% sodium hypochlorite as in McClure *et al*. (1973). *Meloidogyne javanica* eggs in all developmental stages were used for experiments. One‐month‐old *in vitro* banana plantlets (*Musa acuminata* cv. Dwarf Cavendish) were purchased from Cultesa S.A. (Tacoronte, Canary Islands, Spain).

### Pc, chitosan and RKN: RNA‐seq experimental design

Pc conidia (final concentration 10^6^ conidia·ml^−1^) were inoculated into 100 ml flasks each containing 20 ml Czapek Dox broth medium (Ward *et al*., 2012). Flasks were incubated at 25°C with shaking at 120 rpm. After 5 days, mycelia were recovered by filtration through Miracloth (Calbiochem) and washed twice with sterile distilled water. Pc mycelia (ca. 0.2 g) were inoculated axenically into 100 ml flasks each containing 20 ml MM (Aranda‐Martinez *et al*., 2016) and amended with either: (i) chitosan (PcQ) (0.1 mg ml^−1^ final concentration), (ii) surface‐sterilized RKN eggs (PcRKN) (1 egg μl^−1^ final concentration), or (iii) both (PcRKNQ). Controls consisted of Pc mycelium growing in MM (Pc). All treatments were carried out in triplicate. Flasks were incubated for 4 days as before. Samples were then filtered through Miracloth, frozen in liquid N_2_, lyophilized and stored at −80°C until used. The experiment was performed three times.

### RNA extraction and RNA‐seq performing

Total RNA extractions were performed using TRIzol reagent (Life Tech), following the manufacturer's protocol. The quality of all RNA samples was determined using a bioanalyzer (Agilent 2100 Bioanalyzer System) to confirm it was adequate for RNA‐seq analysis (Supplementary Table 4). Three replicates per treatment were then selected at random to perform RNA‐seq analysis. cDNA synthesis, library construction and Illumina sequencing were carried out by Macrogen (Seoul, South Korea). TruSeq Stranded mRNA LT Sample Prep Kit (Illumina) was used as Library Kit and TruSeq Stranded mRNA Sample Preparation Guide, Part #15031047 Rev. E as Library Protocol. The reagent used was NovaSeq 6000 S4 Reagent Kit (Illumina) and sequencing protocol NovaSeq 6000 System User Guide Document #1000000019358 v02.

### Bioinformatic analyses

Raw reads were trimmed and filtered with Trimmomatic (Bolger *et al*., 2014) to remove adapters with up to two mismatches that had a palindrome read alignment accuracy of 30 and a sequence match accuracy of 10. Leading and trailing low‐quality or N bases (<3) were removed by using a 4‐base‐wide sliding window. Where average base quality was low (<15) reads were trimmed and any short reads (<80 bp) were removed. The quality of the reads was then checked with FASTQC (http://www.bioinformatics.babraham.ac.uk/projects/fastqc/).

Reads were quantified using Salmon mapping against the *P*. *chlamydospora* 123 genome (GenBank accession: GCA_000411695.2) using a wrapper script (align_and_estimate_abundance.pl) from the Trinity software package (Grabherr *et al*., 2011). DEG were determined with the Bioconductor package DESeq2 (Love *et al*., 2014) using the likelihood ratio test. Genes were filtered so only those with at least three samples with >10 counts were analysed. *P* values were adjusted using the Benjamini–Hochberg (BH) correction (Benjamini and Hochberg, 1995). Genes were considered differentially expressed if they had a log_2_ fold change of ±2 and a BH‐FDR‐adjusted *P*‐value of ≤0.05. This process selected genes whose expression varied the most with treatments with respect to the control and characterized the main genes involved in the response of Pc to chitosan and RKN.

A consensus set of transcripts was functionally annotated with GO terms using Blast2GO (http://www.blast2go.com/b2ghome) (Edgar *et al*., 2002). Protein sequences were annotated using the InterPro (http://www.ebi.ac.uk/interpro) and KEGG databases (http://www.genome.jp/kegg/pathway.html) in OmicsBox (BioBam, Spain; http://www.biobam.com/omicsbox). Significant differential gene expression between treatments was analysed using maSigPro R package (Nueda *et al*., 2014). And significant differential GOs were obtained with maSigFun, an adaptation of maSigPro for dealing with groups of genes, as in Lopez‐Moya *et al*. (2016).

### Pc‐Chitosan‐*Musa* : bioassays

Pc (10^6^ conidia·ml^−1^) was inoculated in Cz liquid medium and incubated for 5 days as described above. Thirty‐six banana plantlets were placed individually in Magenta Boxes™ (Sigma) each containing 50 ml of MM supplemented or without chitosan (final concentration 0.1 mg ml^−1^). Half of the plants were inoculated with 0.2 g Pc mycelium and half were left uninoculated. Plants were maintained at 24°C, 60% relative humidity and 16:8 h light/darkness photoperiod, with 100 rpm shaking, for 4 days. Control replicates of 0.2 g of mycelium were inoculated in 100 ml flasks each containing 20 ml MM supplemented or not with chitosan (final concentration 0.1 mg ml^−1^). Controls with and without chitosan were made by triplicate to obtain replicability among the samples. To extract RNA, three plant roots from the same treatment were collected for each extraction. In this way, three replicates were obtained per treatment, each replicate with three whole roots from three different plants. RNA was extracted as described above. Final treatments were: Pc (Pc mycelium in MM), PcQ (Pc mycelium in MM amended with 0.1 mg ml^−1^ chitosan), PcB (Pc mycelium growing in MM close to banana roots), PcBQ (Pc mycelium growing in MM amended with 0.1 mg ml^−1^ chitosan close to Banana roots), BPc (banana roots colonized by Pc) and BPcQ (Banana roots colonized by Pc in medium amended with 0.1 mg ml^−1^ chitosan). To identify potential genes related to endophytism and pathogenicity, a whole‐genome blast search was conducted against the Pathogen–Host Interaction database v. 4.9 (PHI‐base; www.phi-base.org; Urban *et al*., 2020). These candidate genes were intersected with expressed genes when Pc colonizes Barley (Larriba *et al*., 2014) and upregulated genes (log_2_ fold change values ≧2) in all treatments in this work (data available in Supplementary Information File). Selected genes were evaluated by qRT‐PCR in banana treatments.

### qRT‐PCR

RNA was treated twice with DNase (Turbo DNA‐free, Ambion) to remove any remnants of DNA in the samples. Then, cDNA was obtained using NZY First Strand cDNA Synthesis Kit (NZYtech). Finally, qRT‐PCRs were performed in a StepOnePlus™ Real‐Time PCR System machine, using SYBR Green with ROX (Roche) and a ΔΔCt methodology. qRT‐PCR analyses included three biological replicates with three technical replicates each. ∆∆Ct method was used to calculate the relative fold gene expression of samples and statistical analyses were performed using ANOVA in GraphPad Prism 7.0 Software (www.graphpad.com). Primers used for qRT‐PCRs are shown in Supplementary Table 5. *Pochonia chlamydosporia* allantoate permease (RZR69578.1; Rosso *et al*., 2014), glyceraldehyde‐3‐phosphate dehydrogenase (RZR61537.1; Escudero *et al*., 2016) and ß‐tubulin (RZR65128.1; Ward *et al*., 2012) were used as housekeeping genes.

### Statistical analyses and figures

Statistical analysis for differential gene expression was determined using DESeq2 (Love *et al*., 2014). Statistics for gene set enrichment analysis were performed using OmicsBox (BioBam, Spain) and figures generated using GraphPad Prism version 7.00 for Mac, GraphPad Software, La Jolla, California, USA, (www.graphpad.com).

## Author Contributions

L.V.L.‐L. conceived the original screening and research plans and supervised original writing; F.L.‐M. supervised the experiments and provided technical assistance; M.S.‐F. performed biological experiments and original writing; C.S. and D.J.S. performed bioinformatic analyses; M.J.N. performed analyses with maSigPro; all authors completed the writing; M.S.‐F. agrees to serve as the author responsible for contact and ensures communication.

## Supporting information


**Appendix S1.** Supporting Information.

**Supplementary Fig. 1.** Confirmation of data replicability by qRT‐PCR. A, q‐PCR analysis. B, RNA‐seq log2 fold change data.

**Supplementary Fig. 2.** Individual trends of 113 GO terms included in clusters in Fig. 3.

**Supplementary Fig. 3.** Individual trends of 180 genes included in clusters in Fig. 4.

**Supplementary Fig. 4.** Pc gene expression of 8 selected genes when the fungus colonizes banana roots. Treatments: Pc (Pc mycelium in MM), PcQ (Pc mycelium in MM amended with 0.1 mg·mL^−1^ chitosan), PcB (Pc mycelium growing in MM close to banana roots), PcBQ (Pc mycelium growing in MM amended with 0.1 mg·mL^−1^ chitosan close to banana roots), BPc (banana roots colonized by Pc) and BPcQ (banana roots colonized by Pc in medium amended with 0.1 mg·mL^−1^ chitosan).

**Supplementary Fig. 5.** Workflow of the steps followed for the analysis and obtaining log2 fold change values from the raw data.

**Supplementary Table 1.** Statistical analyses of 113 GO enriched Terms from Fig. 3.

**Supplementary Table 2.** 113 GO enriched Terms from Fig. 3: ID, Description and genes included.

**Supplementary Table 3.** Classification and statistics of the 180 genes represented in clusters in Fig. 4.

**Supplementary Table 4.** Quality of RNA extracted. All samples were sent to Macrogen to perform RNA‐seq analyses.

**Supplementary Table 5.** Primers used in all gene expression analyses.
