# Supplementary material for: Chitosan modulates Pochonia chlamydosporia gene expression during nematode egg parasitism
Source: Environ Microbiol. 2021 Feb 5;23(9):4980–97. doi: 10.1111/1462-2920.15408 (PMC8518118; doi:10.1111/1462-2920.15408)
Supplement: Supplementary file 2 — Supplementary Fig. 1. Confirmation of data replicability by qRT‐PCR. A, q‐PCR analysis. B, RNA‐seq log2 fold change data. [file EMI-23-4980-s001.docx]

**Supplementary Figure 1.** Confirmation of data replicability by qRT-PCR. A, q-PCR analysis. B, RNAseq Log2 fold change data.
