# Supplementary material for: Chitosan modulates Pochonia chlamydosporia gene expression during nematode egg parasitism
Source: Environ Microbiol. 2021 Feb 5;23(9):4980–97. doi: 10.1111/1462-2920.15408 (PMC8518118; doi:10.1111/1462-2920.15408)
Supplement: Supplementary file 3 — Supplementary Fig. 2. Individual trends of 113 GO terms included in clusters in Fig. 3. [file EMI-23-4980-s003.pdf]

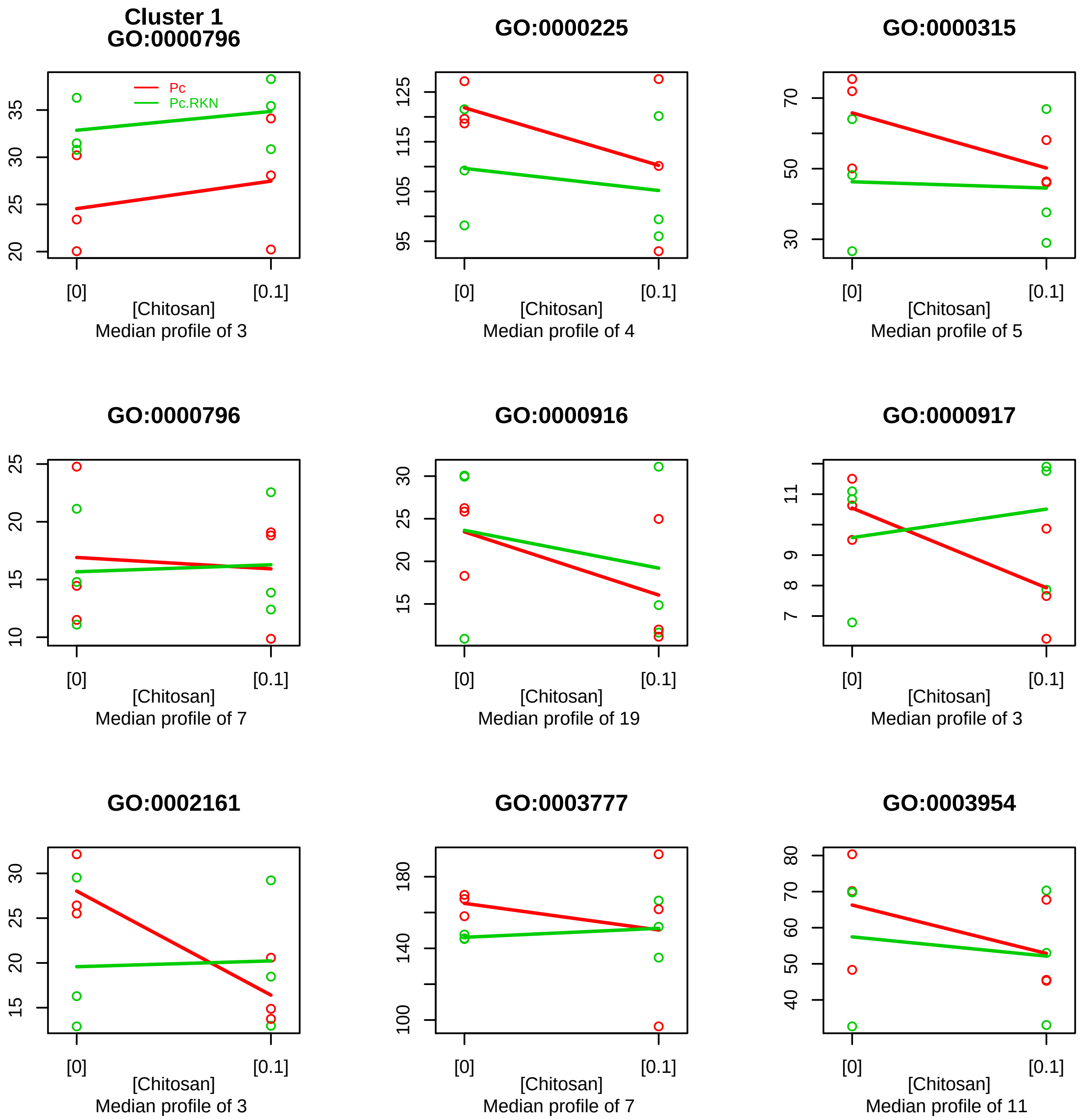

Cluster 1
GO:0000796
Pc
Pc.RKN
[0]
[0.1]
[Chitosan]
Median profile of 3
GO:0000225
[0]
[0.1]
[Chitosan]
Median profile of 4
GO:0000315
[0]
[0.1]
[Chitosan]
Median profile of 5
GO:0000796
[0]
[0.1]
[Chitosan]
Median profile of 7
GO:0000916
[0]
[0.1]
[Chitosan]
Median profile of 19
GO:0000917
[0]
[0.1]
[Chitosan]
Median profile of 3
GO:0002161
[0]
[0.1]
[Chitosan]
Median profile of 3
GO:0003777
[0]
[0.1]
[Chitosan]
Median profile of 7
GO:0003954
[0]
[0.1]
[Chitosan]
Median profile of 11

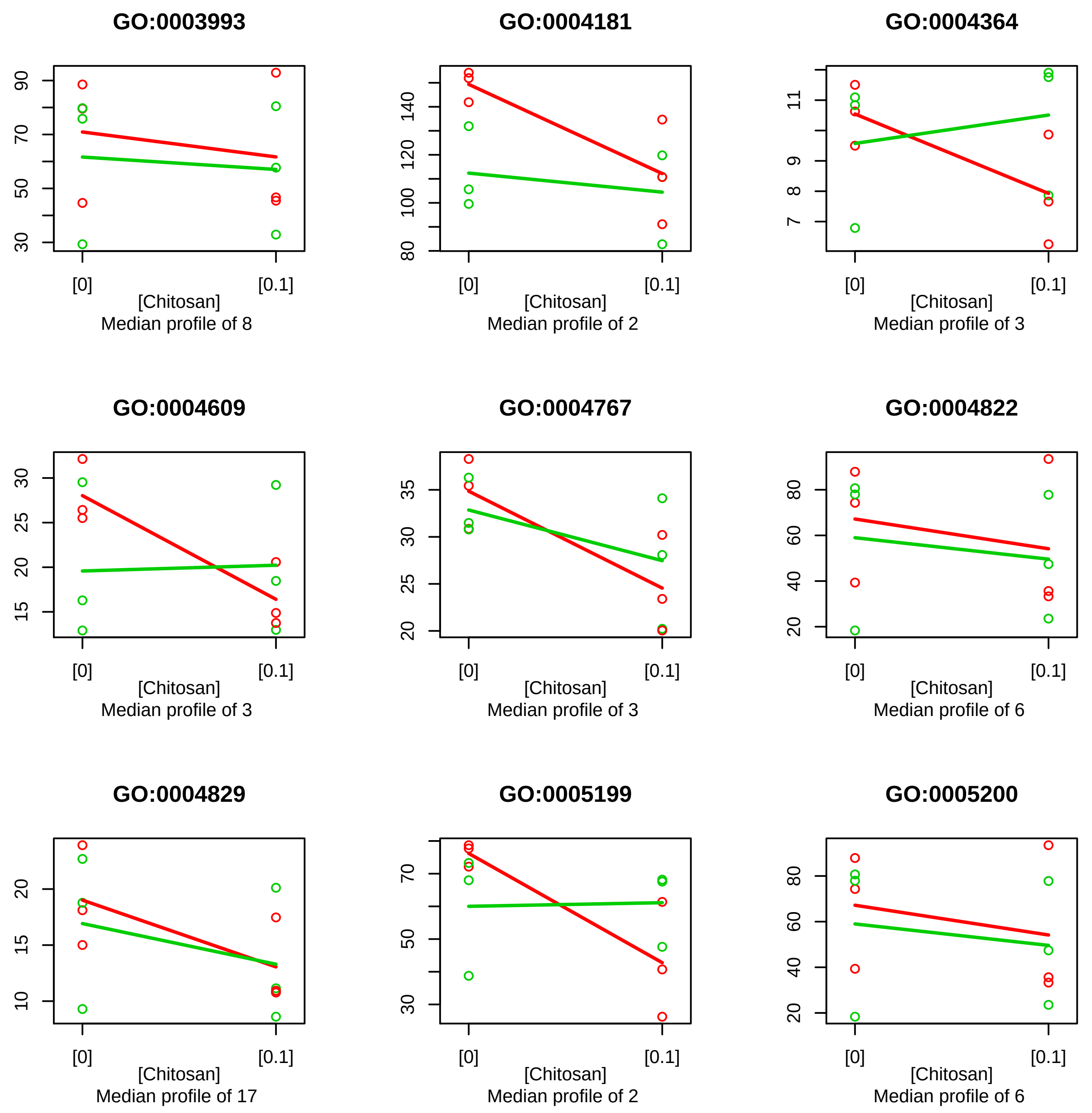
GO:0003993
Median profile of 8
GO:0004181
Median profile of 2
GO:0004364
Median profile of 3
GO:0004609
Median profile of 3
GO:0004767
Median profile of 3
GO:0004822
Median profile of 6
GO:0004829
Median profile of 17
GO:0005199
Median profile of 2
GO:0005200
Median profile of 6
[0]
[0.1]
[Chitosan]

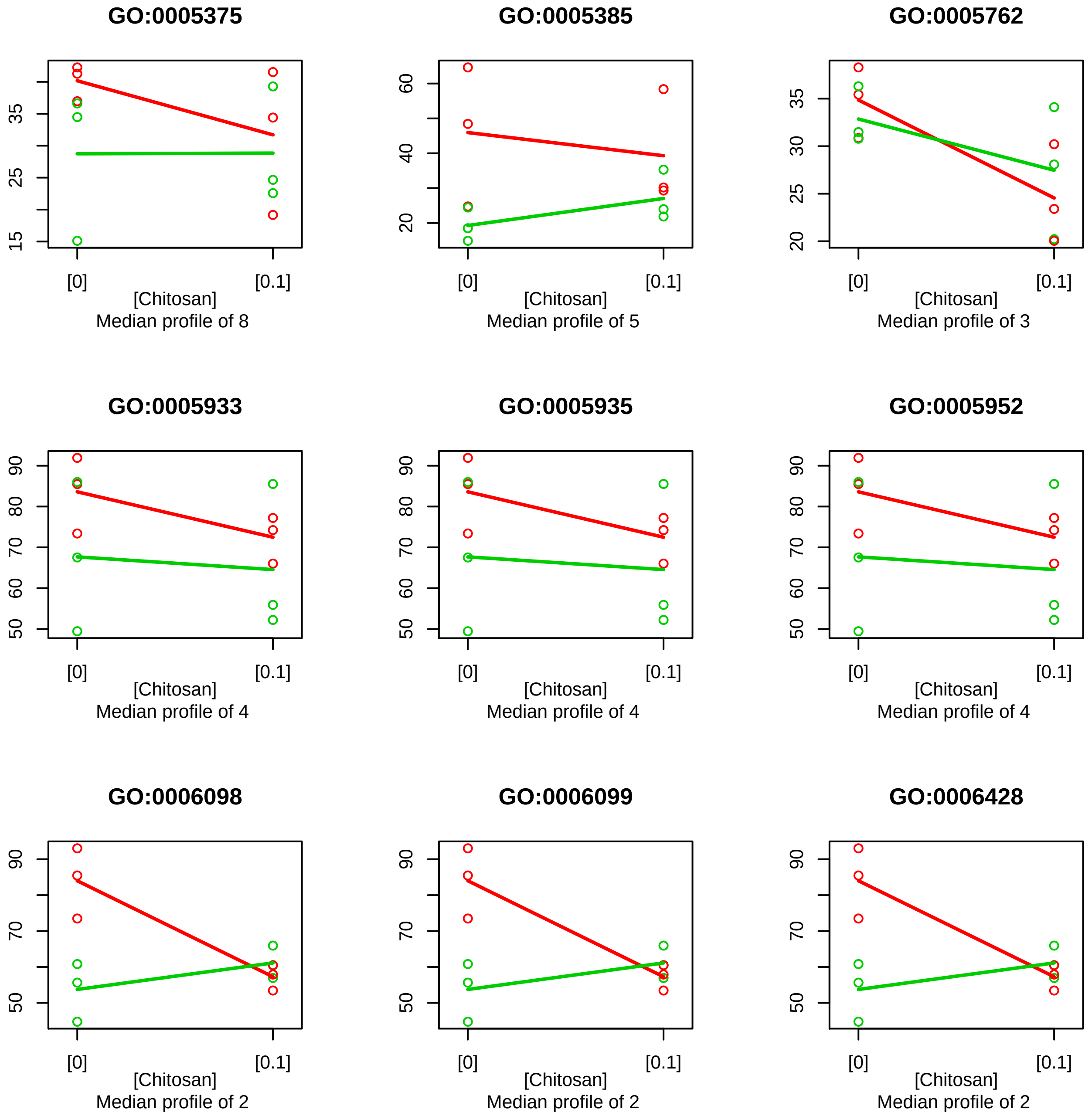
GO:0005375
35
25
15
[0]
[0.1]
[Chitosan]
Median profile of 8
GO:0005385
60
40
20
[0]
[0.1]
[Chitosan]
Median profile of 5
GO:0005762
35
30
25
20
[0]
[0.1]
[Chitosan]
Median profile of 3
GO:0005933
90
80
70
60
50
[0]
[0.1]
[Chitosan]
Median profile of 4
GO:0005935
90
80
70
60
50
[0]
[0.1]
[Chitosan]
Median profile of 4
GO:0005952
90
80
70
60
50
[0]
[0.1]
[Chitosan]
Median profile of 4
GO:0006098
90
70
50
[0]
[0.1]
[Chitosan]
Median profile of 2
GO:0006099
90
70
50
[0]
[0.1]
[Chitosan]
Median profile of 2
GO:0006428
90
70
50
[0]
[0.1]
[Chitosan]
Median profile of 2

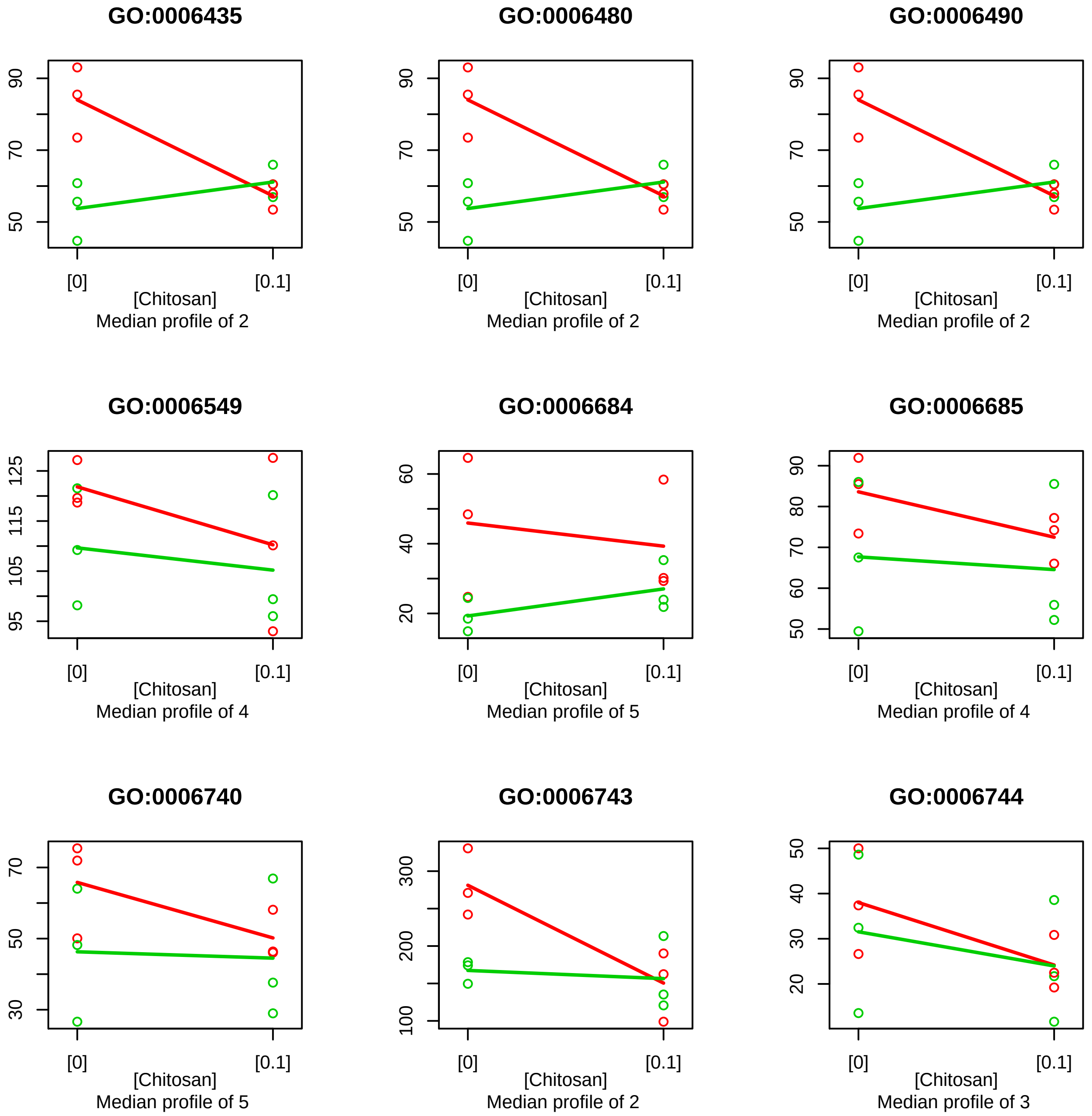
GO:0006435
GO:0006480
GO:0006490
GO:0006549
GO:0006684
GO:0006685
GO:0006740
GO:0006743
GO:0006744
[0]
[0.1]
[Chitosan]
Median profile of 2
Median profile of 4
Median profile of 5
Median profile of 3
50
70
90
95
105
115
125
20
40
60
80
30
100
200
300

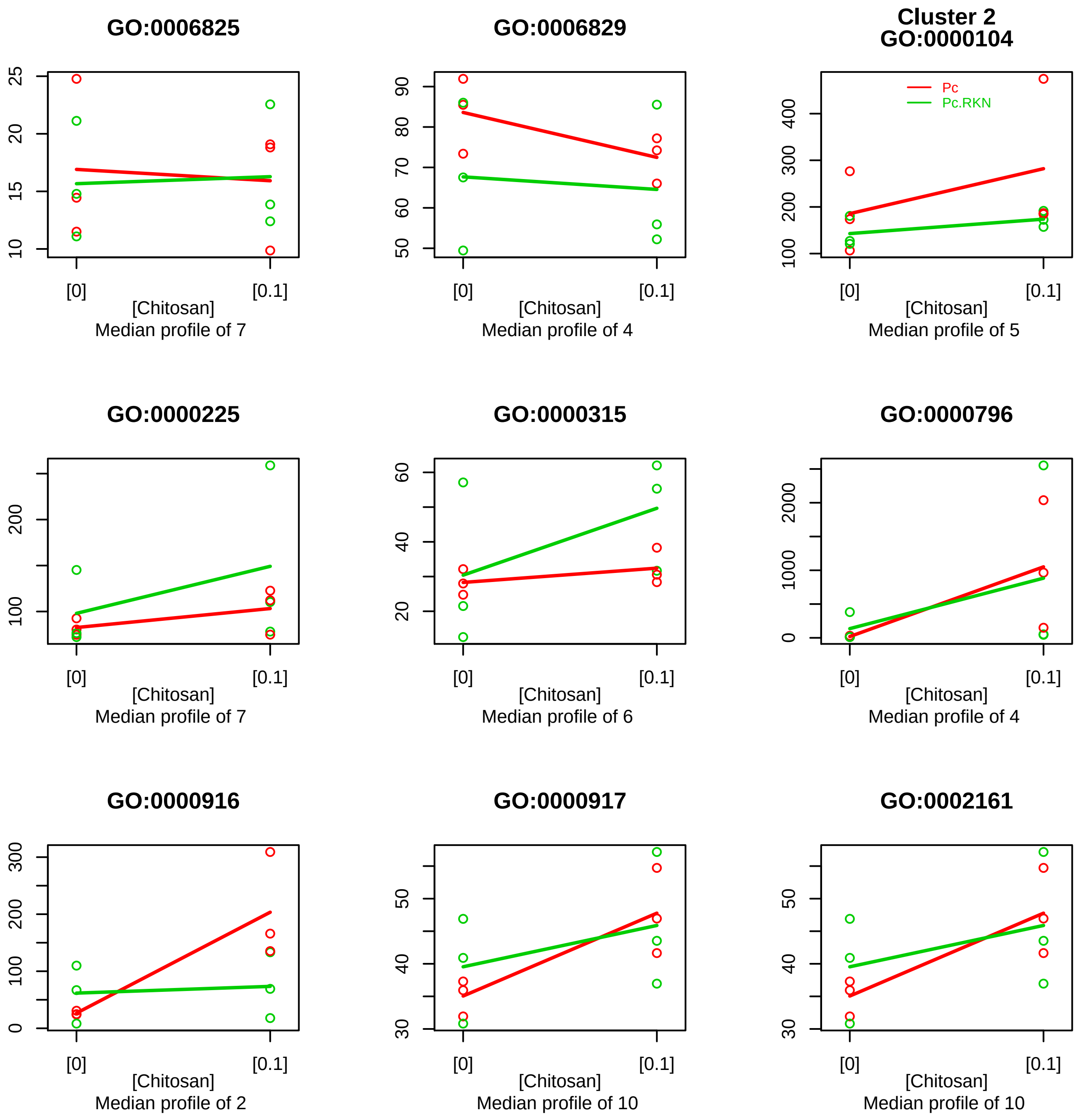

GO:0006825
GO:0006829
Cluster 2
GO:0000104
Pc
Pc.RKN
GO:0000225
GO:0000315
GO:0000796
GO:0000916
GO:0000917
GO:0002161
[0]
[0.1]
[Chitosan]
Median profile of 7
Median profile of 4
Median profile of 5
Median profile of 7
Median profile of 6
Median profile of 4
Median profile of 2
Median profile of 10
Median profile of 10

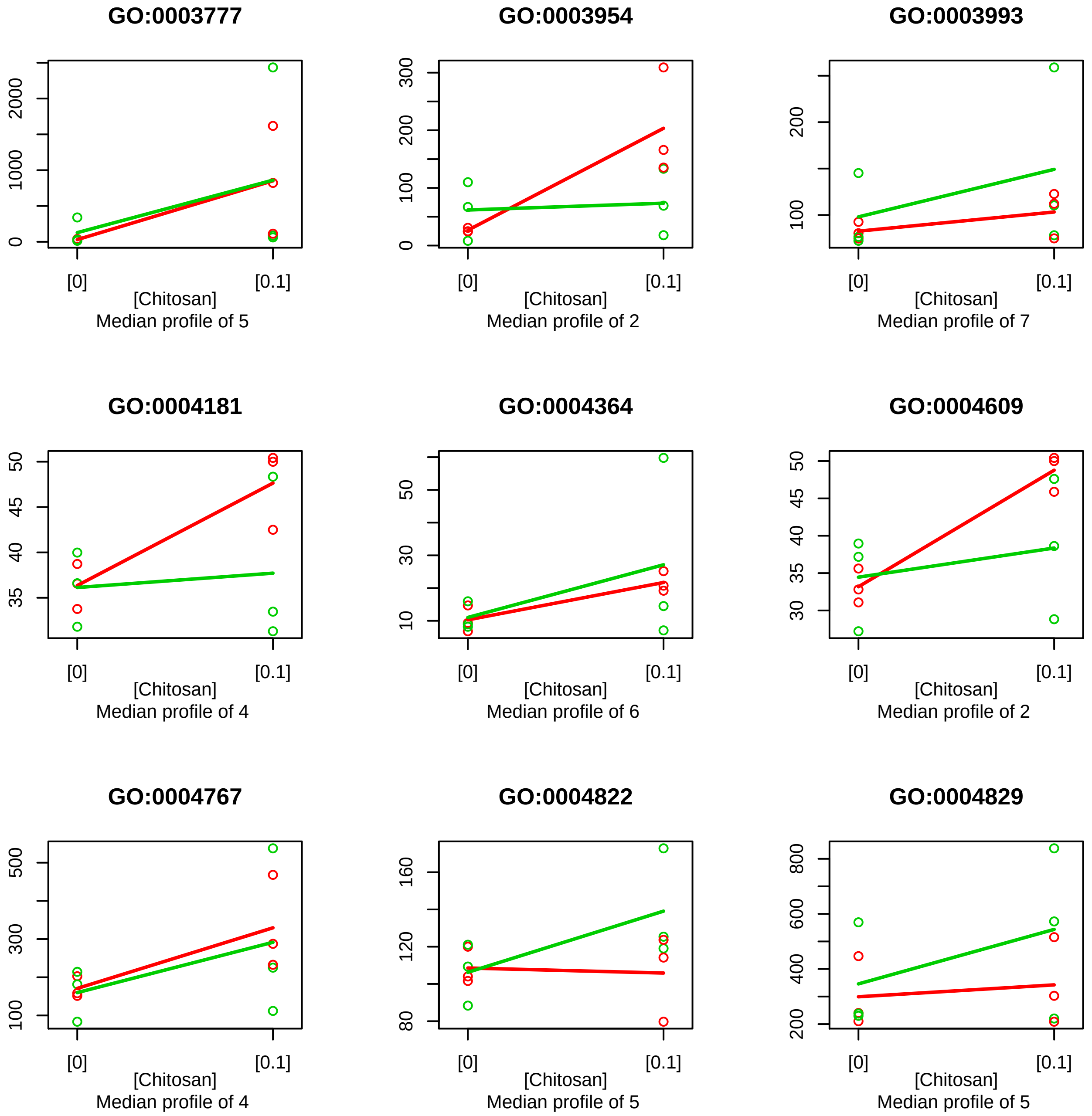
GO:0003777
[0]
[0.1]
[Chitosan]
Median profile of 5
GO:0003954
[0]
[0.1]
[Chitosan]
Median profile of 2
GO:0003993
[0]
[0.1]
[Chitosan]
Median profile of 7
GO:0004181
[0]
[0.1]
[Chitosan]
Median profile of 4
GO:0004364
[0]
[0.1]
[Chitosan]
Median profile of 6
GO:0004609
[0]
[0.1]
[Chitosan]
Median profile of 2
GO:0004767
[0]
[0.1]
[Chitosan]
Median profile of 4
GO:0004822
[0]
[0.1]
[Chitosan]
Median profile of 5
GO:0004829
[0]
[0.1]
[Chitosan]
Median profile of 5

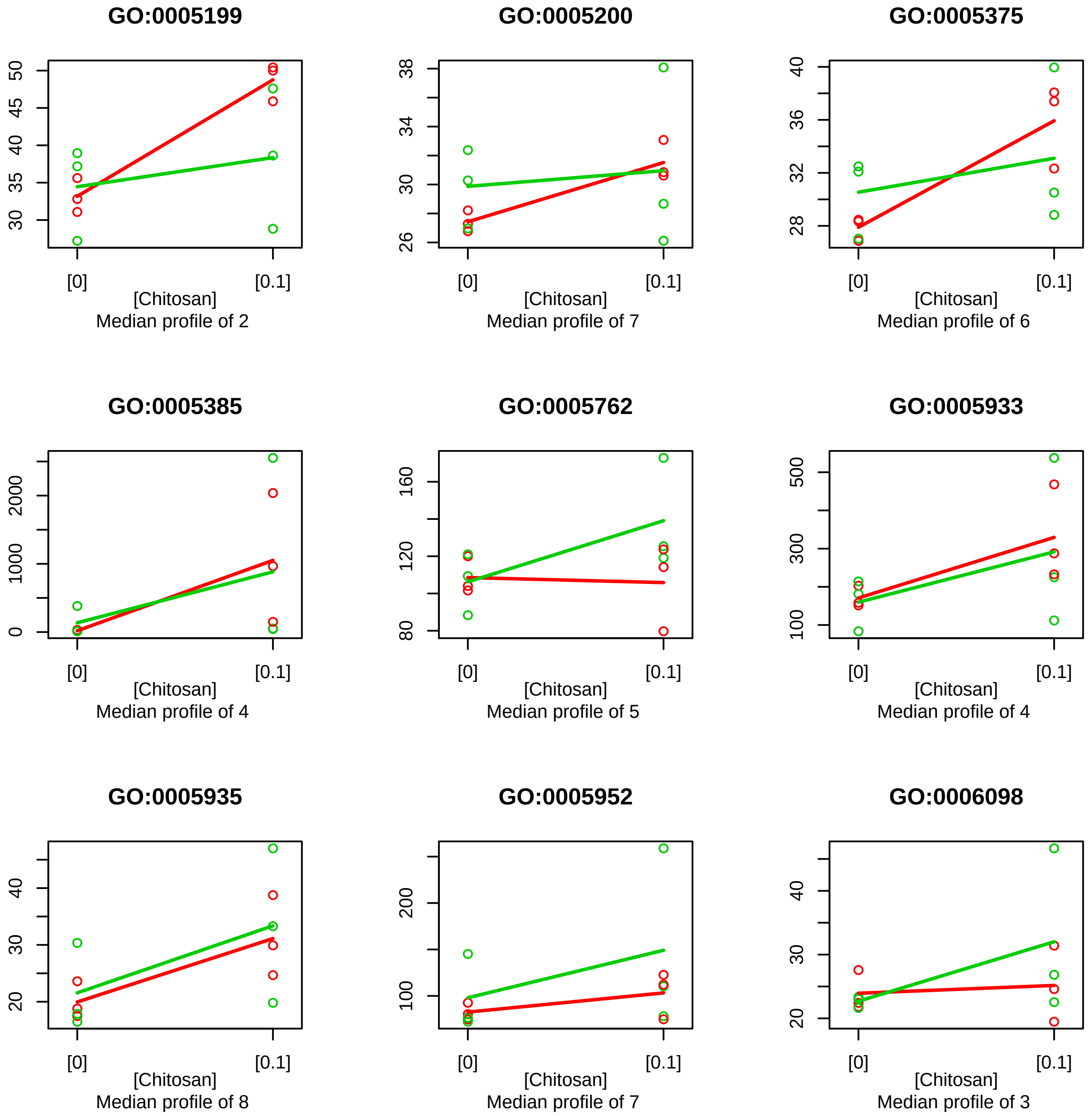
GO:0005199
30 35 40 45 50
[0]
[0.1]
[Chitosan]
Median profile of 2
GO:0005200
26 30 34 38
[0]
[0.1]
[Chitosan]
Median profile of 7
GO:0005375
28 32 36 40
[0]
[0.1]
[Chitosan]
Median profile of 6
GO:0005385
0 1000 2000
[0]
[0.1]
[Chitosan]
Median profile of 4
GO:0005762
80 120 160
[0]
[0.1]
[Chitosan]
Median profile of 5
GO:0005933
100 300 500
[0]
[0.1]
[Chitosan]
Median profile of 4
GO:0005935
20 30 40
[0]
[0.1]
[Chitosan]
Median profile of 8
GO:0005952
100 200
[0]
[0.1]
[Chitosan]
Median profile of 7
GO:0006098
20 30 40
[0]
[0.1]
[Chitosan]
Median profile of 3

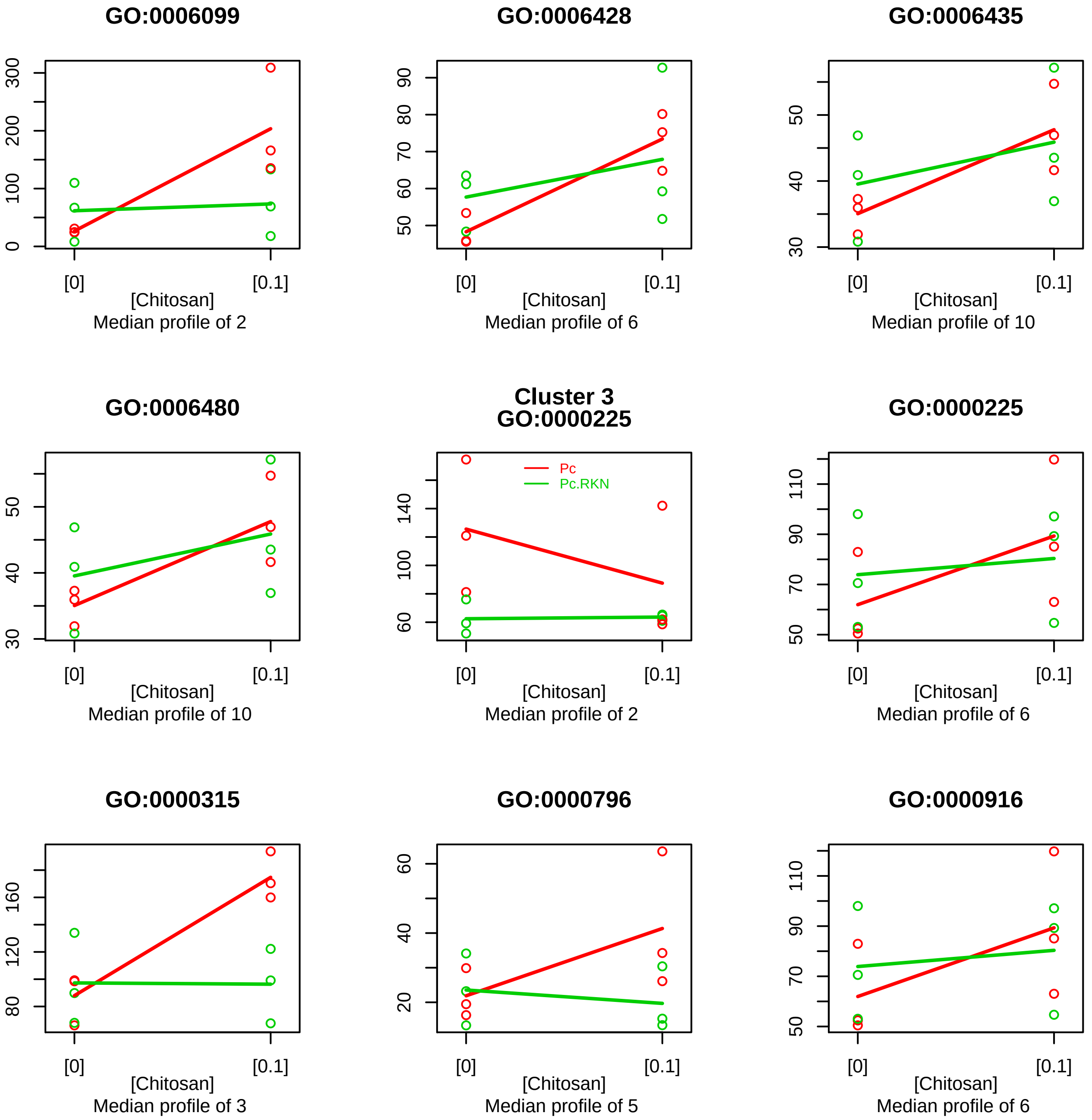
GO:0006099
0
100
200
300
[0]
[0.1]
[Chitosan]
Median profile of 2
GO:0006428
50
60
70
80
90
[0]
[0.1]
[Chitosan]
Median profile of 6
GO:0006435
30
40
50
[0]
[0.1]
[Chitosan]
Median profile of 10
GO:0006480
30
40
50
[0]
[0.1]
[Chitosan]
Median profile of 10
Cluster 3
GO:0000225
Pc
Pc.RKN
60
100
140
[0]
[0.1]
[Chitosan]
Median profile of 2
GO:0000225
50
70
90
110
[0]
[0.1]
[Chitosan]
Median profile of 6
GO:0000315
80
120
160
[0]
[0.1]
[Chitosan]
Median profile of 3
GO:0000796
20
40
60
[0]
[0.1]
[Chitosan]
Median profile of 5
GO:0000916
50
70
90
110
[0]
[0.1]
[Chitosan]
Median profile of 6

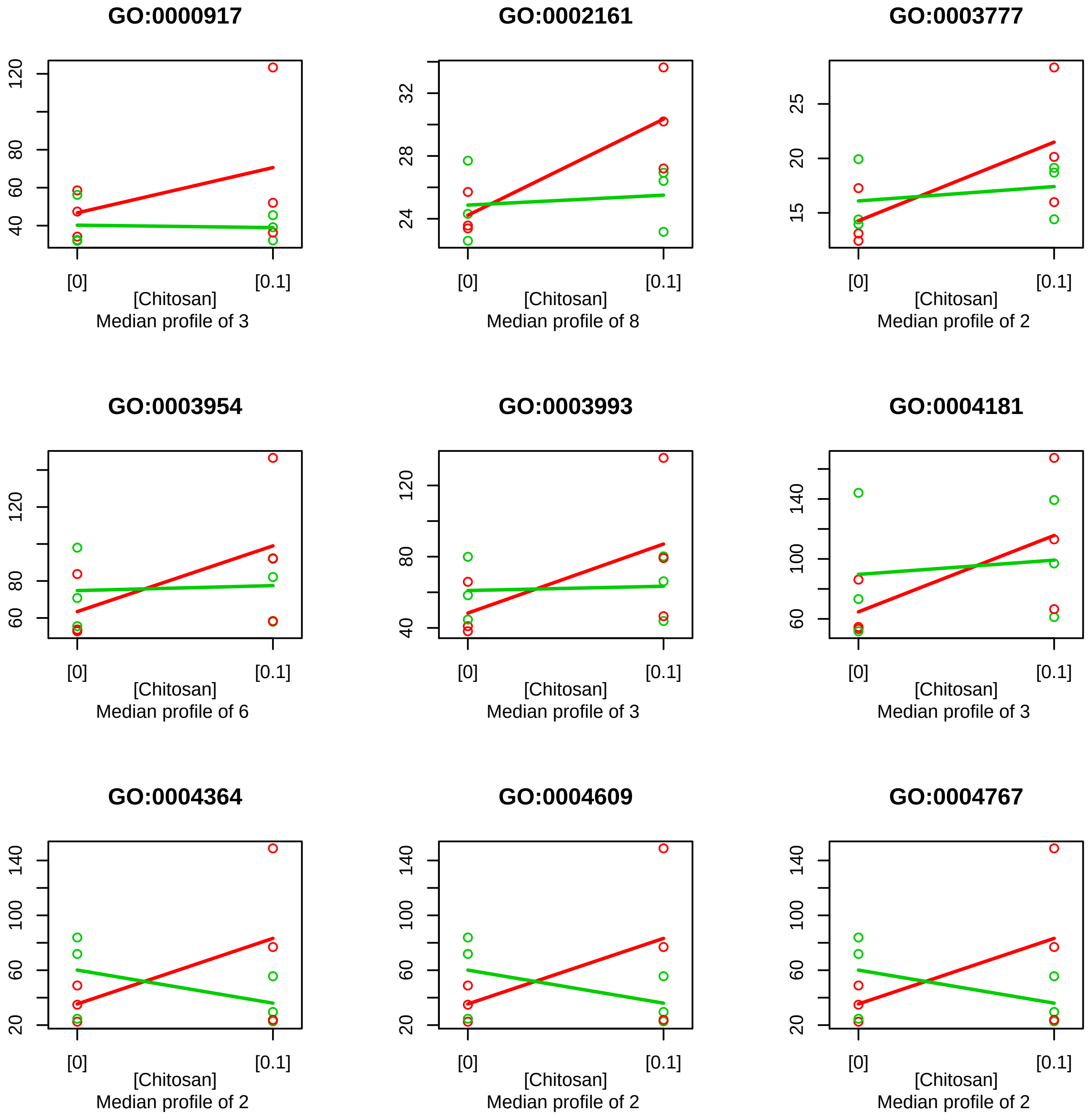
GO:0000917
120
80
60
40
[0]
[0.1]
[Chitosan]
Median profile of 3
GO:0002161
32
28
24
[0]
[0.1]
[Chitosan]
Median profile of 8
GO:0003777
25
20
15
[0]
[0.1]
[Chitosan]
Median profile of 2
GO:0003954
120
80
60
[0]
[0.1]
[Chitosan]
Median profile of 6
GO:0003993
120
80
40
[0]
[0.1]
[Chitosan]
Median profile of 3
GO:0004181
140
100
60
[0]
[0.1]
[Chitosan]
Median profile of 3
GO:0004364
140
100
60
20
[0]
[0.1]
[Chitosan]
Median profile of 2
GO:0004609
140
100
60
20
[0]
[0.1]
[Chitosan]
Median profile of 2
GO:0004767
140
100
60
20
[0]
[0.1]
[Chitosan]
Median profile of 2

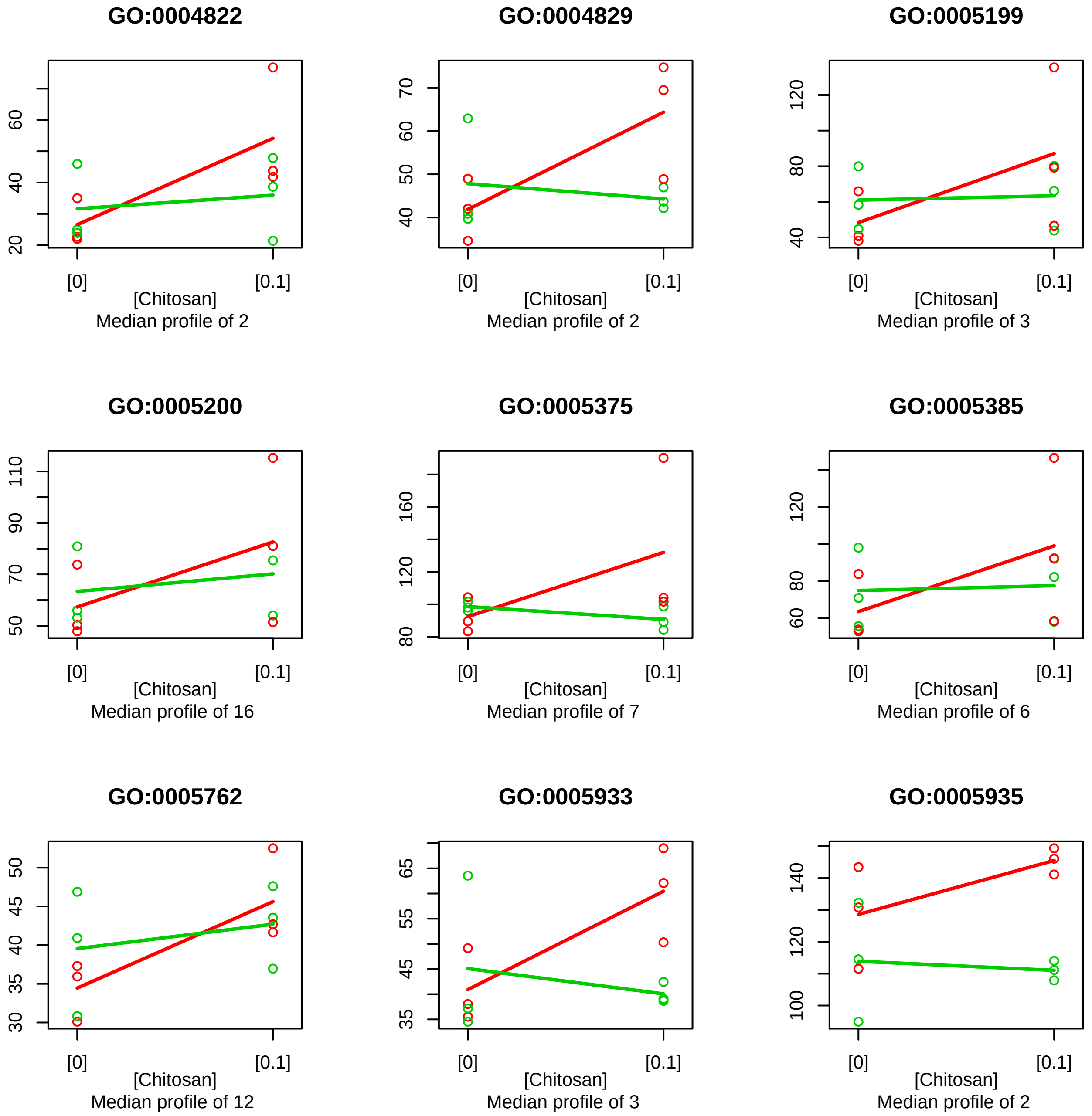
GO:0004822
60
40
20
[0]
[0.1]
[Chitosan]
Median profile of 2
GO:0004829
70
60
50
40
[0]
[0.1]
[Chitosan]
Median profile of 2
GO:0005199
120
80
40
[0]
[0.1]
[Chitosan]
Median profile of 3
GO:0005200
110
90
70
50
[0]
[0.1]
[Chitosan]
Median profile of 16
GO:0005375
160
120
80
[0]
[0.1]
[Chitosan]
Median profile of 7
GO:0005385
120
80
60
[0]
[0.1]
[Chitosan]
Median profile of 6
GO:0005762
50
45
40
35
30
[0]
[0.1]
[Chitosan]
Median profile of 12
GO:0005933
65
55
45
35
[0]
[0.1]
[Chitosan]
Median profile of 3
GO:0005935
140
120
100
[0]
[0.1]
[Chitosan]
Median profile of 2

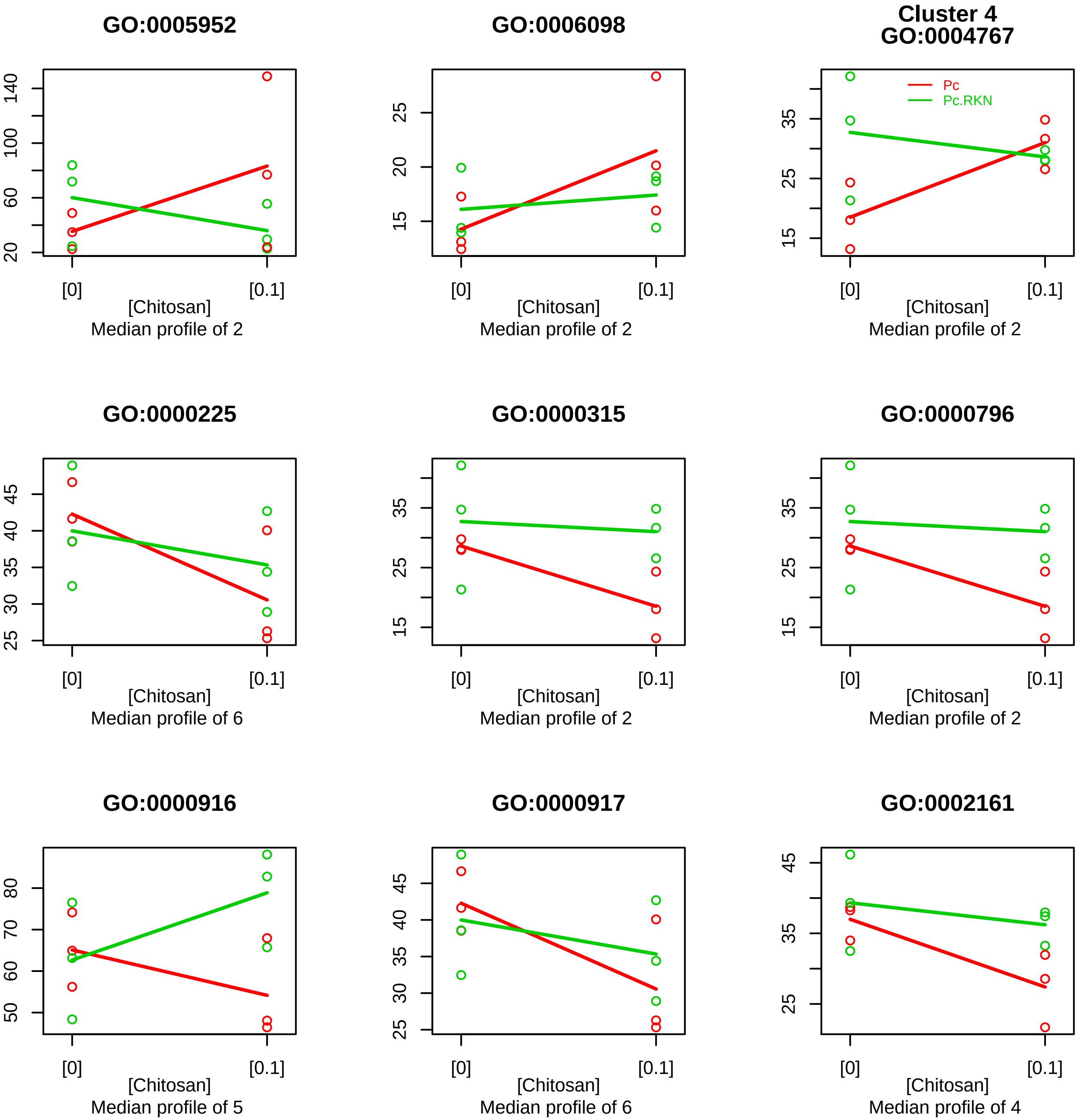
GO:0005952
GO:0006098
Cluster 4
GO:0004767
Pc
Pc.RKN
GO:0000225
GO:0000315
GO:0000796
GO:0000916
GO:0000917
GO:0002161
[0]
[0.1]
[Chitosan]
Median profile of 2
Median profile of 6
Median profile of 5
Median profile of 4

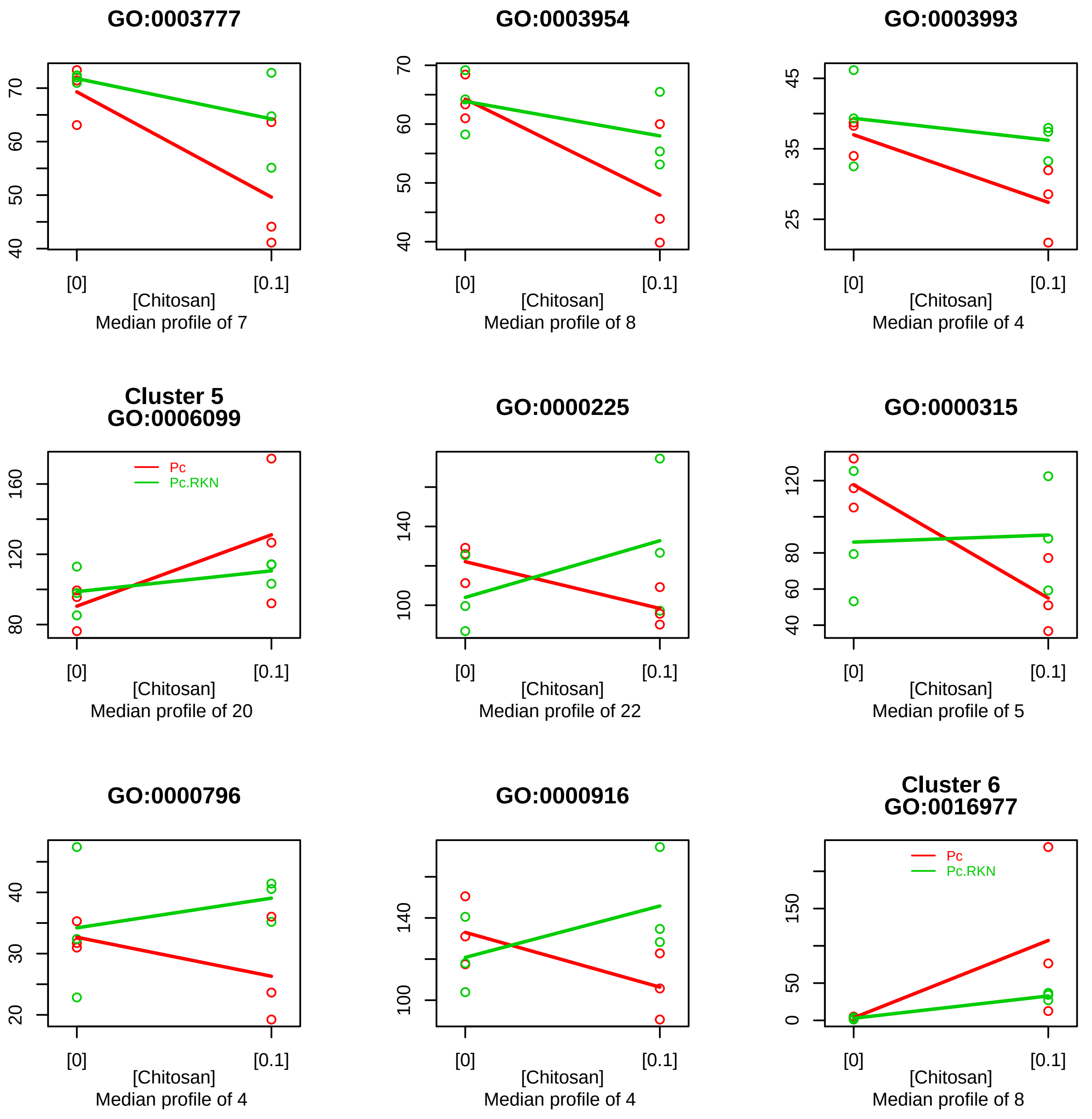

GO:0003777
GO:0003954
GO:0003993
Cluster 5
GO:0006099
GO:0000225
GO:0000315
GO:0000796
GO:0000916
Cluster 6
GO:0016977
Pc
Pc.RKN
[0]
[0.1]
[Chitosan]
Median profile of 7
Median profile of 8
Median profile of 4
Median profile of 20
Median profile of 22
Median profile of 5
Median profile of 4
Median profile of 4
Median profile of 8

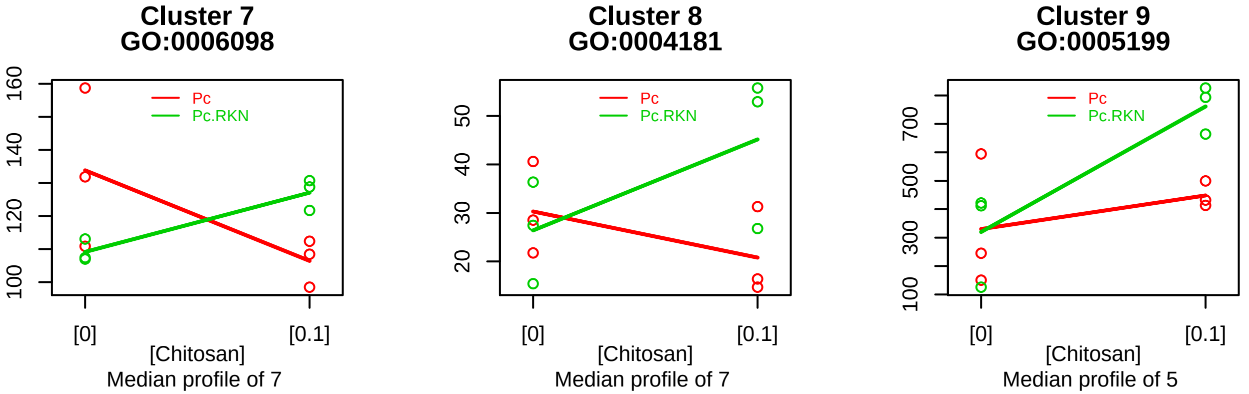
Cluster 7
GO:0006098
160
140
120
100
Pc
Pc.RKN
[0]
[0.1]
[Chitosan]
Median profile of 7
Cluster 8
GO:0004181
50
40
30
20
Pc
Pc.RKN
[0]
[0.1]
[Chitosan]
Median profile of 7
Cluster 9
GO:0005199
700
500
300
100
Pc
Pc.RKN
[0]
[0.1]
[Chitosan]
Median profile of 5
