# Supplementary material for: Chitosan modulates Pochonia chlamydosporia gene expression during nematode egg parasitism
Source: Environ Microbiol. 2021 Feb 5;23(9):4980–97. doi: 10.1111/1462-2920.15408 (PMC8518118; doi:10.1111/1462-2920.15408)
Supplement: Supplementary file 4 — Supplementary Fig. 3. Individual trends of 180 genes included in clusters in Fig. 4. [file EMI-23-4980-s006.pdf]

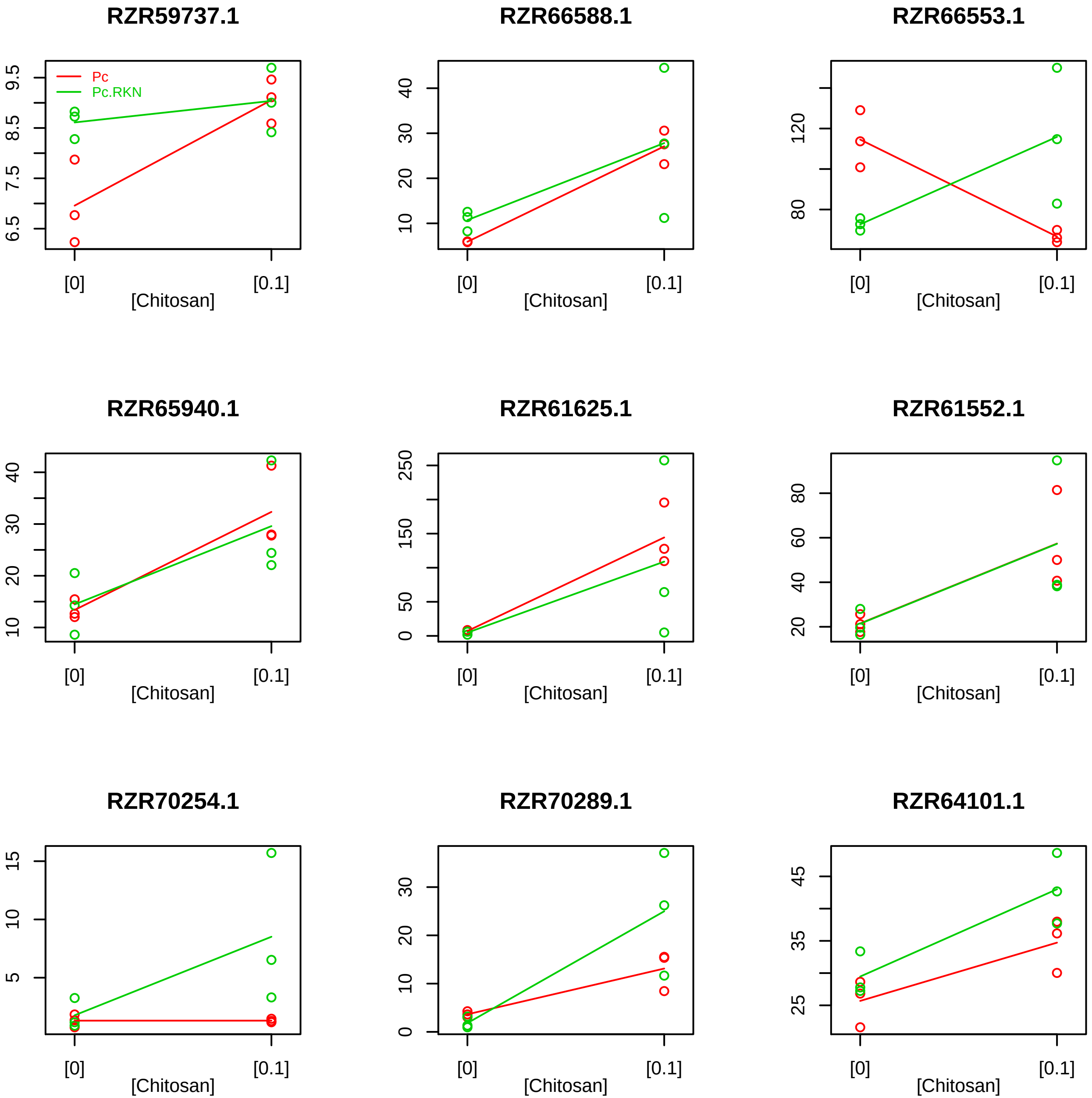
RZR59737.1
RZR66588.1
RZR66553.1
RZR65940.1
RZR61625.1
RZR61552.1
RZR70254.1
RZR70289.1
RZR64101.1
Pc
Pc.RKN
[0]
[0.1]
[Chitosan]

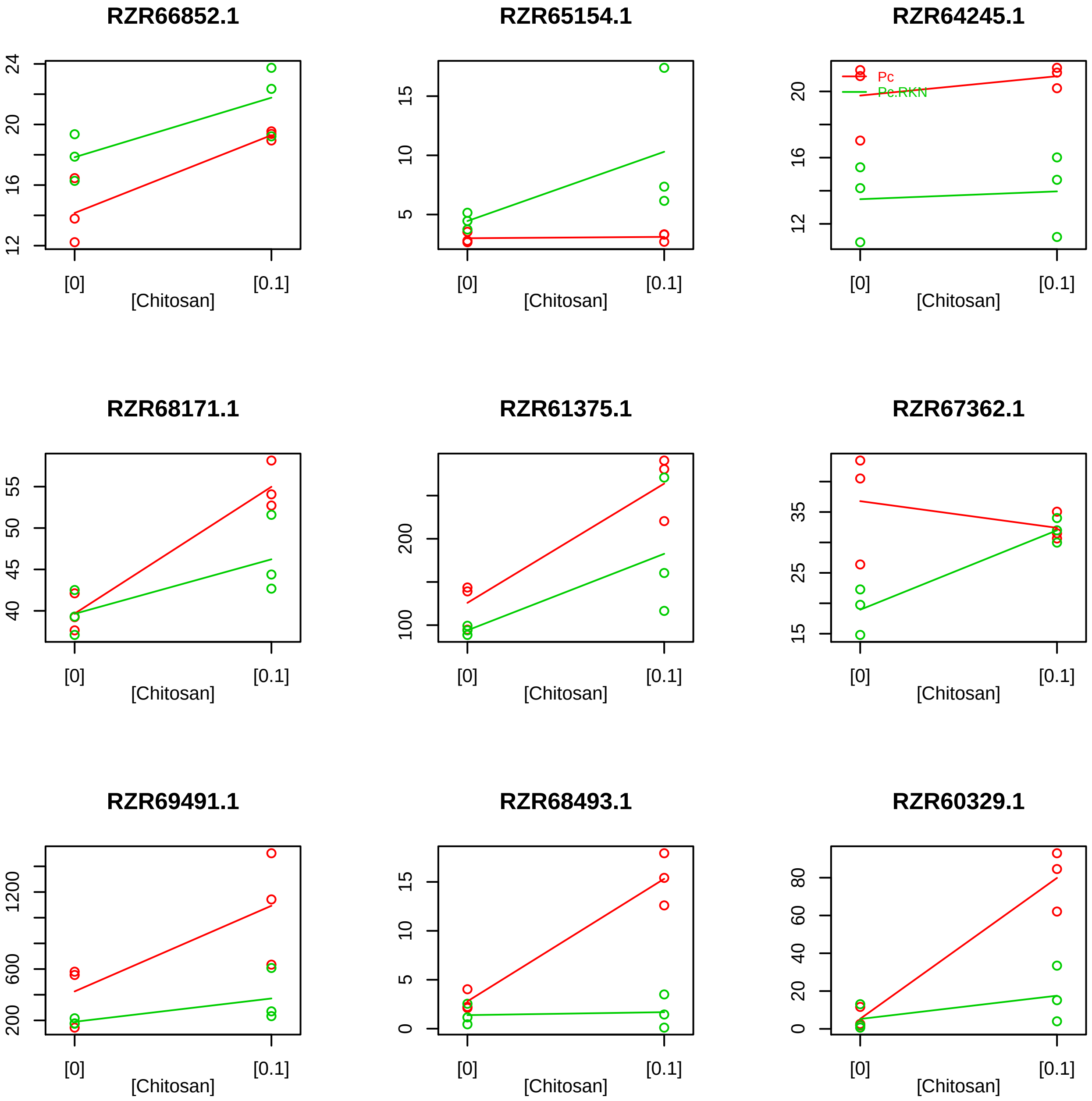
RZR66852.1
RZR65154.1
RZR64245.1
Pc
Pc.RKN
RZR68171.1
RZR61375.1
RZR67362.1
RZR69491.1
RZR68493.1
RZR60329.1
[0]
[0.1]
[Chitosan]

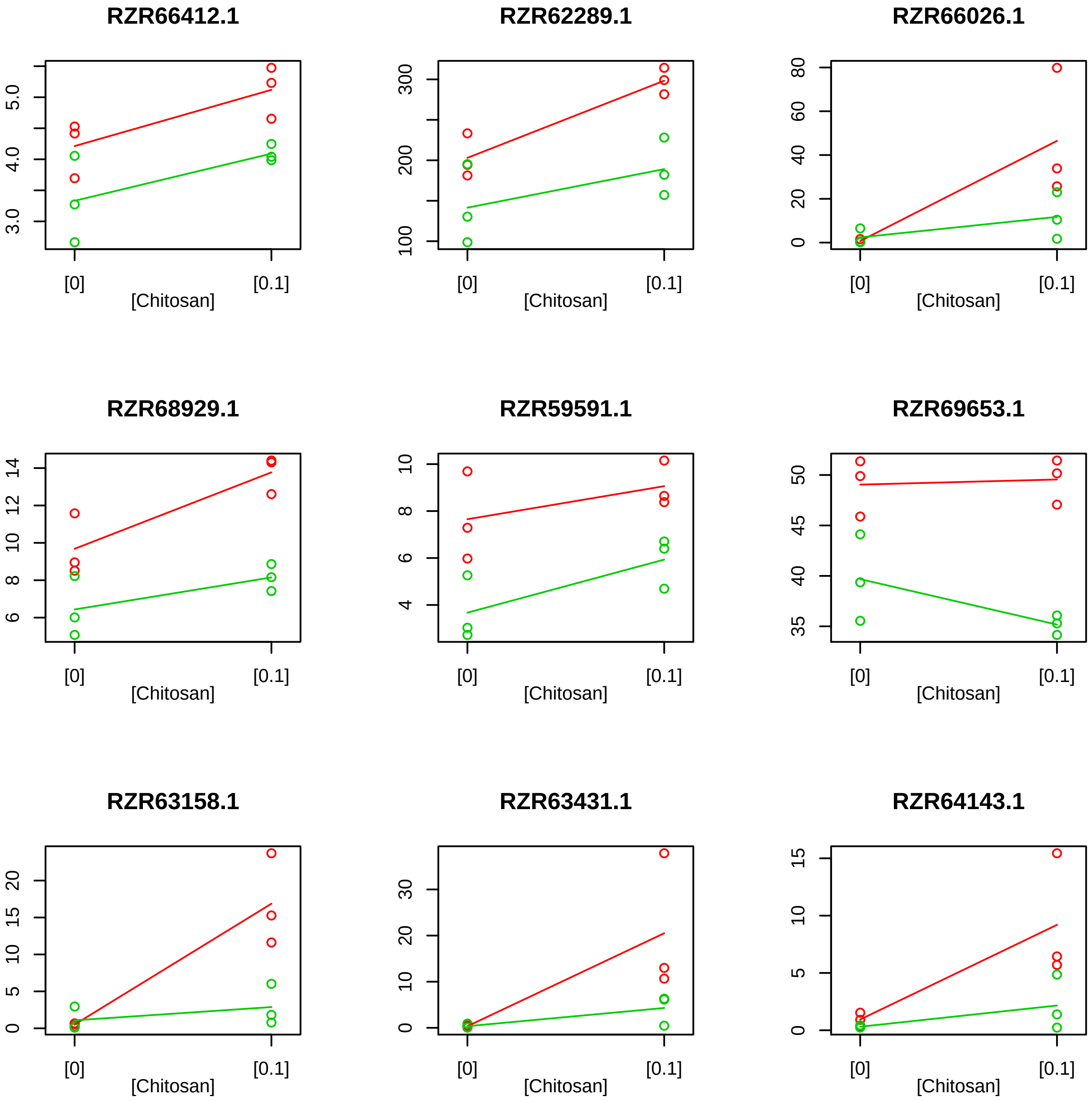
RZR66412.1
3.0
4.0
5.0
[0]
[0.1]
[Chitosan]
RZR62289.1
100
200
300
[0]
[0.1]
[Chitosan]
RZR66026.1
0
20
40
60
80
[0]
[0.1]
[Chitosan]
RZR68929.1
6
8
10
12
14
[0]
[0.1]
[Chitosan]
RZR59591.1
4
6
8
10
[0]
[0.1]
[Chitosan]
RZR69653.1
35
40
45
50
[0]
[0.1]
[Chitosan]
RZR63158.1
0
5
10
15
20
[0]
[0.1]
[Chitosan]
RZR63431.1
0
10
20
30
[0]
[0.1]
[Chitosan]
RZR64143.1
0
5
10
15
[0]
[0.1]
[Chitosan]

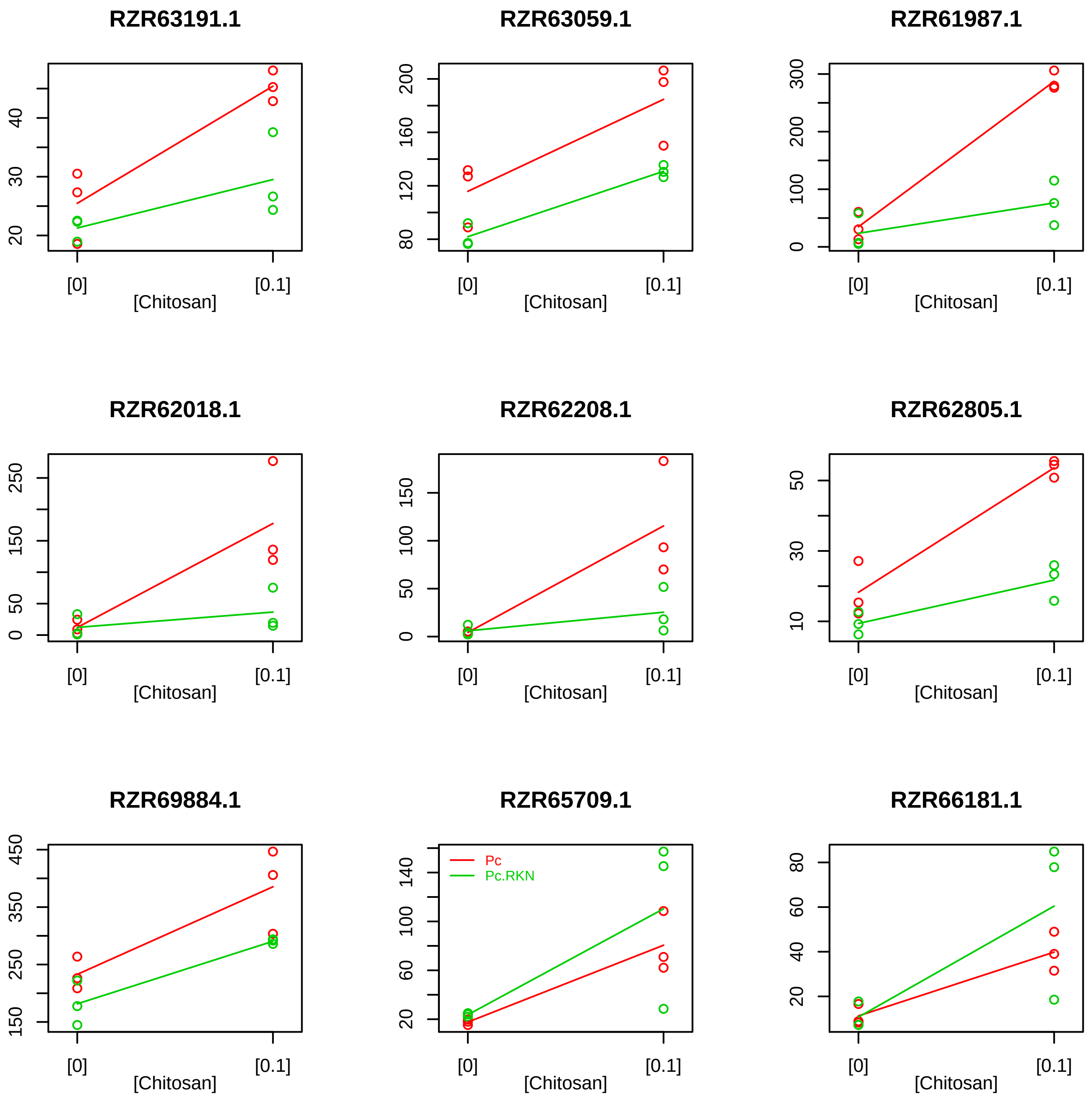
RZR63191.1
RZR63059.1
RZR61987.1
RZR62018.1
RZR62208.1
RZR62805.1
RZR69884.1
RZR65709.1
RZR66181.1
[0]
[0.1]
[Chitosan]
Pc
Pc.RKN

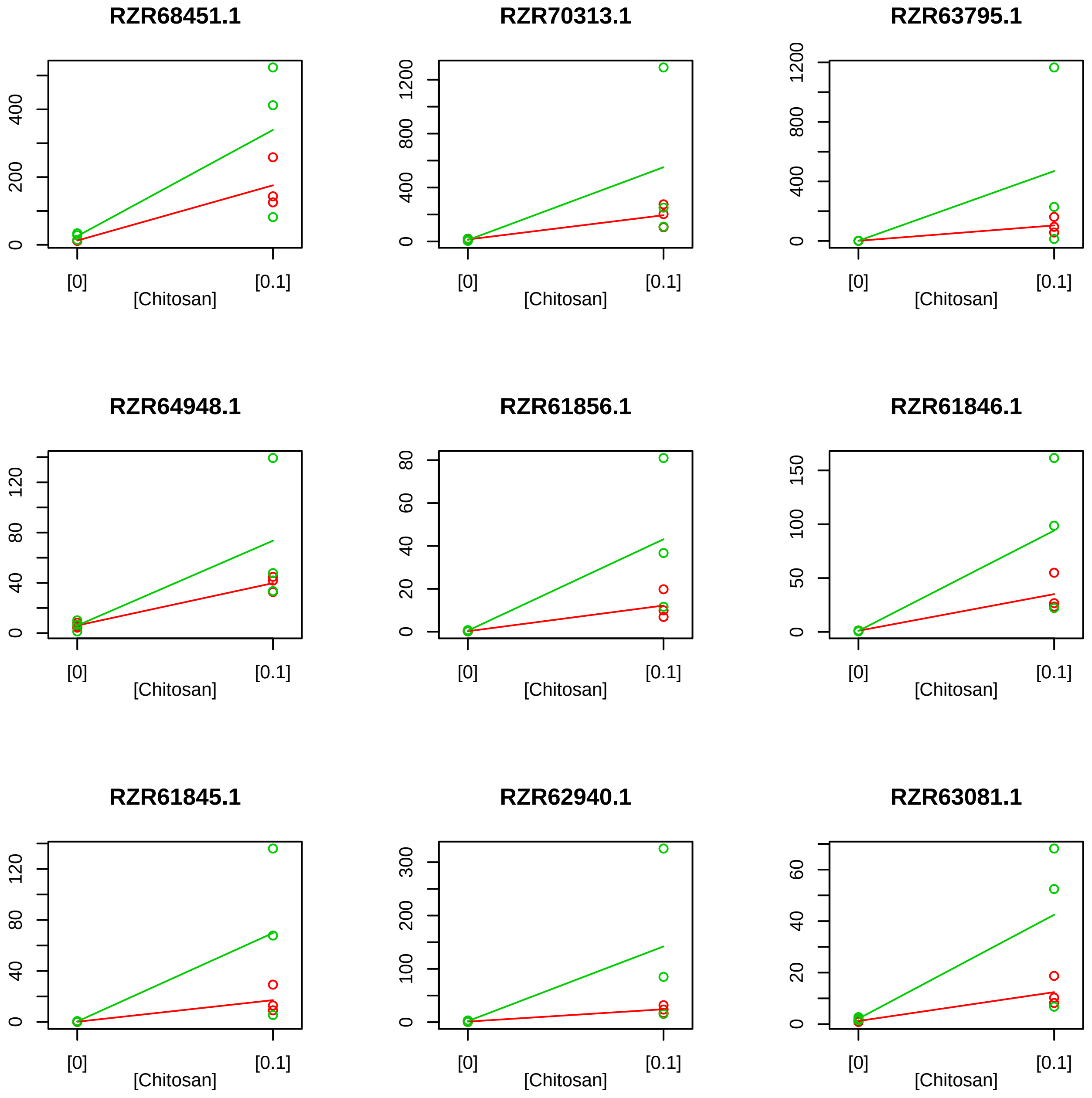
RZR68451.1
RZR70313.1
RZR63795.1
RZR64948.1
RZR61856.1
RZR61846.1
RZR61845.1
RZR62940.1
RZR63081.1
[0]
[0.1]
[Chitosan]

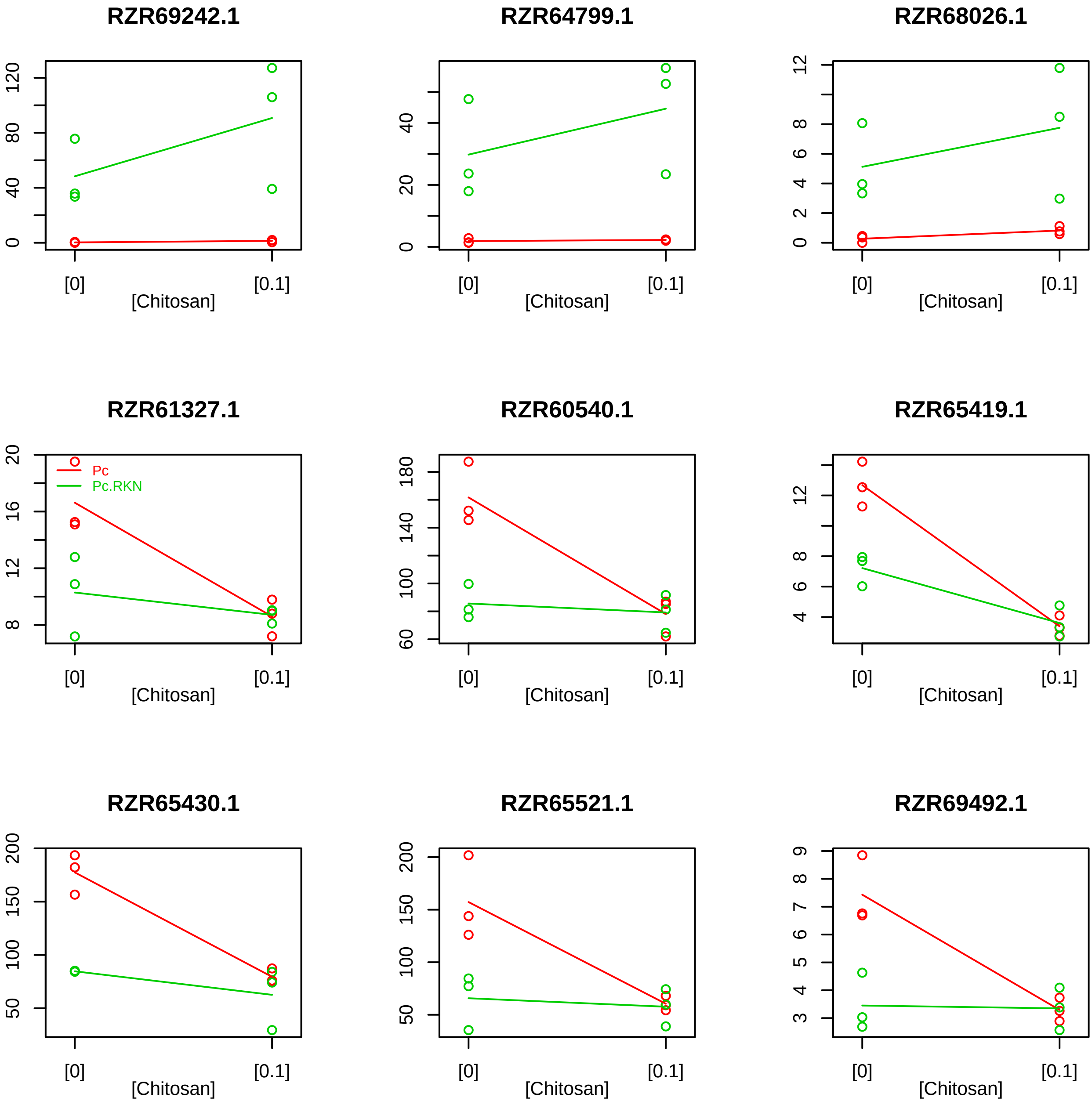
RZR69242.1
0
40
80
120
[0]
[0.1]
[Chitosan]
RZR64799.1
0
20
40
[0]
[0.1]
[Chitosan]
RZR68026.1
0
2
4
6
8
12
[0]
[0.1]
[Chitosan]
RZR61327.1
Pc
Pc.RKN
8
12
16
20
[0]
[0.1]
[Chitosan]
RZR60540.1
60
100
140
180
[0]
[0.1]
[Chitosan]
RZR65419.1
4
6
8
12
[0]
[0.1]
[Chitosan]
RZR65430.1
50
100
150
200
[0]
[0.1]
[Chitosan]
RZR65521.1
50
100
150
200
[0]
[0.1]
[Chitosan]
RZR69492.1
3
4
5
6
7
8
9
[0]
[0.1]
[Chitosan]

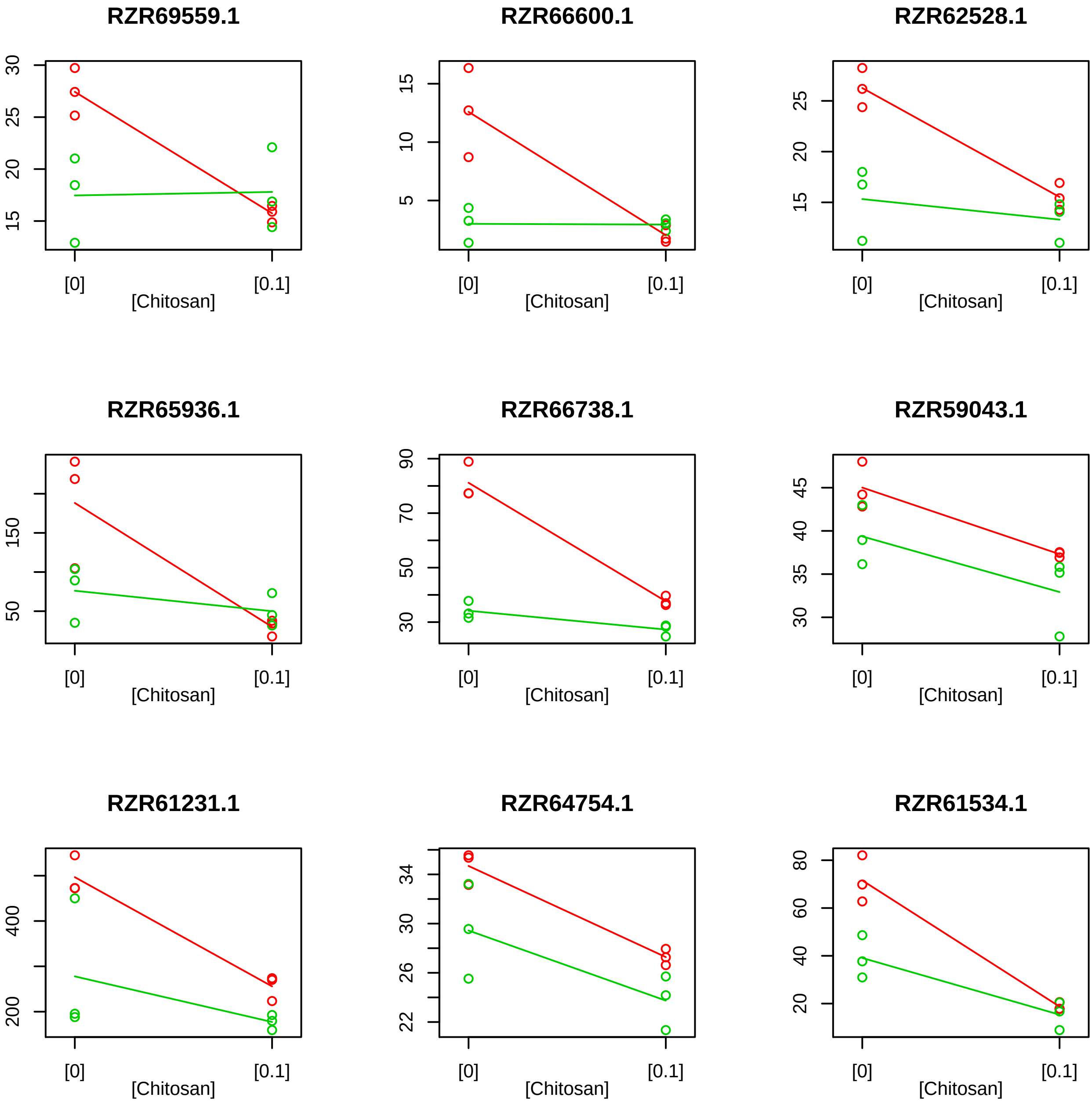
RZR69559.1
30
25
20
15
[0]
[0.1]
[Chitosan]
RZR66600.1
15
10
5
[0]
[0.1]
[Chitosan]
RZR62528.1
25
20
15
[0]
[0.1]
[Chitosan]
RZR65936.1
150
50
[0]
[0.1]
[Chitosan]
RZR66738.1
90
70
50
30
[0]
[0.1]
[Chitosan]
RZR59043.1
45
40
35
30
[0]
[0.1]
[Chitosan]
RZR61231.1
400
200
[0]
[0.1]
[Chitosan]
RZR64754.1
34
30
26
22
[0]
[0.1]
[Chitosan]
RZR61534.1
80
60
40
20
[0]
[0.1]
[Chitosan]

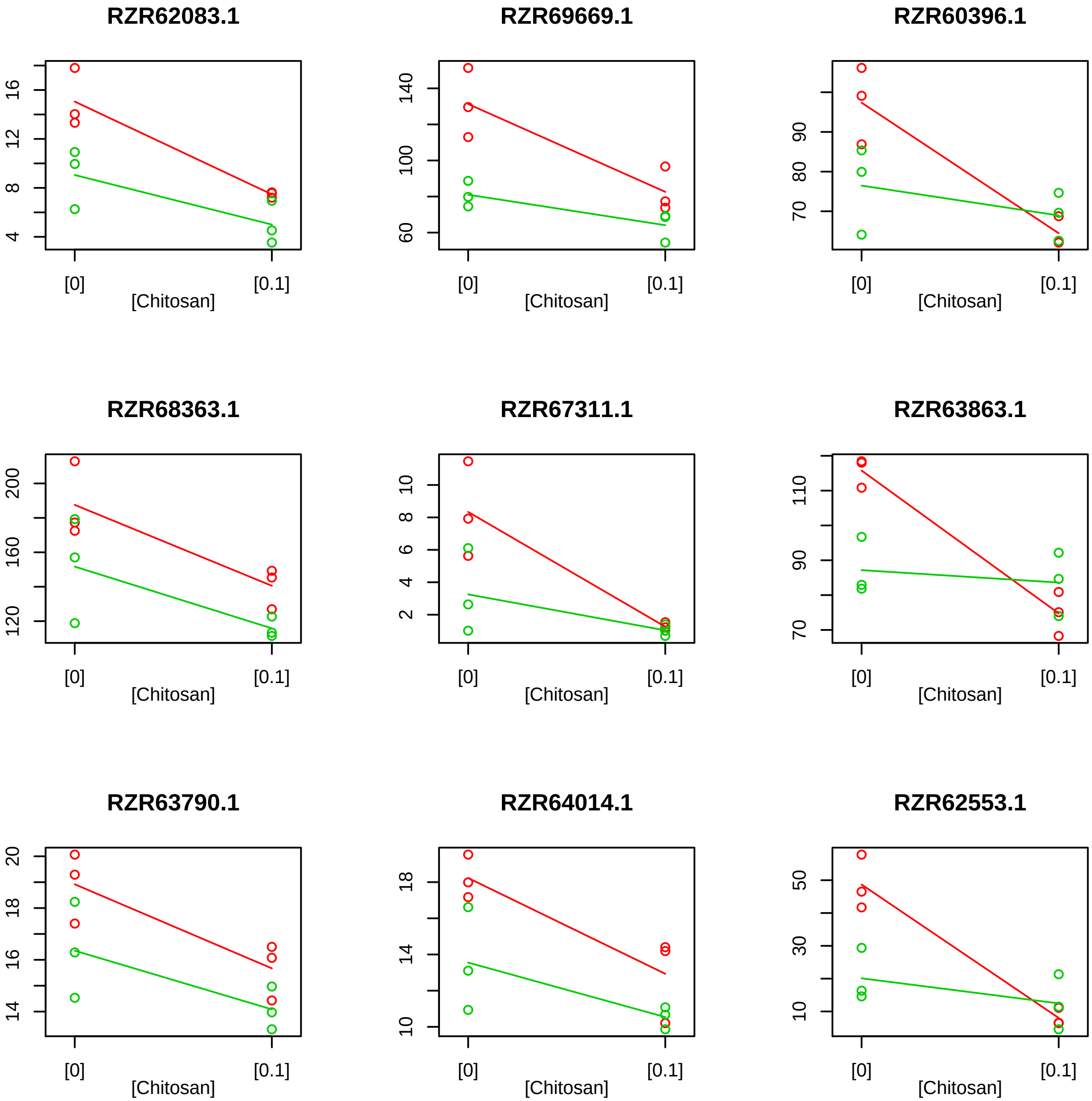
RZR62083.1
16
12
8
4
[0]
[0.1]
[Chitosan]
RZR69669.1
140
100
60
[0]
[0.1]
[Chitosan]
RZR60396.1
90
80
70
[0]
[0.1]
[Chitosan]
RZR68363.1
200
160
120
[0]
[0.1]
[Chitosan]
RZR67311.1
10
8
6
4
2
[0]
[0.1]
[Chitosan]
RZR63863.1
110
90
70
[0]
[0.1]
[Chitosan]
RZR63790.1
20
18
16
14
[0]
[0.1]
[Chitosan]
RZR64014.1
18
14
10
[0]
[0.1]
[Chitosan]
RZR62553.1
50
30
10
[0]
[0.1]
[Chitosan]

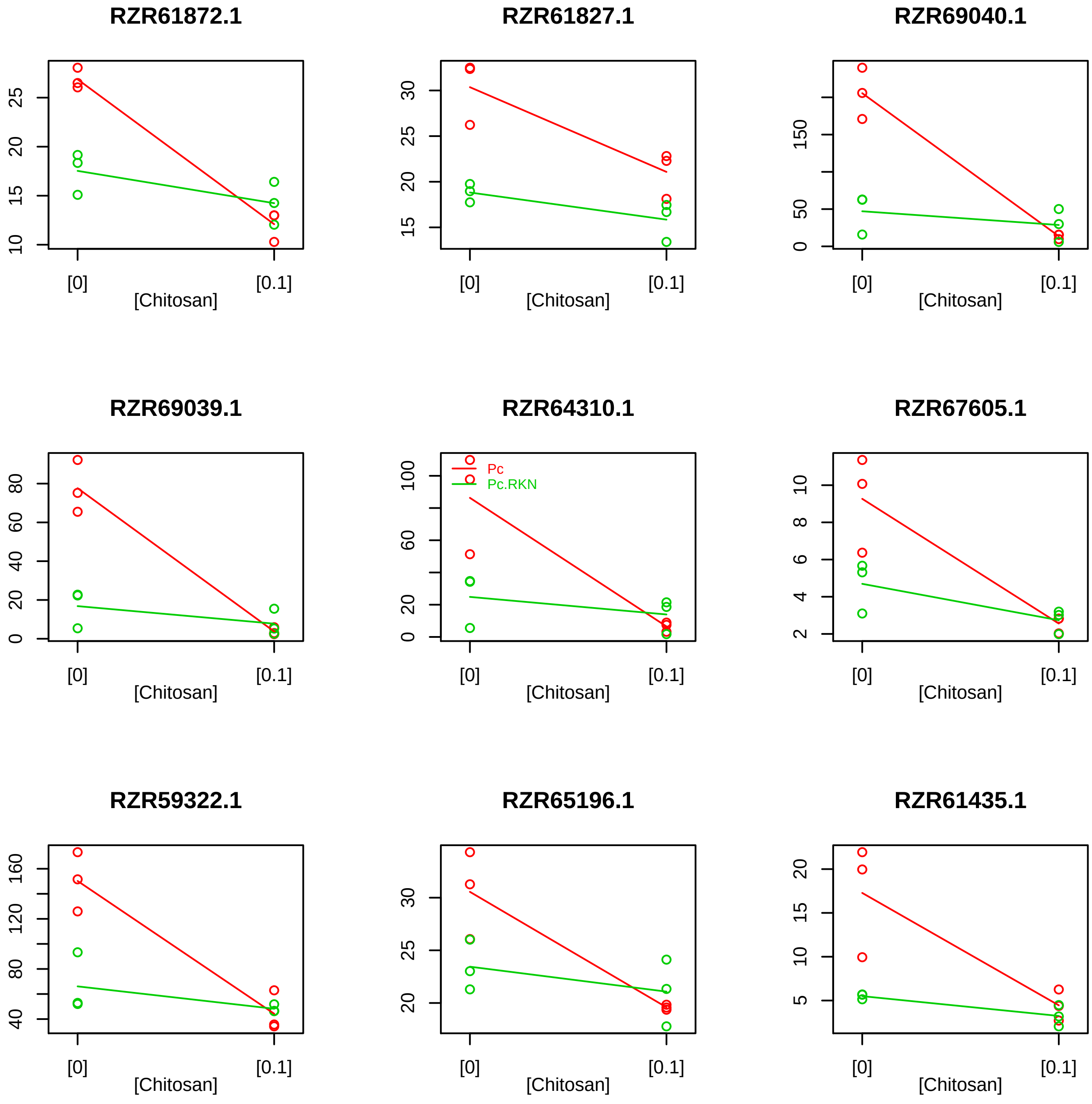
RZR61872.1
RZR61827.1
RZR69040.1
RZR69039.1
RZR64310.1
RZR67605.1
RZR59322.1
RZR65196.1
RZR61435.1
Pc
Pc.RKN
[0]
[0.1]
[Chitosan]

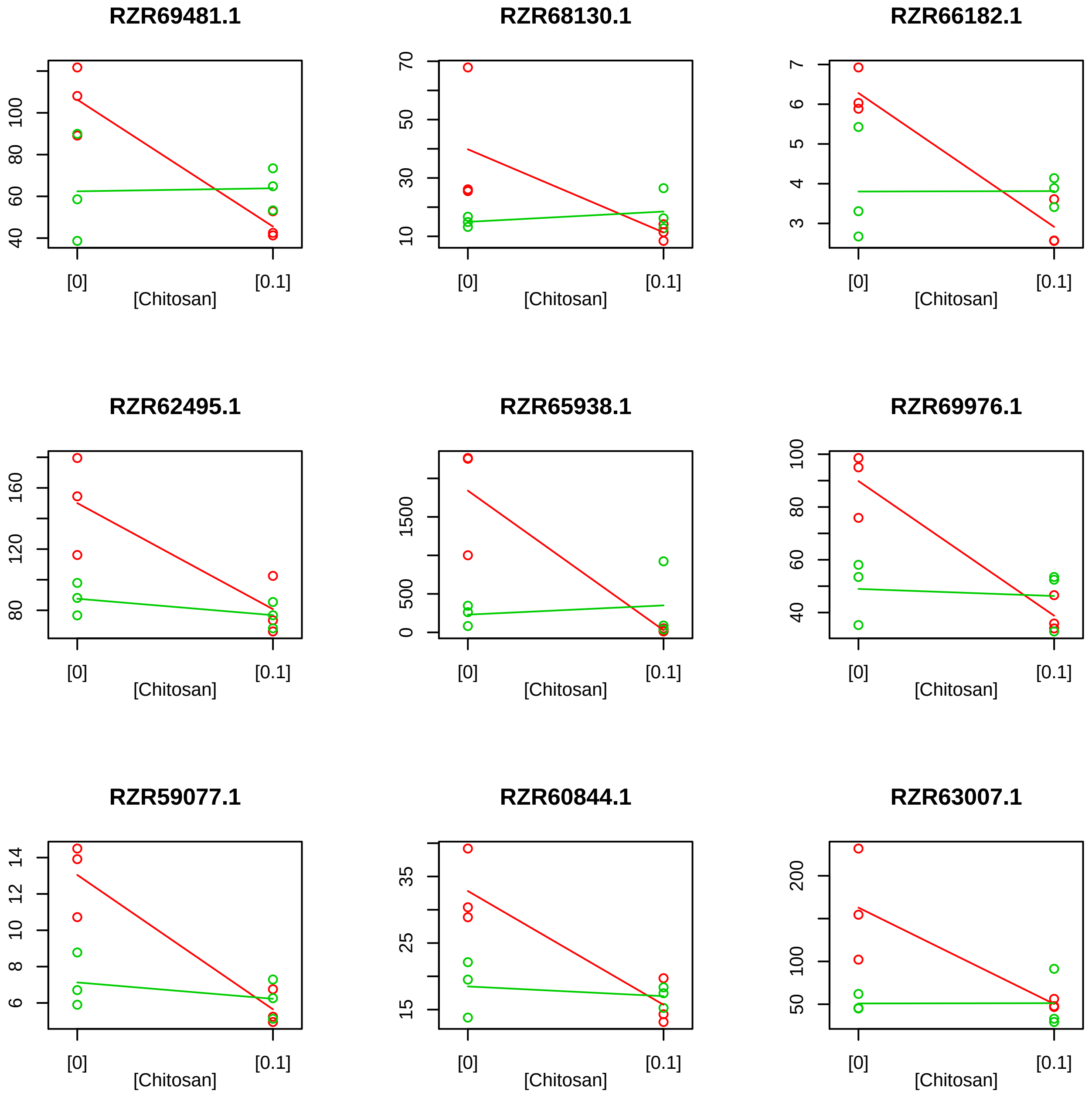
RZR69481.1
40
60
80
100
[0]
[0.1]
[Chitosan]
RZR68130.1
10
30
50
70
[0]
[0.1]
[Chitosan]
RZR66182.1
3
4
5
6
7
[0]
[0.1]
[Chitosan]
RZR62495.1
80
120
160
[0]
[0.1]
[Chitosan]
RZR65938.1
0
500
1500
[0]
[0.1]
[Chitosan]
RZR69976.1
40
60
80
100
[0]
[0.1]
[Chitosan]
RZR59077.1
6
8
10
12
14
[0]
[0.1]
[Chitosan]
RZR60844.1
15
25
35
[0]
[0.1]
[Chitosan]
RZR63007.1
50
100
200
[0]
[0.1]
[Chitosan]

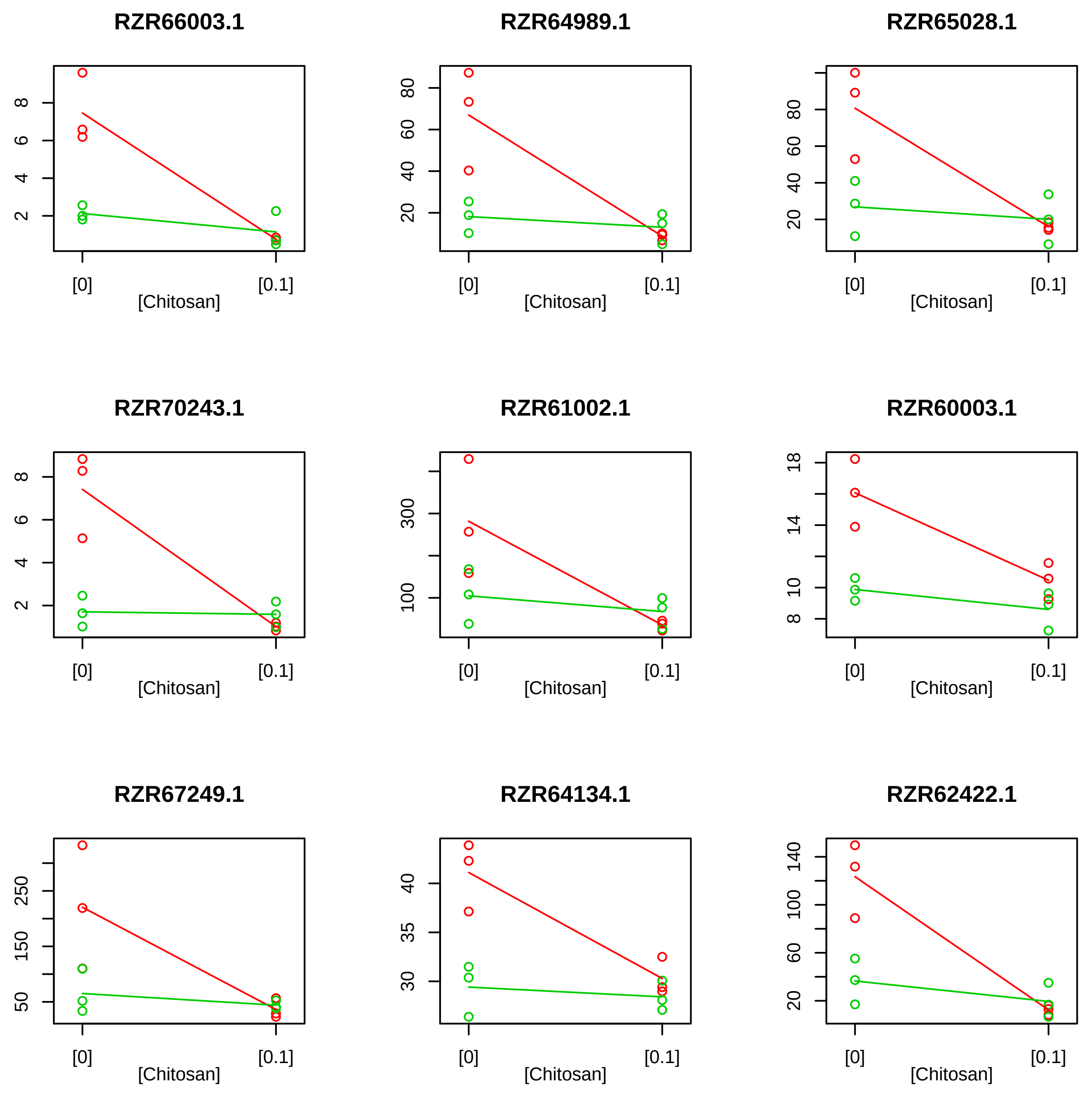
RZR66003.1
2
4
6
8
[0]
[0.1]
[Chitosan]
RZR64989.1
20
40
60
80
[0]
[0.1]
[Chitosan]
RZR65028.1
20
40
60
80
[0]
[0.1]
[Chitosan]
RZR70243.1
2
4
6
8
[0]
[0.1]
[Chitosan]
RZR61002.1
100
300
[0]
[0.1]
[Chitosan]
RZR60003.1
8
10
14
18
[0]
[0.1]
[Chitosan]
RZR67249.1
50
150
250
[0]
[0.1]
[Chitosan]
RZR64134.1
30
35
40
[0]
[0.1]
[Chitosan]
RZR62422.1
20
60
100
140
[0]
[0.1]
[Chitosan]

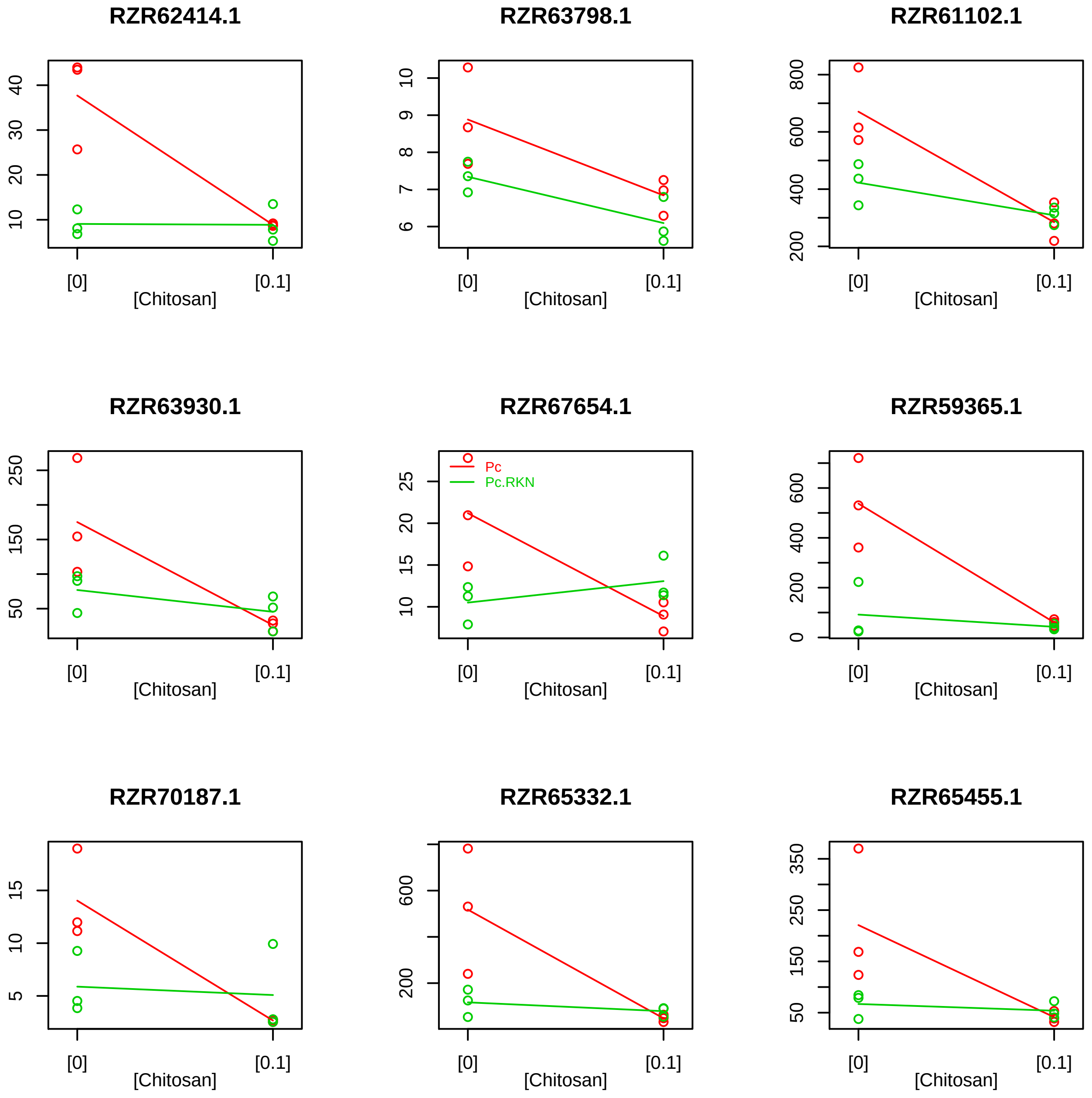
RZR62414.1
RZR63798.1
RZR61102.1
RZR63930.1
RZR67654.1
RZR59365.1
RZR70187.1
RZR65332.1
RZR65455.1
Pc
Pc.RKN
[0]
[0.1]
[Chitosan]

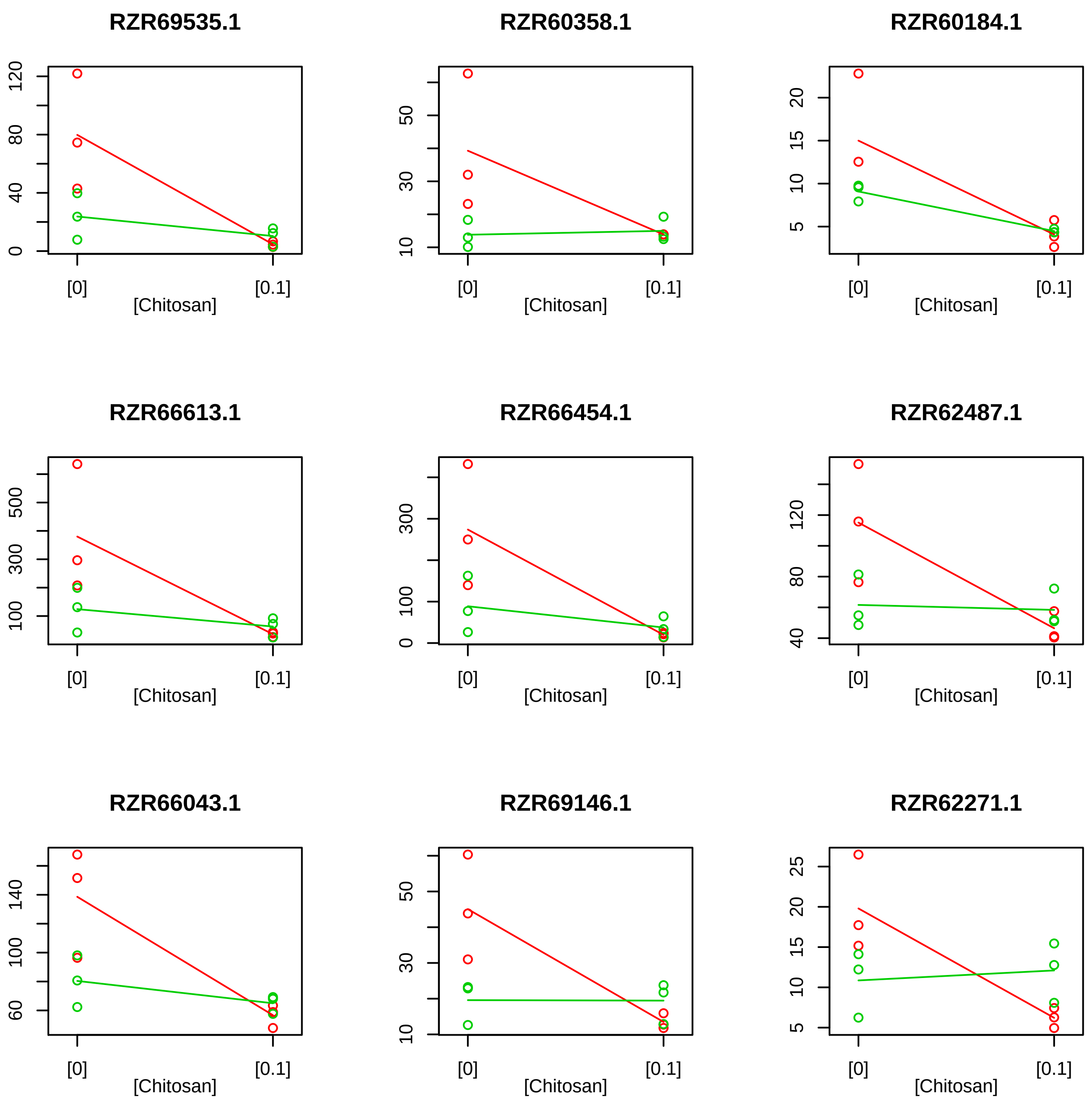
RZR69535.1
0
40
80
120
[0]
[0.1]
[Chitosan]
RZR60358.1
10
30
50
[0]
[0.1]
[Chitosan]
RZR60184.1
5
10
15
20
[0]
[0.1]
[Chitosan]
RZR66613.1
100
300
500
[0]
[0.1]
[Chitosan]
RZR66454.1
0
100
300
[0]
[0.1]
[Chitosan]
RZR62487.1
40
80
120
[0]
[0.1]
[Chitosan]
RZR66043.1
60
100
140
[0]
[0.1]
[Chitosan]
RZR69146.1
10
30
50
[0]
[0.1]
[Chitosan]
RZR62271.1
5
10
15
20
25
[0]
[0.1]
[Chitosan]

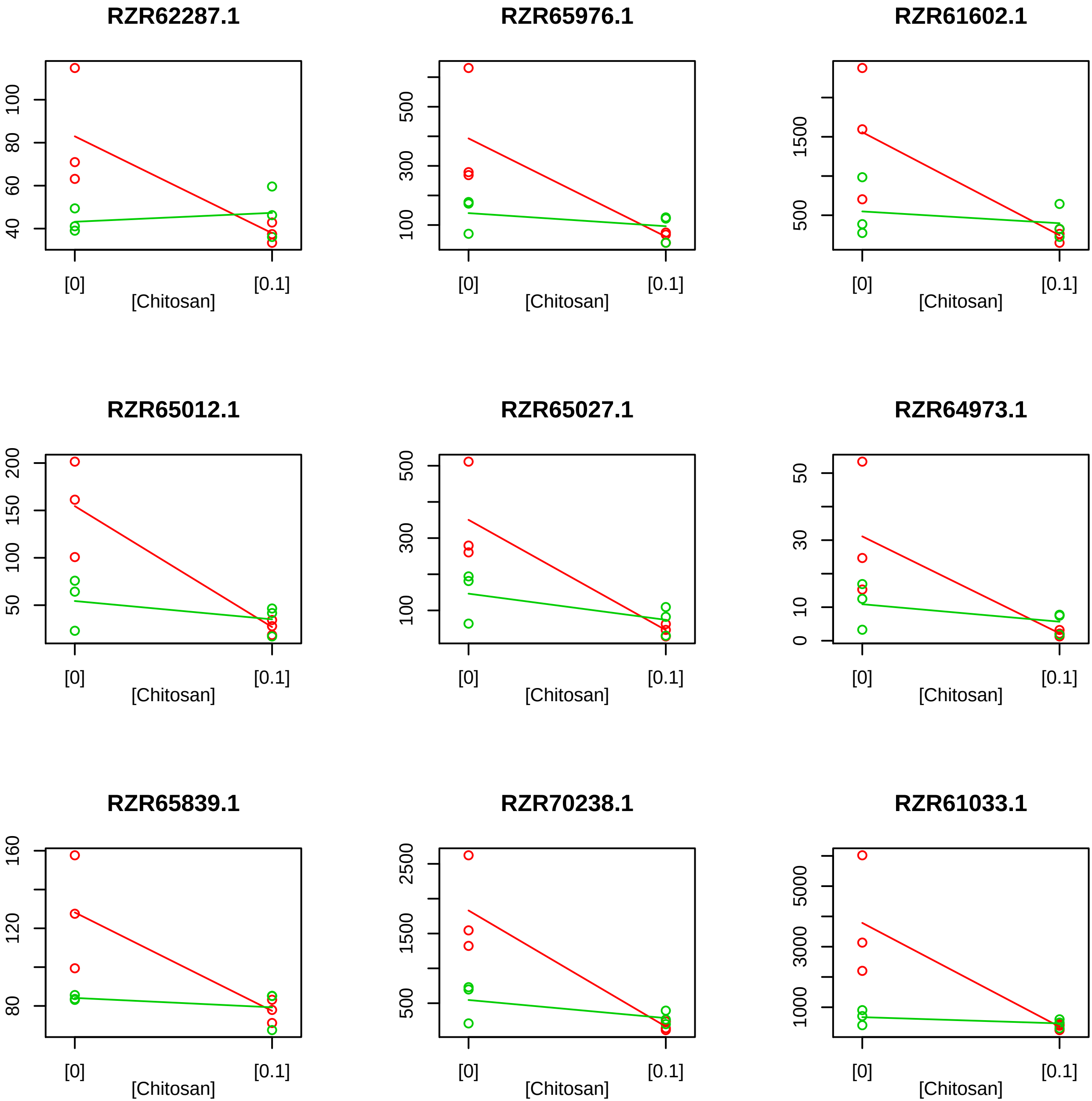
RZR62287.1
40
60
80
100
[0]
[0.1]
[Chitosan]
RZR65976.1
100
300
500
[0]
[0.1]
[Chitosan]
RZR61602.1
500
1500
[0]
[0.1]
[Chitosan]
RZR65012.1
50
100
150
200
[0]
[0.1]
[Chitosan]
RZR65027.1
100
300
500
[0]
[0.1]
[Chitosan]
RZR64973.1
0
10
30
50
[0]
[0.1]
[Chitosan]
RZR65839.1
80
120
160
[0]
[0.1]
[Chitosan]
RZR70238.1
500
1500
2500
[0]
[0.1]
[Chitosan]
RZR61033.1
1000
3000
5000
[0]
[0.1]
[Chitosan]

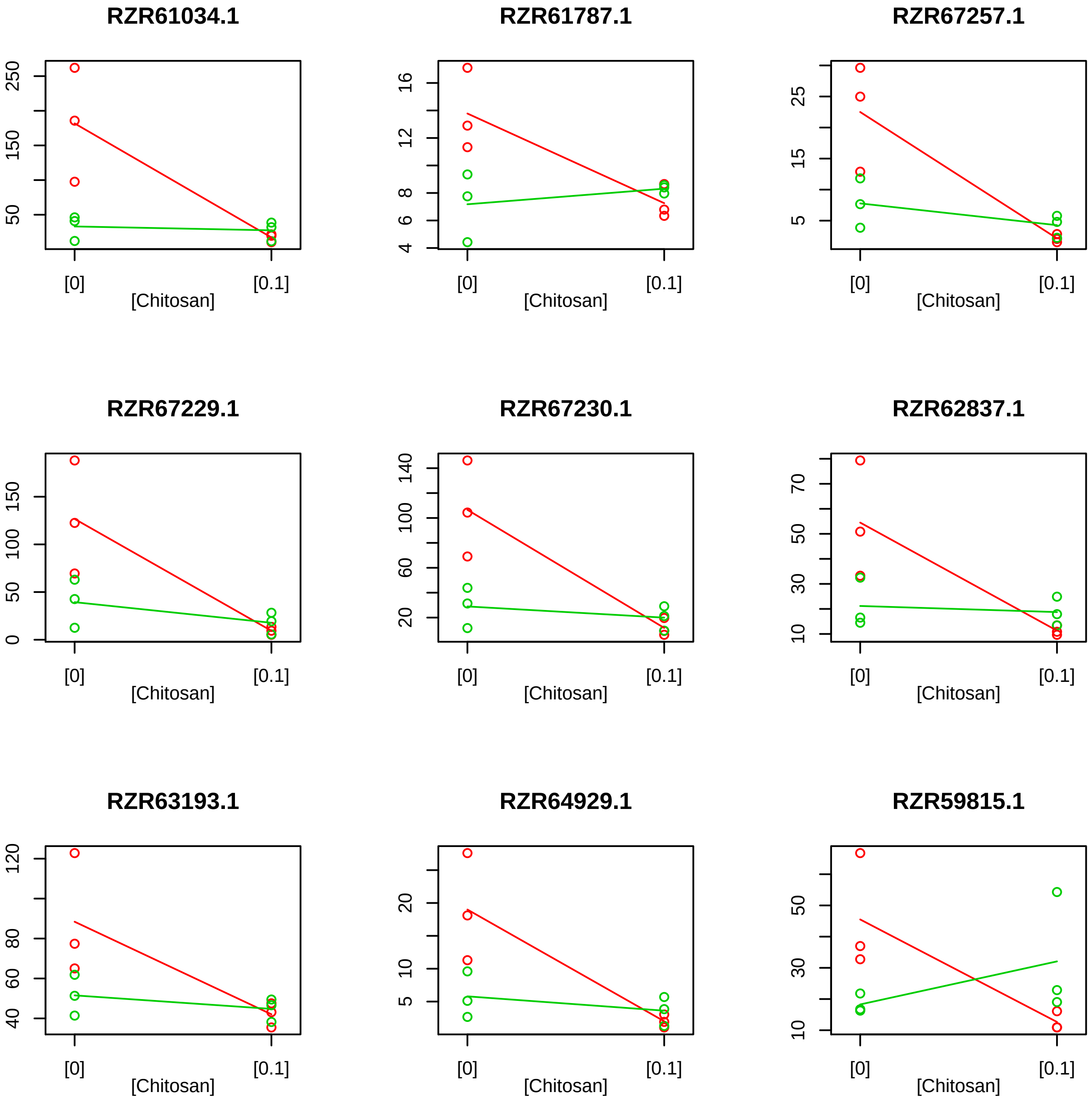
RZR61034.1
50
150
250
[0]
[0.1]
[Chitosan]
RZR61787.1
4
6
8
12
16
[0]
[0.1]
[Chitosan]
RZR67257.1
5
15
25
[0]
[0.1]
[Chitosan]
RZR67229.1
0
50
100
150
[0]
[0.1]
[Chitosan]
RZR67230.1
20
60
100
140
[0]
[0.1]
[Chitosan]
RZR62837.1
10
30
50
70
[0]
[0.1]
[Chitosan]
RZR63193.1
40
60
80
120
[0]
[0.1]
[Chitosan]
RZR64929.1
5
10
20
[0]
[0.1]
[Chitosan]
RZR59815.1
10
30
50
[0]
[0.1]
[Chitosan]

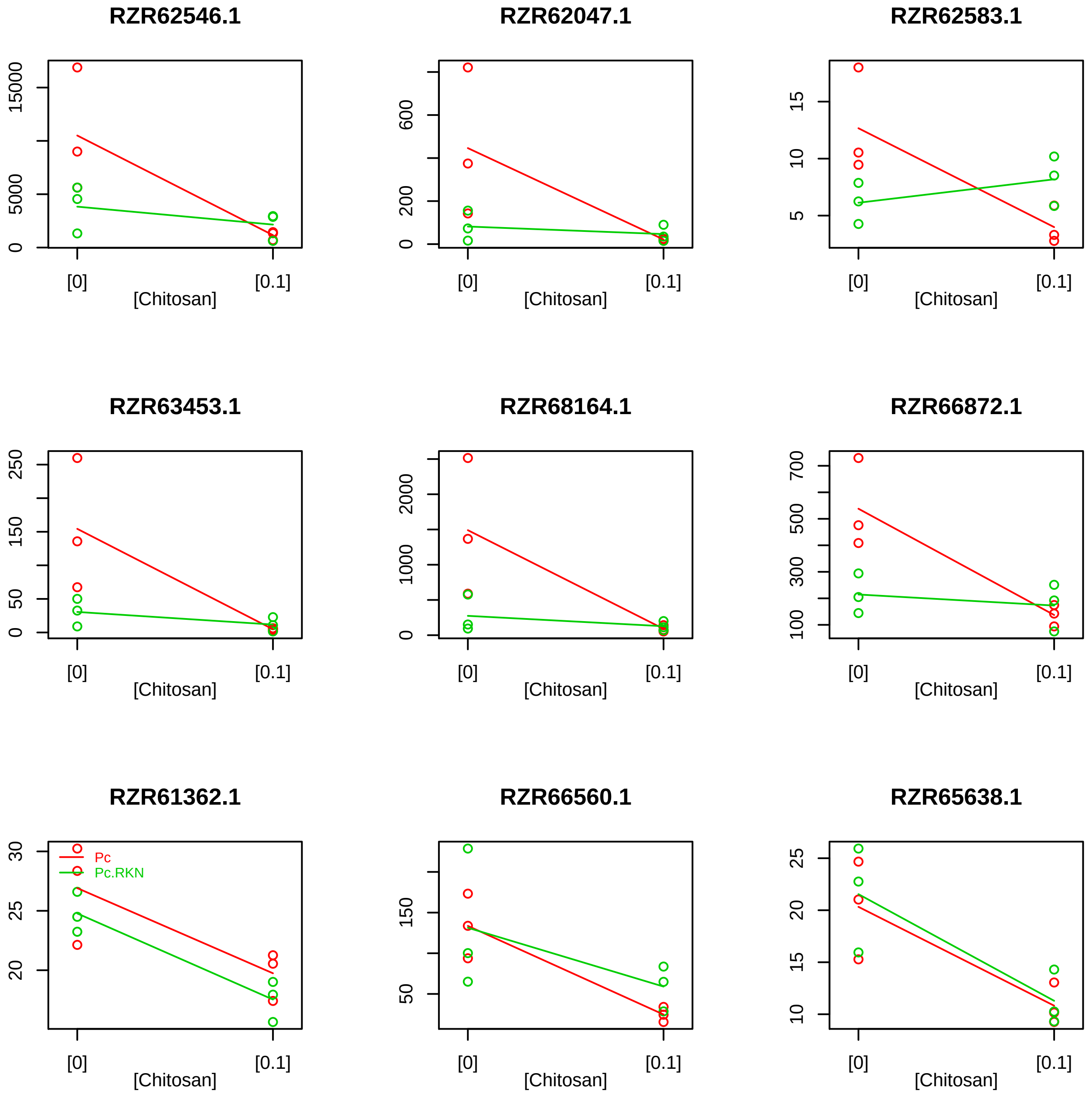

RZR62546.1
0
5000
15000
RZR62047.1
0
200
600
RZR62583.1
5
10
15
RZR63453.1
0
50
150
250
RZR68164.1
0
1000
2000
RZR66872.1
100
300
500
700
RZR61362.1
20
25
30
Pc
Pc.RKN
RZR66560.1
50
150
RZR65638.1
10
15
20
25
[0]
[0.1]
[Chitosan]

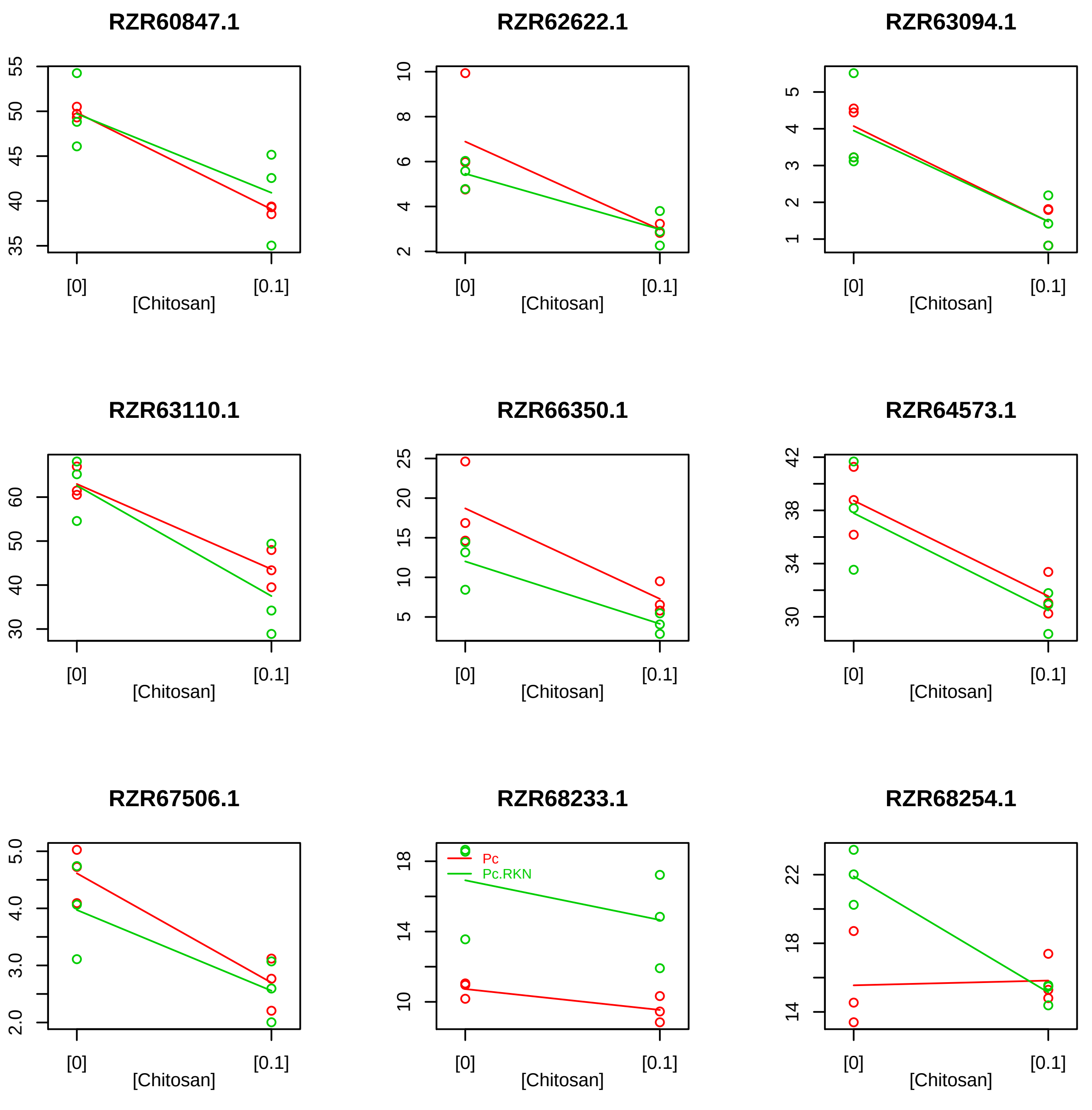
RZR60847.1
RZR62622.1
RZR63094.1
RZR63110.1
RZR66350.1
RZR64573.1
RZR67506.1
RZR68233.1
RZR68254.1
Pc
Pc.RKN
[0]
[0.1]
[Chitosan]

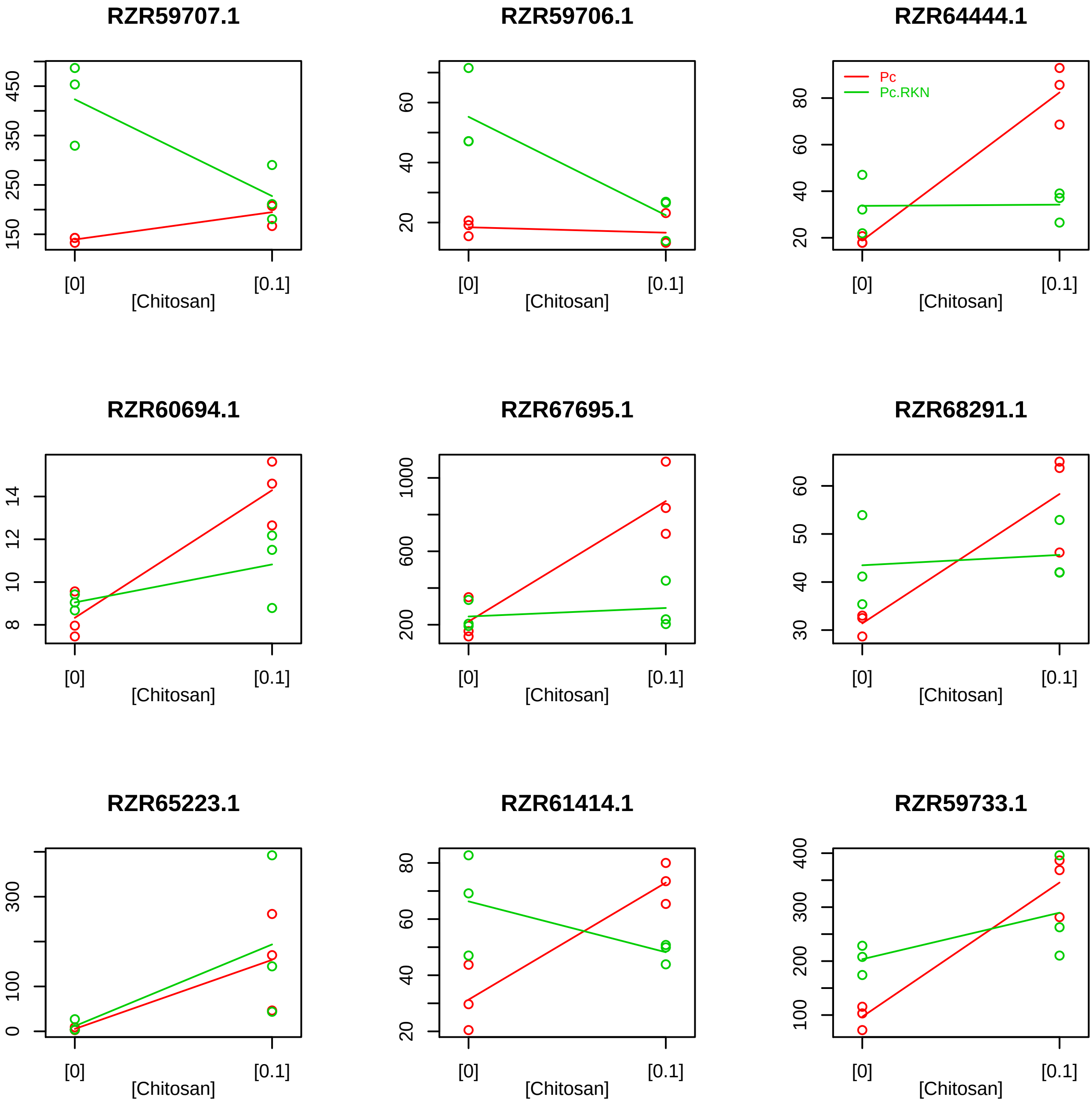
RZR59707.1
RZR59706.1
RZR64444.1
Pc
Pc.RKN
RZR60694.1
RZR67695.1
RZR68291.1
RZR65223.1
RZR61414.1
RZR59733.1
[0]
[0.1]
[Chitosan]

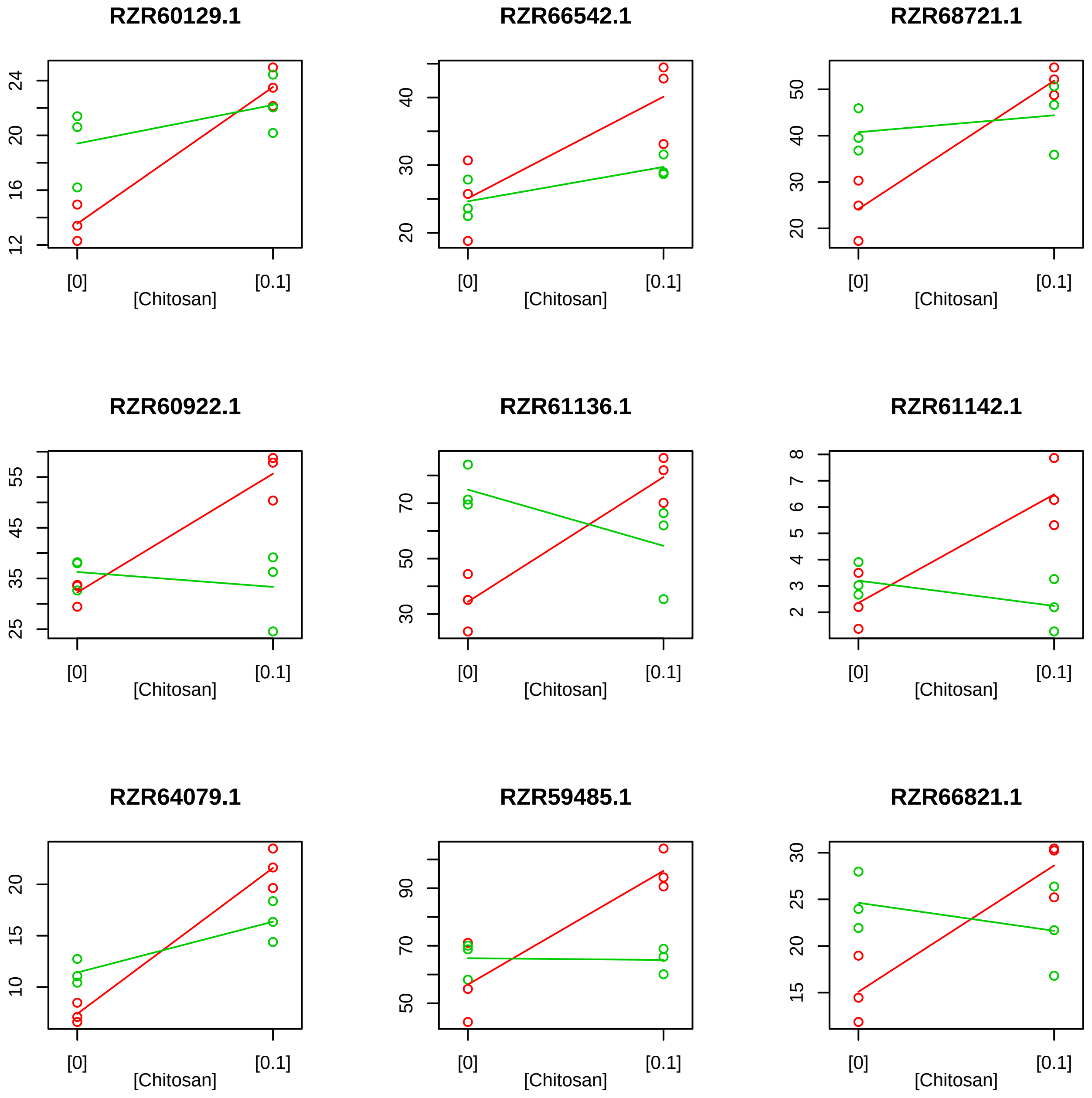
RZR60129.1
RZR66542.1
RZR68721.1
RZR60922.1
RZR61136.1
RZR61142.1
RZR64079.1
RZR59485.1
RZR66821.1
[0]
[0.1]
[Chitosan]

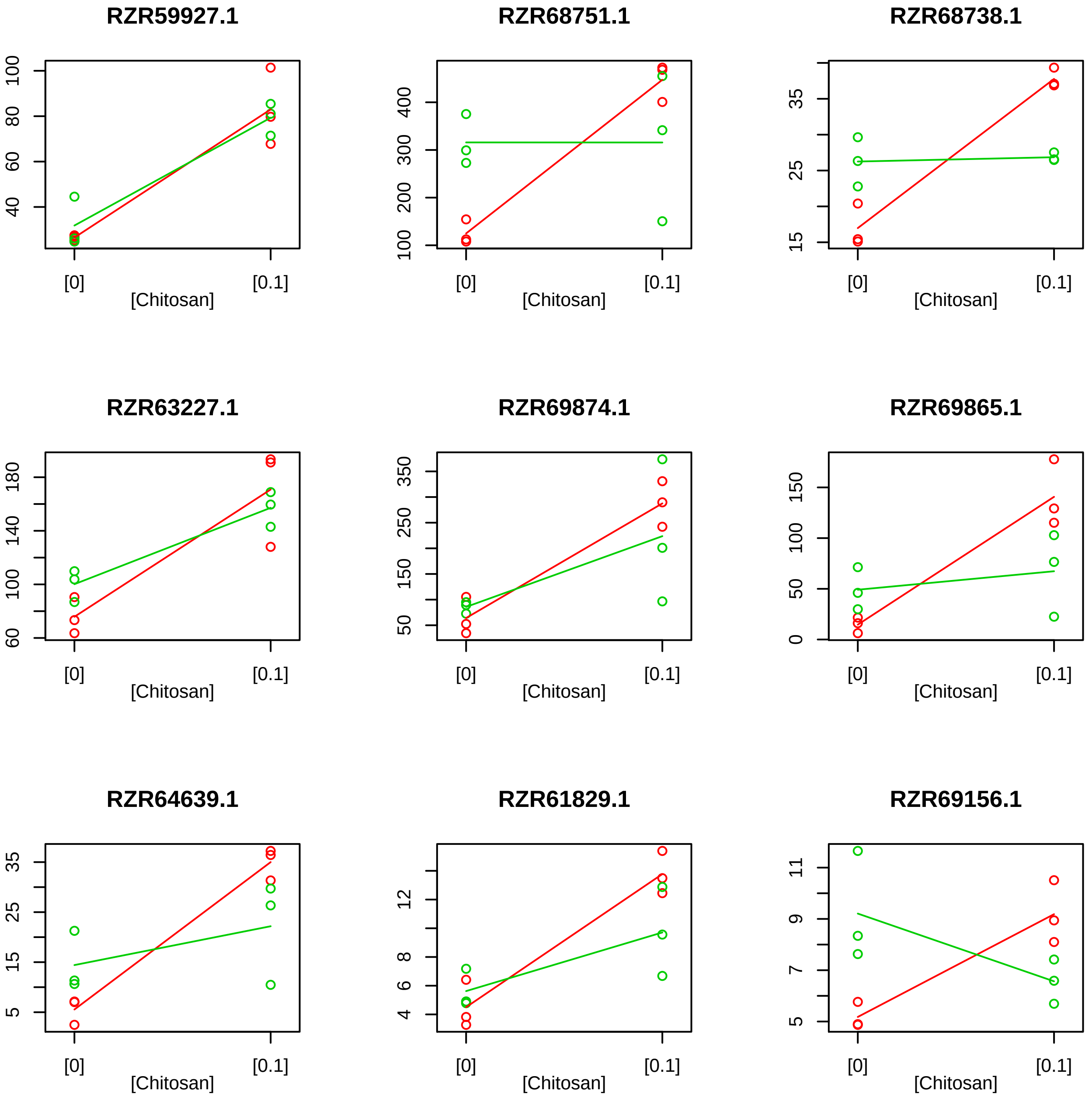
RZR59927.1
100
80
60
40
[0]
[0.1]
[Chitosan]
RZR68751.1
400
300
200
100
[0]
[0.1]
[Chitosan]
RZR68738.1
35
25
15
[0]
[0.1]
[Chitosan]
RZR63227.1
180
140
100
60
[0]
[0.1]
[Chitosan]
RZR69874.1
350
250
150
50
[0]
[0.1]
[Chitosan]
RZR69865.1
150
100
50
0
[0]
[0.1]
[Chitosan]
RZR64639.1
35
25
15
5
[0]
[0.1]
[Chitosan]
RZR61829.1
12
8
6
4
[0]
[0.1]
[Chitosan]
RZR69156.1
11
9
7
5
[0]
[0.1]
[Chitosan]
