# Supplementary material for: Chitosan modulates Pochonia chlamydosporia gene expression during nematode egg parasitism
Source: Environ Microbiol. 2021 Feb 5;23(9):4980–97. doi: 10.1111/1462-2920.15408 (PMC8518118; doi:10.1111/1462-2920.15408)
Supplement: Supplementary file 5 — Supplementary Fig. 4. Pc gene expression of 8 selected genes when the fungus colonizes banana roots. Treatments: Pc (Pc mycelium in MM), PcQ (Pc mycelium in MM amended with 0.1 mg·mL−1 chitosan), PcB (Pc mycelium growing in MM close to banana roots), PcBQ (Pc mycelium growing in MM amended with 0.1 mg·mL−1 chitosan close to banana roots), BPc (banana roots colonized by Pc) and BPcQ (banana roots colonized by Pc in medium amended with 0.1 mg·mL−1 chitosan). [file EMI-23-4980-s010.docx]

**Supplementary Figure 4.** Pc gene expression of 8 selected genes when the fungus colonizes banana roots. Treatments: Pc (Pc mycelium in MM), PcQ (Pc mycelium in MM amended with 0.1 mg·mL^-1^ chitosan), PcB (Pc mycelium growing in MM close to Banana roots), PcBQ (Pc mycelium growing in MM amended with 0.1 mg·mL^-1^ chitosan close to Banana roots), BPc (Banana roots colonized by Pc) and BPcQ (Banana roots colonized by Pc in medium amended with 0.1 mg·mL^-1^ chitosan).
