# Supplementary material for: Chitosan modulates Pochonia chlamydosporia gene expression during nematode egg parasitism
Source: Environ Microbiol. 2021 Feb 5;23(9):4980–97. doi: 10.1111/1462-2920.15408 (PMC8518118; doi:10.1111/1462-2920.15408)
Supplement: Supplementary file 6 — Supplementary Fig. 5. Workflow of the steps followed for the analysis and obtaining log2 fold change values from the raw data. [file EMI-23-4980-s007.docx]

**Supplementary Figure 5.** Workflow of the steps followed for the analysis and obtaining log2fc values from the raw data.
