# Supplementary material for: Chitosan modulates Pochonia chlamydosporia gene expression during nematode egg parasitism
Source: Environ Microbiol. 2021 Feb 5;23(9):4980–97. doi: 10.1111/1462-2920.15408 (PMC8518118; doi:10.1111/1462-2920.15408)
Supplement: Supplementary file 7 — Supplementary Table 1. Statistical analyses of 113 GO enriched Terms from Fig. 3. [file EMI-23-4980-s009.docx]

**Supplementary Table 1.** Statistical analyses of 113 GO enriched Terms from Figure 3.

|  |  |  |  | **MODEL** | | **p-values VARIABLES** | | | |
| --- | --- | --- | --- | --- | --- | --- | --- | --- | --- |
| **GO** | **GO.names** | **num.genes** | **CLUSTER** | **p-value** | **R-squared** | **beta0** | **RKN** | **Chitosan** | **ChitosanxRKN** |
| GO:0000796 | condensin complex | 3 | 1 | 0,0000 | 0,3313 | NA | 0,0011 | 0,0002 | 0,0001 |
| GO:0000916 | actomyosin contractile ring contraction | 4 | 1 | 0,0038 | 0,1706 | 0,0000 | NA | 0,0076 | 0,0041 |
| GO:0000917 | division septum assembly | 5 | 1 | 0,0054 | 0,1361 | 0,0000 | NA | 0,0137 | 0,0036 |
| GO:0002161 | aminoacyl-tRNA editing activity | 8 | 1 | 0,0053 | 0,1229 | 0,0000 | 0,0354 | 0,0007 | 0,0123 |
| GO:0003777 | microtubule motor activity | 22 | 1 | 0,0000 | 0,1013 | 0,0000 | 0,0012 | 0,0000 | 0,0000 |
| GO:0004822 | isoleucine-tRNA ligase activity | 3 | 1 | 0,0059 | 0,2312 | 0,0115 | 0,0302 | 0,0024 | 0,0430 |
| GO:0004829 | threonine-tRNA ligase activity | 3 | 1 | 0,0132 | 0,2012 | NA | 0,0465 | 0,0033 | 0,0152 |
| GO:0005200 | structural constituent of cytoskeleton | 7 | 1 | 0,0041 | 0,1022 | 0,0000 | NA | 0,0031 | 0,0070 |
| GO:0005933 | cellular bud | 11 | 1 | 0,0002 | 0,1029 | 0,0000 | NA | 0,0011 | 0,0002 |
| GO:0005935 | cellular bud neck | 8 | 1 | 0,0007 | 0,1170 | 0,0000 | NA | 0,0031 | 0,0006 |
| GO:0005952 | cAMP-dependent protein kinase complex | 2 | 1 | 0,0001 | 0,3480 | 0,0400 | NA | 0,0018 | 0,0016 |
| GO:0006428 | isoleucyl-tRNA aminoacylation | 3 | 1 | 0,0059 | 0,2312 | 0,0115 | 0,0302 | 0,0024 | 0,0430 |
| GO:0006435 | threonyl-tRNA aminoacylation | 3 | 1 | 0,0132 | 0,2012 | NA | 0,0465 | 0,0033 | 0,0152 |
| GO:0007076 | mitotic chromosome condensation | 3 | 1 | 0,0000 | 0,3313 | NA | 0,0011 | 0,0002 | 0,0001 |
| GO:0007096 | regulation of exit from mitosis | 6 | 1 | 0,0032 | 0,1279 | 0,0000 | NA | 0,0081 | 0,0024 |
| GO:0008017 | microtubule binding | 19 | 1 | 0,0000 | 0,1497 | 0,0000 | 0,0001 | 0,0000 | 0,0000 |
| GO:0008239 | dipeptidyl-peptidase activity | 4 | 1 | 0,0078 | 0,2370 | 0,0128 | NA | 0,0052 | 0,0793 |
| GO:0010458 | exit from mitosis | 6 | 1 | 0,0032 | 0,1279 | 0,0000 | NA | 0,0081 | 0,0024 |
| GO:0015074 | DNA integration | 10 | 1 | 0,0004 | 0,1036 | 0,0000 | NA | 0,0011 | 0,0007 |
| GO:0019566 | arabinose metabolic process | 7 | 1 | 0,0014 | 0,1325 | 0,0004 | NA | 0,0024 | NA |
| GO:0030261 | chromosome condensation | 3 | 1 | 0,0000 | 0,3313 | NA | 0,0011 | 0,0002 | 0,0001 |
| GO:0032465 | regulation of cytokinesis | 4 | 1 | 0,0041 | 0,1698 | 0,0000 | NA | 0,0090 | 0,0040 |
| GO:0032954 | regulation of cytokinetic process | 4 | 1 | 0,0041 | 0,1698 | 0,0000 | NA | 0,0090 | 0,0040 |
| GO:0032955 | regulation of division septum assembly | 4 | 1 | 0,0041 | 0,1698 | 0,0000 | NA | 0,0090 | 0,0040 |
| GO:0036046 | protein demalonylation | 2 | 1 | 0,0000 | 0,5298 | NA | NA | 0,0000 | 0,0019 |
| GO:0036047 | peptidyl-lysine demalonylation | 2 | 1 | 0,0000 | 0,5298 | NA | NA | 0,0000 | 0,0019 |
| GO:0036048 | protein desuccinylation | 2 | 1 | 0,0000 | 0,5298 | NA | NA | 0,0000 | 0,0019 |
| GO:0036049 | peptidyl-lysine desuccinylation | 2 | 1 | 0,0000 | 0,5298 | NA | NA | 0,0000 | 0,0019 |
| GO:0036054 | protein-malonyllysine demalonylase activity | 2 | 1 | 0,0000 | 0,5298 | NA | NA | 0,0000 | 0,0019 |
| GO:0036055 | protein-succinyllysine desuccinylase activity | 2 | 1 | 0,0000 | 0,5298 | NA | NA | 0,0000 | 0,0019 |
| GO:0036213 | contractile ring contraction | 4 | 1 | 0,0038 | 0,1706 | 0,0000 | NA | 0,0076 | 0,0041 |
| GO:0046373 | L-arabinose metabolic process | 7 | 1 | 0,0014 | 0,1325 | 0,0004 | NA | 0,0024 | NA |
| GO:0051302 | regulation of cell division | 4 | 1 | 0,0041 | 0,1698 | 0,0000 | NA | 0,0090 | 0,0040 |
| GO:0090529 | cell septum assembly | 5 | 1 | 0,0054 | 0,1361 | 0,0000 | NA | 0,0137 | 0,0036 |
| GO:0097599 | xylanase activity | 3 | 1 | 0,0000 | 0,4156 | NA | NA | 0,0001 | 0,0112 |
| GO:0099111 | microtubule-based transport | 3 | 1 | 0,0008 | 0,2434 | NA | 0,0544 | 0,0011 | 0,0018 |
| GO:0106074 | aminoacyl-tRNA metabolism involved in translational fidelity | 8 | 1 | 0,0053 | 0,1229 | 0,0000 | 0,0354 | 0,0007 | 0,0123 |
| GO:1901891 | regulation of cell septum assembly | 4 | 1 | 0,0041 | 0,1698 | 0,0000 | NA | 0,0090 | 0,0040 |
| GO:0000104 | succinate dehydrogenase activity | 6 | 2 | 0,0004 | 0,1340 | NA | NA | NA | 0,0006 |
| GO:0003954 | NADH dehydrogenase activity | 8 | 2 | 0,0000 | 0,1259 | NA | NA | NA | 0,0000 |
| GO:0003993 | acid phosphatase activity | 7 | 2 | 0,0044 | 0,1244 | 0,0000 | NA | 0,0899 | 0,0018 |
| GO:0005375 | copper ion transmembrane transporter activity | 4 | 2 | 0,0001 | 0,2818 | 0,0000 | 0,0014 | 0,0009 | 0,0001 |
| GO:0005385 | zinc ion transmembrane transporter activity | 3 | 2 | 0,0001 | 0,3180 | 0,0019 | 0,0051 | 0,0016 | 0,0031 |
| GO:0006743 | ubiquinone metabolic process | 10 | 2 | 0,0000 | 0,1279 | 0,0000 | 0,0080 | 0,0001 | 0,0000 |
| GO:0006744 | ubiquinone biosynthetic process | 10 | 2 | 0,0000 | 0,1279 | 0,0000 | 0,0080 | 0,0001 | 0,0000 |
| GO:0006825 | copper ion transport | 5 | 2 | 0,0000 | 0,2705 | 0,0000 | 0,0006 | 0,0001 | 0,0000 |
| GO:0006829 | zinc ion transport | 3 | 2 | 0,0001 | 0,3180 | 0,0019 | 0,0051 | 0,0016 | 0,0031 |
| GO:0008137 | NADH dehydrogenase (ubiquinone) activity | 8 | 2 | 0,0000 | 0,1259 | NA | NA | NA | 0,0000 |
| GO:0008169 | C-methyltransferase activity | 6 | 2 | 0,0067 | 0,1593 | 0,0000 | 0,0173 | 0,0036 | 0,0029 |
| GO:0008199 | ferric iron binding | 6 | 2 | 0,0014 | 0,1415 | 0,0034 | 0,0487 | 0,0073 | 0,0004 |
| GO:0008425 | 2-polyprenyl-6-methoxy-1,4-benzoquinone methyltransferase activity | 2 | 2 | 0,0008 | 0,3211 | 0,0000 | 0,0059 | 0,0026 | 0,0042 |
| GO:0009228 | thiamine biosynthetic process | 4 | 2 | 0,0142 | 0,1688 | 0,0000 | 0,0161 | 0,0164 | 0,0028 |
| GO:0009295 | nucleoid | 5 | 2 | 0,0008 | 0,1260 | 0,0023 | NA | NA | 0,0011 |
| GO:0016679 | oxidoreductase activity, acting on diphenols and related substances as donors | 5 | 2 | 0,0005 | 0,1255 | 0,0134 | NA | NA | 0,0007 |
| GO:0030580 | quinone cofactor methyltransferase activity | 2 | 2 | 0,0008 | 0,3211 | 0,0000 | 0,0059 | 0,0026 | 0,0042 |
| GO:0031312 | extrinsic component of organelle membrane | 7 | 2 | 0,0015 | 0,1468 | 0,0000 | 0,0397 | 0,0003 | 0,0014 |
| GO:0031314 | extrinsic component of mitochondrial inner membrane | 6 | 2 | 0,0000 | 0,2020 | 0,0000 | 0,0090 | 0,0000 | 0,0000 |
| GO:0035434 | copper ion transmembrane transport | 4 | 2 | 0,0001 | 0,2818 | 0,0000 | 0,0014 | 0,0009 | 0,0001 |
| GO:0042645 | mitochondrial nucleoid | 5 | 2 | 0,0008 | 0,1260 | 0,0023 | NA | NA | 0,0011 |
| GO:0042724 | thiamine-containing compound biosynthetic process | 4 | 2 | 0,0142 | 0,1688 | 0,0000 | 0,0161 | 0,0164 | 0,0028 |
| GO:0047617 | acyl-CoA hydrolase activity | 9 | 2 | 0,0006 | 0,1228 | 0,0000 | 0,0192 | 0,0346 | 0,0002 |
| GO:0050136 | NADH dehydrogenase (quinone) activity | 8 | 2 | 0,0000 | 0,1259 | NA | NA | NA | 0,0000 |
| GO:0070814 | hydrogen sulfide biosynthetic process | 3 | 2 | 0,0040 | 0,1757 | 0,0689 | 0,0112 | 0,0749 | NA |
| GO:0071577 | zinc ion transmembrane transport | 3 | 2 | 0,0001 | 0,3180 | 0,0019 | 0,0051 | 0,0016 | 0,0031 |
| GO:0097428 | protein maturation by iron-sulfur cluster transfer | 6 | 2 | 0,0002 | 0,1888 | 0,0000 | 0,0266 | 0,0003 | 0,0002 |
| GO:1901661 | quinone metabolic process | 10 | 2 | 0,0000 | 0,1279 | 0,0000 | 0,0080 | 0,0001 | 0,0000 |
| GO:1901663 | quinone biosynthetic process | 10 | 2 | 0,0000 | 0,1279 | 0,0000 | 0,0080 | 0,0001 | 0,0000 |
| GO:0000225 | N-acetylglucosaminylphosphatidylinositol deacetylase activity | 2 | 3 | 0,0027 | 0,2694 | 0,0147 | 0,0111 | 0,0107 | 0,0102 |
| GO:0000315 | organellar large ribosomal subunit | 6 | 3 | 0,0029 | 0,1208 | 0,0000 | 0,0549 | 0,0007 | 0,0020 |
| GO:0004364 | glutathione transferase activity | 5 | 3 | 0,0009 | 0,2507 | 0,0000 | 0,0069 | 0,0028 | 0,0572 |
| GO:0004609 | phosphatidylserine decarboxylase activity | 5 | 3 | 0,0013 | 0,1417 | 0,0000 | 0,0392 | 0,0048 | NA |
| GO:0005762 | mitochondrial large ribosomal subunit | 6 | 3 | 0,0029 | 0,1208 | 0,0000 | 0,0549 | 0,0007 | 0,0020 |
| GO:0006480 | N-terminal protein amino acid methylation | 4 | 3 | 0,0004 | 0,2071 | 0,0034 | 0,0947 | 0,0017 | NA |
| GO:0006490 | oligosaccharide-lipid intermediate biosynthetic process | 8 | 3 | 0,0047 | 0,1073 | 0,0000 | 0,0035 | 0,0037 | 0,0212 |
| GO:0008479 | queuine tRNA-ribosyltransferase activity | 2 | 3 | 0,0137 | 0,2373 | 0,0034 | 0,0487 | 0,0096 | 0,0137 |
| GO:0008535 | respiratory chain complex IV assembly | 6 | 3 | 0,0009 | 0,1431 | 0,0000 | 0,0242 | 0,0002 | 0,0015 |
| GO:0010257 | NADH dehydrogenase complex assembly | 3 | 3 | 0,0015 | 0,2124 | 0,0001 | 0,0153 | 0,0018 | 0,0265 |
| GO:0015680 | protein maturation by copper ion transfer | 3 | 3 | 0,0115 | 0,1769 | 0,0000 | 0,0425 | 0,0043 | 0,0089 |
| GO:0018013 | N-terminal peptidyl-glycine methylation | 2 | 3 | 0,0010 | 0,2199 | 0,0316 | NA | 0,0054 | NA |
| GO:0018027 | peptidyl-lysine dimethylation | 2 | 3 | 0,0010 | 0,2199 | 0,0316 | NA | 0,0054 | NA |
| GO:0018201 | peptidyl-glycine modification | 2 | 3 | 0,0010 | 0,2199 | 0,0316 | NA | 0,0054 | NA |
| GO:0030366 | molybdopterin synthase activity | 2 | 3 | 0,0067 | 0,2642 | 0,0058 | 0,0407 | 0,0075 | 0,0163 |
| GO:0031391 | Elg1 RFC-like complex | 2 | 3 | 0,0002 | 0,3351 | 0,0001 | 0,0238 | 0,0018 | 0,0745 |
| GO:0032981 | mitochondrial respiratory chain complex I assembly | 3 | 3 | 0,0015 | 0,2124 | 0,0001 | 0,0153 | 0,0018 | 0,0265 |
| GO:0033108 | mitochondrial respiratory chain complex assembly | 18 | 3 | 0,0000 | 0,1015 | 0,0000 | 0,0008 | 0,0000 | 0,0000 |
| GO:0033179 | proton-transporting V-type ATPase, V0 domain | 7 | 3 | 0,0019 | 0,1218 | 0,0000 | 0,0110 | 0,0018 | 0,0782 |
| GO:0033617 | mitochondrial respiratory chain complex IV assembly | 6 | 3 | 0,0009 | 0,1431 | 0,0000 | 0,0242 | 0,0002 | 0,0015 |
| GO:0042181 | ketone biosynthetic process | 12 | 3 | 0,0000 | 0,1256 | 0,0000 | 0,0203 | 0,0000 | 0,0000 |
| GO:0055070 | copper ion homeostasis | 3 | 3 | 0,0020 | 0,2316 | 0,0000 | 0,0165 | 0,0018 | 0,0298 |
| GO:0060303 | regulation of nucleosome density | 2 | 3 | 0,0001 | 0,3221 | 0,0000 | 0,0009 | NA | NA |
| GO:0071885 | N-terminal protein N-methyltransferase activity | 2 | 3 | 0,0010 | 0,2199 | 0,0316 | NA | 0,0054 | NA |
| GO:0101030 | tRNA-guanine transglycosylation | 2 | 3 | 0,0137 | 0,2373 | 0,0034 | 0,0487 | 0,0096 | 0,0137 |
| GO:0004767 | sphingomyelin phosphodiesterase activity | 3 | 4 | 0,0029 | 0,3506 | NA | 0,0035 | 0,0206 | 0,0404 |
| GO:0006549 | isoleucine metabolic process | 7 | 4 | 0,0127 | 0,1075 | NA | 0,0119 | 0,0035 | 0,0057 |
| GO:0006684 | sphingomyelin metabolic process | 3 | 4 | 0,0029 | 0,3506 | NA | 0,0035 | 0,0206 | 0,0404 |
| GO:0006685 | sphingomyelin catabolic process | 3 | 4 | 0,0029 | 0,3506 | NA | 0,0035 | 0,0206 | 0,0404 |
| GO:0009062 | fatty acid catabolic process | 5 | 4 | 0,0001 | 0,1843 | 0,0084 | 0,0007 | 0,0241 | NA |
| GO:0009097 | isoleucine biosynthetic process | 7 | 4 | 0,0127 | 0,1075 | NA | 0,0119 | 0,0035 | 0,0057 |
| GO:0030149 | sphingolipid catabolic process | 7 | 4 | 0,0004 | 0,2271 | NA | 0,0012 | 0,0007 | 0,0049 |
| GO:0032968 | positive regulation of transcription elongation from RNA polymerase II promoter | 7 | 4 | 0,0024 | 0,1297 | 0,0006 | 0,0007 | 0,0036 | 0,0043 |
| GO:0034243 | regulation of transcription elongation from RNA polymerase II promoter | 8 | 4 | 0,0011 | 0,1228 | 0,0001 | 0,0005 | 0,0013 | 0,0016 |
| GO:0046466 | membrane lipid catabolic process | 7 | 4 | 0,0004 | 0,2271 | NA | 0,0012 | 0,0007 | 0,0049 |
| GO:0006099 | tricarboxylic acid cycle | 22 | 5 | 0,0000 | 0,1069 | 0,0000 | NA | NA | 0,0000 |
| GO:0009060 | aerobic respiration | 24 | 5 | 0,0000 | 0,1003 | 0,0000 | NA | 0,0870 | 0,0010 |
| GO:0031072 | heat shock protein binding | 5 | 5 | 0,0003 | 0,1535 | 0,0000 | NA | 0,0007 | NA |
| GO:0043605 | cellular amide catabolic process | 4 | 5 | 0,0009 | 0,2050 | 0,0000 | 0,0185 | 0,0067 | NA |
| GO:0045239 | tricarboxylic acid cycle enzyme complex | 4 | 5 | 0,0002 | 0,2385 | 0,0000 | 0,0593 | 0,0007 | NA |
| GO:0016977 | chitosanase activity | 11 | 6 | 0,0000 | 0,2828 | 0,0000 | NA | 0,0001 | 0,0001 |
| GO:0052761 | exo-1,4-beta-D-glucosaminidase activity | 2 | 6 | 0,0000 | 0,4904 | 0,0000 | 0,0137 | 0,0000 | NA |
| GO:0006098 | pentose-phosphate shunt | 7 | 7 | 0,0007 | 0,1112 | 0,0000 | 0,0012 | NA | NA |
| GO:0006740 | NADPH regeneration | 7 | 7 | 0,0007 | 0,1112 | 0,0000 | 0,0012 | NA | NA |
| GO:0004181 | metallocarboxypeptidase activity | 7 | 8 | 0,0002 | 0,1034 | 0,0000 | 0,0005 | NA | NA |
| GO:0005199 | structural constituent of cell wall | 6 | 9 | 0,0007 | 0,1265 | 0,0000 | 0,0014 | NA | NA |
