# Supplementary material for: Chitosan modulates Pochonia chlamydosporia gene expression during nematode egg parasitism
Source: Environ Microbiol. 2021 Feb 5;23(9):4980–97. doi: 10.1111/1462-2920.15408 (PMC8518118; doi:10.1111/1462-2920.15408)
Supplement: Supplementary file 8 — Supplementary Table 2. 113 GO enriched Terms from Fig. 3: ID, Description and genes included. [file EMI-23-4980-s004.docx]

**Supplementary Table 2.** 113 GO enriched Terms from Figure 3: ID, Description and genes included.

| **GO ID** | **GO Description** | **GO Genes Included** |
| --- | --- | --- |
| GO:0000104 | succinate dehydrogenase activity | RZR68187.1,RZR63823.1,RZR62514.1,RZR60007.1,RZR60004.1 |
| GO:0000225 | N-acetylglucosaminylphosphatidylinositol deacetylase activity | RZR65549.1,RZR64552.1 |
| GO:0000315 | organellar large ribosomal subunit | RZR67887.1,RZR66506.1,RZR64382.1,RZR63665.1,RZR60179.1,RZR59994.1 |
| GO:0000796 | condensin complex | RZR68265.1,RZR67103.1,RZR64937.1 |
| GO:0000916 | actomyosin contractile ring contraction | RZR69077.1,RZR66739.1,RZR65927.1,RZR65855.1 |
| GO:0000917 | division septum assembly | RZR59323.1,RZR66739.1,RZR65927.1,RZR65855.1,RZR63618.1 |
| GO:0002161 | aminoacyl-tRNA editing activity | RZR69911.1,RZR67956.1,RZR64344.1,RZR63849.1,RZR61589.1,RZR61190.1,RZR60480.1 |
| GO:0003777 | microtubule motor activity | RZR59488.1,RZR69421.1,RZR68897.1,RZR68402.1,RZR67519.1,RZR66943.1,RZR66880.1,RZR66431.1,RZR66163.1,RZR65030.1,RZR64440.1,RZR64429.1,RZR64224.1,RZR64218.1,RZR63172.1,RZR62373.1,RZR62097.1,RZR61600.1,RZR60631.1 |
| GO:0003954 | NADH dehydrogenase activity | RZR67747.1,RZR67714.1,RZR67091.1,RZR66584.1,RZR65807.1,RZR65658.1,RZR63682.1 |
| GO:0003993 | acid phosphatase activity | RZR59816.1,RZR68341.1,RZR67516.1,RZR67148.1,RZR62665.1,RZR61721.1 |
| GO:0004181 | metallocarboxypeptidase activity | RZR68343.1,RZR66784.1,RZR65715.1,RZR65711.1,RZR63096.1,RZR61143.1,RZR59918.1 |
| GO:0004364 | glutathione transferase activity | RZR68352.1,RZR68123.1,RZR60846.1 |
| GO:0004609 | phosphatidylserine decarboxylase activity | RZR68025.1,RZR66653.1,RZR65013.1,RZR63420.1,RZR63419.1 |
| GO:0004767 | sphingomyelin phosphodiesterase activity | RZR67459.1,RZR60731.1 |
| GO:0004822 | isoleucine-tRNA ligase activity | RZR69911.1,RZR63849.1,RZR60480.1 |
| GO:0004829 | threonine-tRNA ligase activity | RZR68624.1,RZR65353.1,RZR61995.1 |
| GO:0005199 | structural constituent of cell wall | RZR59839.1,RZR69569.1,RZR67696.1,RZR62766.1,RZR60003.1 |
| GO:0005200 | structural constituent of cytoskeleton | RZR69077.1,RZR66308.1,RZR65128.1,RZR64063.1,RZR63853.1,RZR60700.1,RZR60428.1 |
| GO:0005375 | copper ion transmembrane transporter activity | RZR64616.1,RZR62374.1,RZR61814.1,RZR61718.1 |
| GO:0005385 | zinc ion transmembrane transporter activity | RZR70090.1,RZR63877.1 |
| GO:0005762 | mitochondrial large ribosomal subunit | RZR67887.1,RZR66506.1,RZR64382.1,RZR63665.1,RZR60179.1,RZR59994.1 |
| GO:0005933 | cellular bud | RZR70101.1,RZR69095.1,RZR69077.1,RZR68496.1,RZR66583.1,RZR66561.1,RZR65927.1,RZR65855.1,RZR63749.1,RZR63200.1,RZR61072.1 |
| GO:0005935 | cellular bud neck | RZR69077.1,RZR68496.1,RZR66561.1,RZR65927.1,RZR65855.1,RZR63749.1,RZR63200.1,RZR61072.1 |
| GO:0005952 | cAMP-dependent protein kinase complex | RZR66548.1,RZR60342.1 |
| GO:0006098 | pentose-phosphate shunt | RZR69380.1,RZR68968.1,RZR68789.1,RZR67228.1,RZR66497.1,RZR65586.1,RZR62817.1 |
| GO:0006099 | tricarboxylic acid cycle | RZR70262.1,RZR69977.1,RZR69573.1,RZR69120.1,RZR68187.1,RZR67838.1,RZR67100.1,RZR66272.1,RZR66094.1,RZR64290.1,RZR64130.1,RZR63707.1,RZR63280.1,RZR62514.1,RZR62185.1,RZR61818.1,RZR61194.1,RZR60653.1,RZR60007.1,RZR60004.1 |
| GO:0006428 | isoleucyl-tRNA aminoacylation | RZR69911.1,RZR63849.1,RZR60480.1 |
| GO:0006435 | threonyl-tRNA aminoacylation | RZR68624.1,RZR65353.1,RZR61995.1 |
| GO:0006480 | N-terminal protein amino acid methylation | RZR63791.1,RZR60150.1,RZR60149.1 |
| GO:0006490 | oligosaccharide-lipid intermediate biosynthetic process | RZR59858.1,RZR59274.1,RZR69743.1,RZR67532.1,RZR67129.1,RZR66812.1,RZR62516.1,RZR61871.1 |
| GO:0006549 | isoleucine metabolic process | RZR59853.1,RZR68694.1,RZR68693.1,RZR65095.1,RZR63884.1,RZR59872.1 |
| GO:0006684 | sphingomyelin metabolic process | RZR67459.1,RZR60731.1 |
| GO:0006685 | sphingomyelin catabolic process | RZR67459.1,RZR60731.1 |
| GO:0006740 | NADPH regeneration | RZR69380.1,RZR68968.1,RZR68789.1,RZR67228.1,RZR66497.1,RZR65586.1,RZR62817.1 |
| GO:0006743 | ubiquinone metabolic process | RZR70285.1,RZR69906.1,RZR68209.1,RZR67748.1,RZR67355.1,RZR66583.1,RZR66085.1,RZR64164.1,RZR64045.1,RZR60204.1 |
| GO:0006744 | ubiquinone biosynthetic process | RZR70285.1,RZR69906.1,RZR68209.1,RZR67748.1,RZR67355.1,RZR66583.1,RZR66085.1,RZR64164.1,RZR64045.1,RZR60204.1 |
| GO:0006825 | copper ion transport | RZR68882.1,RZR64616.1,RZR62374.1,RZR61814.1,RZR61718.1 |
| GO:0006829 | zinc ion transport | RZR70090.1,RZR63877.1 |
| GO:0007076 | mitotic chromosome condensation | RZR68265.1,RZR67103.1,RZR64937.1 |
| GO:0007096 | regulation of exit from mitosis | RZR67787.1,RZR66739.1,RZR65927.1,RZR65855.1,RZR61515.1,RZR61072.1 |
| GO:0008017 | microtubule binding | RZR68897.1,RZR68695.1,RZR68402.1,RZR67519.1,RZR67157.1,RZR67085.1,RZR67022.1,RZR66431.1,RZR66370.1,RZR66163.1,RZR65030.1,RZR64440.1,RZR64429.1,RZR63172.1,RZR62373.1,RZR61600.1,RZR60631.1 |
| GO:0008137 | NADH dehydrogenase (ubiquinone) activity | RZR67747.1,RZR67714.1,RZR67091.1,RZR66584.1,RZR65807.1,RZR65658.1,RZR63682.1 |
| GO:0008169 | C-methyltransferase activity | RZR68375.1,RZR67748.1,RZR61514.1,RZR60204.1 |
| GO:0008199 | ferric iron binding | RZR59710.1,RZR59623.1,RZR59593.1,RZR65090.1,RZR63248.1,RZR62779.1 |
| GO:0008239 | dipeptidyl-peptidase activity | RZR68809.1,RZR67873.1 |
| GO:0008425 | 2-polyprenyl-6-methoxy-1,4-benzoquinone methyltransferase activity | RZR67748.1,RZR60204.1 |
| GO:0008479 | queuine tRNA-ribosyltransferase activity | RZR62920.1,RZR61672.1 |
| GO:0008535 | respiratory chain complex IV assembly | RZR69137.1,RZR68882.1,RZR66450.1,RZR66266.1,RZR64278.1,RZR62474.1 |
| GO:0009060 | aerobic respiration | RZR70262.1,RZR70107.1,RZR69977.1,RZR69573.1,RZR69120.1,RZR68187.1,RZR67838.1,RZR67100.1,RZR66272.1,RZR66094.1,RZR64290.1,RZR64130.1,RZR63707.1,RZR63280.1,RZR62514.1,RZR62185.1,RZR61818.1,RZR61194.1,RZR60653.1,RZR60342.1,RZR60007.1,RZR60004.1 |
| GO:0009062 | fatty acid catabolic process | RZR69117.1,RZR66713.1,RZR65753.1,RZR65116.1,RZR60882.1 |
| GO:0009097 | isoleucine biosynthetic process | RZR59853.1,RZR68694.1,RZR68693.1,RZR65095.1,RZR63884.1,RZR59872.1 |
| GO:0009228 | thiamine biosynthetic process | RZR65191.1,RZR60779.1,RZR60251.1,RZR60249.1 |
| GO:0009295 | nucleoid | RZR66271.1,RZR65212.1,RZR64130.1,RZR62185.1,RZR59872.1 |
| GO:0010257 | NADH dehydrogenase complex assembly | RZR68001.1,RZR65097.1,RZR60816.1, |
| GO:0010458 | exit from mitosis | RZR67787.1,RZR66739.1,RZR65927.1,RZR65855.1,RZR61515.1,RZR61072.1 |
| GO:0015074 | DNA integration | RZR59233.1,RZR69746.1,RZR68761.1,RZR68760.1,RZR67010.1,RZR65585.1,RZR61377.1,RZR61271.1 |
| GO:0015680 | protein maturation by copper ion transfer | RZR68882.1,RZR64616.1,RZR60851.1 |
| GO:0016679 | oxidoreductase activity, acting on diphenols and related substances as donors | RZR70136.1,RZR70107.1,RZR65829.1,RZR65147.1,RZR63058.1 |
| GO:0016977 | chitosanase activity | RZR70313.1,RZR68649.1,RZR68136.1,RZR64948.1,RZR63795.1,RZR62940.1,RZR61845.1,RZR60235.1 |
| GO:0018013 | N-terminal peptidyl-glycine methylation | RZR60150.1,RZR60149.1 |
| GO:0018027 | peptidyl-lysine dimethylation | RZR60150.1,RZR60149.1 |
| GO:0018201 | peptidyl-glycine modification | RZR60150.1,RZR60149.1 |
| GO:0019566 | arabinose metabolic process | RZR69616.1,RZR69214.1,RZR67366.1,RZR64345.1,RZR60553.1 |
| GO:0030149 | sphingolipid catabolic process | RZR67459.1,RZR60731.1,RZR60128.1,RZR59887.1 |
| GO:0030261 | chromosome condensation | RZR68265.1,RZR67103.1,RZR64937.1 |
| GO:0030366 | molybdopterin synthase activity | RZR59201.1,RZR63335.1 |
| GO:0030580 | quinone cofactor methyltransferase activity | RZR67748.1,RZR60204.1 |
| GO:0031072 | heat shock protein binding | RZR68266.1,RZR67904.1,RZR66957.1,RZR65045.1,RZR60856.1 |
| GO:0031312 | extrinsic component of organelle membrane | RZR70285.1,RZR68209.1,RZR67748.1,RZR66095.1,RZR64164.1,RZR64045.1,RZR60204.1 |
| GO:0031314 | extrinsic component of mitochondrial inner membrane | RZR70285.1,RZR68209.1,RZR67748.1,RZR64164.1,RZR64045.1,RZR60204.1 |
| GO:0031391 | Elg1 RFC-like complex | RZR59249.1,RZR66381.1 |
| GO:0032465 | regulation of cytokinesis | RZR66739.1,RZR65927.1,RZR65855.1,RZR63618.1 |
| GO:0032954 | regulation of cytokinetic process | RZR66739.1,RZR65927.1,RZR65855.1,RZR63618.1 |
| GO:0032955 | regulation of division septum assembly | RZR66739.1,RZR65927.1,RZR65855.1,RZR63618.1 |
| GO:0032968 | positive regulation of transcription elongation from RNA polymerase II promoter | RZR59236.1,RZR66609.1,RZR64896.1,RZR64451.1,RZR63202.1,RZR63189.1,RZR62822.1 |
| GO:0032981 | mitochondrial respiratory chain complex I assembly | RZR68001.1,RZR65097.1,RZR60816.1 |
| GO:0033108 | mitochondrial respiratory chain complex assembly | RZR70107.1,RZR69749.1,RZR69137.1,RZR69061.1,RZR68882.1,RZR68373.1,RZR68001.1,RZR67704.1,RZR66683.1,RZR66450.1,RZR66266.1,RZR65097.1,RZR64278.1,RZR62474.1,RZR61807.1,RZR60816.1 |
| GO:0033179 | proton-transporting V-type ATPase, V0 domain | RZR68553.1,RZR68506.1,RZR68202.1,RZR66166.1,RZR65869.1,RZR64725.1,RZR60720.1 |
| GO:0033617 | mitochondrial respiratory chain complex IV assembly | RZR69137.1,RZR68882.1,RZR66450.1,RZR66266.1,RZR64278.1,RZR62474.1 |
| GO:0034243 | regulation of transcription elongation from RNA polymerase II promoter | RZR59236.1,RZR69964.1,RZR66609.1,RZR64896.1,RZR64451.1,RZR63202.1,RZR63189.1,RZR62822.1 |
| GO:0035434 | copper ion transmembrane transport | RZR64616.1,RZR62374.1,RZR61814.1,RZR61718.1 |
| GO:0036046 | protein demalonylation | RZR65109.1,RZR65108.1 |
| GO:0036047 | peptidyl-lysine demalonylation | RZR65109.1,RZR65108.1 |
| GO:0036048 | protein desuccinylation | RZR65109.1,RZR65108.1 |
| GO:0036049 | peptidyl-lysine desuccinylation | RZR65109.1,RZR65108.1 |
| GO:0036054 | protein-malonyllysine demalonylase activity | RZR65109.1,RZR65108.1 |
| GO:0036055 | protein-succinyllysine desuccinylase activity | RZR65109.1,RZR65108.1 |
| GO:0036213 | contractile ring contraction | RZR69077.1,RZR66739.1,RZR65927.1,RZR65855.1 |
| GO:0042181 | ketone biosynthetic process | RZR70285.1,RZR69906.1,RZR68209.1,RZR68199.1,RZR67748.1,RZR67355.1,RZR66583.1,RZR66085.1,RZR64164.1,RZR64045.1,RZR62263.1,RZR60204.1 |
| GO:0042645 | mitochondrial nucleoid | RZR66271.1,RZR65212.1,RZR64130.1,RZR62185.1,RZR59872.1 |
| GO:0042724 | thiamine-containing compound biosynthetic process | RZR65191.1,RZR60779.1,RZR60251.1,RZR60249.1 |
| GO:0043605 | cellular amide catabolic process | RZR59447.1,RZR70314.1,RZR68059.1,RZR61246.1 |
| GO:0045239 | tricarboxylic acid cycle enzyme complex | RZR70262.1,RZR66094.1,RZR64130.1,RZR61818.1 |
| GO:0046373 | L-arabinose metabolic process | RZR69616.1,RZR69214.1,RZR67366.1,RZR64345.1,RZR60553.1 |
| GO:0046466 | membrane lipid catabolic process | RZR67459.1,RZR60731.1,RZR60128.1,RZR59887.1 |
| GO:0047617 | acyl-CoA hydrolase activity | RZR68152.1,RZR66949.1,RZR66013.1,RZR65453.1,RZR65298.1,RZR65271.1,RZR63339.1,RZR61389.1 |
| GO:0050136 | NADH dehydrogenase (quinone) activity | RZR67747.1,RZR67714.1,RZR67091.1,RZR66584.1,RZR65807.1,RZR65658.1,RZR63682.1 |
| GO:0051302 | regulation of cell division | RZR66739.1,RZR65927.1,RZR65855.1,RZR63618.1 |
| GO:0052761 | exo-1,4-beta-D-glucosaminidase activity | RZR65709.1,RZR63081.1 |
| GO:0055070 | copper ion homeostasis | RZR65869.1,RZR64616.1,RZR61677.1 |
| GO:0060303 | regulation of nucleosome density | RZR60833.1,RZR60062.1 |
| GO:0070814 | hydrogen sulfide biosynthetic process | RZR59424.1,RZR65819.1,RZR62803.1 |
| GO:0071577 | zinc ion transmembrane transport | RZR70090.1,RZR63877.1 |
| GO:0071885 | N-terminal protein N-methyltransferase activity | RZR60150.1,RZR60149.1 |
| GO:0090529 | cell septum assembly | RZR59323.1,RZR66739.1,RZR65927.1,RZR65855.1,RZR63618.1 |
| GO:0097428 | protein maturation by iron-sulfur cluster transfer | RZR69943.1,RZR69929.1,RZR68771.1,RZR67568.1,RZR62442.1,RZR61021.1 |
| GO:0097599 | xylanase activity | RZR67560.1,RZR64345.1 |
| GO:0099111 | microtubule-based transport | RZR66739.1,RZR65128.1,RZR65030.1 |
| GO:0101030 | tRNA-guanine transglycosylation | RZR62920.1,RZR61672.1 |
| GO:0106074 | aminoacyl-tRNA metabolism involved in translational fidelity | RZR69911.1,RZR67956.1,RZR64344.1,RZR63849.1,RZR61589.1,RZR61190.1,RZR60480.1 |
| GO:1901661 | quinone metabolic process | RZR70285.1,RZR69906.1,RZR68209.1,RZR67748.1,RZR67355.1,RZR66583.1,RZR66085.1,RZR64164.1,RZR64045.1,RZR60204.1 |
| GO:1901663 | quinone biosynthetic process | RZR70285.1,RZR69906.1,RZR68209.1,RZR67748.1,RZR67355.1,RZR66583.1,RZR66085.1,RZR64164.1,RZR64045.1,RZR60204.1 |
| GO:1901891 | regulation of cell septum assembly | RZR66739.1,RZR65927.1,RZR65855.1,RZR63618.1 |
