# Supplementary material for: Chitosan modulates Pochonia chlamydosporia gene expression during nematode egg parasitism
Source: Environ Microbiol. 2021 Feb 5;23(9):4980–97. doi: 10.1111/1462-2920.15408 (PMC8518118; doi:10.1111/1462-2920.15408)
Supplement: Supplementary file 9 — Supplementary Table 3. Classification and statistics of the 180 genes represented in clusters in Fig. 4. [file EMI-23-4980-s008.docx]

**Supplementary Table 3.** Classification and statistics of the 180 genes represented in clusters in Figure 4.

|  |  |  | **MODEL** | | **p-values VARIABLES** | | | |
| --- | --- | --- | --- | --- | --- | --- | --- | --- |
| **Gene-id** | **Sequence Description** | **Cluster** | **p-value** | **R-squared** | **beta0** | **RKN** | **Chitosan** | **ChitosanRKN** |
| RZR59737.1 | ribosomal RNA adenine methylase transferase | 1 | 2,12E-05 | 0,75 | 9,19E-11 | 0,008698359 | 0,002647175 | 0,03614802 |
| RZR61552.1 | C6 zinc finger domain protein | 1 | 1,43E-06 | 0,72 | 1,89E-09 | NA | 0,000676414 | NA |
| RZR61625.1 | cytochrome P450 ClCP1 | 1 | 1,36E-14 | 0,76 | 0,00039859 | NA | 1,96E-05 | NA |
| RZR64101.1 | oxidoreductase | 1 | 2,69E-07 | 0,77 | 1,82E-12 | 0,0325619 | 0,000908075 | NA |
| RZR65154.1 | Glycerol kinase | 1 | 2,24E-06 | 0,71 | 5,22E-06 | NA | NA | 0,000842956 |
| RZR65940.1 | Ser/Thr protein phosphatase family | 1 | 1,17E-06 | 0,71 | 1,17E-09 | NA | 0,000666811 | NA |
| RZR66553.1 | hypothetical protein I1G 00003503 | 1 | 2,18E-06 | 0,78 | 2,33E-11 | 0,008877594 | 0,003528656 | 0,000662152 |
| RZR66588.1 | AtmA protein | 1 | 9,60E-08 | 0,73 | 3,11E-07 | NA | 0,000360786 | NA |
| RZR66852.1 | Ash2-trithorax family protein | 1 | 7,04E-07 | 0,75 | 1,57E-12 | 0,014075138 | 0,001672165 | NA |
| RZR70254.1 | thioredoxin-like protein | 1 | 2,69E-07 | 0,72 | NA | NA | NA | 0,000566241 |
| RZR70289.1 | extracellular soluble lytic transglycosylase | 1 | 5,88E-09 | 0,78 | 0,009710006 | NA | 0,000319421 | NA |
| RZR59591.1 | tannase and feruloyl esterase | 2 | 8,71E-06 | 0,72 | 1,31E-09 | 0,001291609 | NA | 0,044920193 |
| RZR60329.1 | carboxyl-terminal proteinase | 2 | 9,79E-08 | 0,77 | 0,002208387 | NA | 0,000576046 | 0,019892045 |
| RZR61375.1 | hypothetical protein VFPPC 13287 | 2 | 1,29E-07 | 0,77 | 1,68E-11 | 0,039996345 | 0,0006496 | NA |
| RZR61987.1 | tripeptidyl-peptidase 1 precursor | 2 | 8,03E-07 | 0,73 | 8,45E-07 | NA | 0,000690212 | 0,028783115 |
| RZR62018.1 | metallo-endopeptidase | 2 | 1,99E-05 | 0,70 | 0,000213224 | NA | 0,001825912 | 0,042598187 |
| RZR62208.1 | peptidase A4 family protein | 2 | 7,70E-11 | 0,84 | 0,001934776 | NA | 0,000153052 | 0,013177962 |
| RZR62289.1 | UbiD family decarboxylase | 2 | 2,48E-05 | 0,71 | 1,50E-12 | 0,006524585 | 0,017302563 | NA |
| RZR62805.1 | aspartic protease precursor | 2 | 4,72E-15 | 0,88 | 4,85E-09 | 0,000703134 | 0,000182671 | NA |
| RZR63059.1 | cytochrome P450 alkane hydroxylase | 2 | 6,89E-13 | 0,85 | 1,13E-13 | 0,001573201 | 0,000194942 | NA |
| RZR63158.1 | peptidase A1 | 2 | 2,08E-09 | 0,80 | NA | NA | 0,00086784 | 0,008989406 |
| RZR63191.1 | putative transporter | 2 | 1,12E-06 | 0,76 | 1,86E-11 | NA | 0,000618245 | 0,017844262 |
| RZR63431.1 | Peptidase S8/S53, subtilisin/kexin/sedolisin | 2 | 1,11E-12 | 0,82 | NA | NA | 0,001131574 | 0,00902536 |
| RZR64143.1 | flavin-binding monooxygenase-like family protein | 2 | 3,07E-08 | 0,73 | NA | 0,012903225 | 0,003592655 | NA |
| RZR64245.1 | major facilitator superfamily transporter | 2 | 3,62E-08 | 0,74 | 2,91E-14 | 0,000260514 | NA | NA |
| RZR66026.1 | secreted aspartic proteinase precursor | 2 | 4,72E-07 | 0,76 | NA | NA | 0,001667017 | 0,049454139 |
| RZR66412.1 | thaumatin family protein | 2 | 2,09E-05 | 0,70 | 1,11E-09 | 0,006816374 | 0,013858384 | NA |
| RZR67362.1 | hypothetical protein I1G 00001705 | 2 | 8,52E-06 | 0,72 | 1,73E-12 | 0,000923845 | NA | 0,005065727 |
| RZR68171.1 | Glycoside hydrolase, family 3 | 2 | 4,20E-11 | 0,84 | 4,75E-16 | NA | 7,90E-05 | 0,01203223 |
| RZR68493.1 | Hydrophobin 2 | 2 | 8,13E-13 | 0,82 | 0,019830601 | NA | 0,000178981 | 0,000824031 |
| RZR68929.1 | indoleamine 2,3-dioxygenase pyrrole 2,3-dioxygenase | 2 | 8,33E-10 | 0,83 | 1,92E-10 | 0,000445206 | 0,007187223 | NA |
| RZR69491.1 | metalloprotease | 2 | 1,15E-06 | 0,70 | 3,06E-10 | 0,00351997 | 0,008562668 | NA |
| RZR69653.1 | ER lumen protein retaining receptor | 2 | 2,60E-14 | 0,87 | 1,08E-16 | 0,000885557 | NA | 0,046226875 |
| RZR69884.1 | hemolysin-III related protein | 2 | 6,92E-10 | 0,82 | 7,87E-14 | 0,012427164 | 0,000294281 | NA |
| RZR61845.1 | glycoside hydrolase family 75 protein | 3 | 1,81E-16 | 0,82 | NA | 0,01440303 | 0,002688108 | NA |
| RZR61846.1 | Major facilitator superfamily domain, general substrate transporter | 3 | 3,92E-25 | 0,90 | NA | NA | 0,000215731 | 0,021332233 |
| RZR61856.1 | glucokinase | 3 | 1,26E-17 | 0,86 | NA | 0,012555689 | 0,000994849 | NA |
| RZR62940.1 | glycoside hydrolase family 75 | 3 | 6,29E-15 | 0,84 | NA | NA | 0,004989657 | 0,007709865 |
| RZR63081.1 | glycoside hydrolase family 2 protein | 3 | 1,30E-15 | 0,83 | NA | NA | 0,002858366 | 0,009563879 |
| RZR63795.1 | glycoside hydrolase family 75 | 3 | 1,92E-18 | 0,84 | NA | NA | 0,001040588 | 0,023555752 |
| RZR64799.1 | floculation protein FLO1 | 3 | 2,47E-24 | 0,91 | 0,038841391 | 5,51E-06 | NA | NA |
| RZR64948.1 | fungal chitosanase | 3 | 3,06E-08 | 0,80 | 0,000404351 | NA | 0,000312761 | NA |
| RZR65709.1 | exo-beta-D-glucosaminidase | 3 | 5,47E-10 | 0,76 | 1,41E-08 | NA | 8,85E-05 | NA |
| RZR66181.1 | Glycoside hydrolase, family 3 | 3 | 1,94E-07 | 0,72 | 1,02E-06 | NA | 0,00040643 | NA |
| RZR68026.1 | putative som1 protein | 3 | 8,31E-10 | 0,73 | NA | 0,000716794 | NA | NA |
| RZR68451.1 | maltose permease | 3 | 2,47E-21 | 0,88 | 3,25E-06 | 0,028532475 | 4,10E-06 | NA |
| RZR69242.1 | floculation protein FLO1 | 3 | 9,90E-34 | 0,93 | NA | 1,81E-05 | 0,043295844 | NA |
| RZR70313.1 | chitosanase CSN1 | 3 | 4,47E-07 | 0,78 | 0,000479547 | NA | 0,000305951 | NA |
| RZR59043.1 | hypothetical protein I1G 00005430 | 4 | 8,91E-06 | 0,72 | 7,82E-15 | 0,021240603 | 0,003447325 | NA |
| RZR60396.1 | hypothetical protein I1G 00008473 | 4 | 2,24E-06 | 0,78 | 7,85E-13 | 0,019719857 | 0,001147584 | 0,031355642 |
| RZR60540.1 | activating transcription factor 7a | 4 | 2,10E-10 | 0,85 | 1,08E-11 | 0,00115877 | 0,000497982 | 0,007752554 |
| RZR61231.1 | related to spore coat protein SP96 precursor | 4 | 4,34E-06 | 0,73 | 4,02E-12 | 0,011530648 | 0,004850107 | NA |
| RZR61327.1 | sphingoid long-chain base transporter RSB1 | 4 | 1,19E-06 | 0,79 | 1,62E-09 | 0,008864141 | 0,001749071 | 0,044651039 |
| RZR61534.1 | trehalose synthase (Ccg-9) | 4 | 1,76E-16 | 0,89 | 6,00E-11 | 0,016084366 | 2,73E-05 | NA |
| RZR61827.1 | UPF0075-domain-containing protein | 4 | 4,06E-11 | 0,84 | 6,85E-13 | 0,000359423 | 0,003369614 | NA |
| RZR61872.1 | 2OG-Fe(II) oxygenase | 4 | 1,92E-18 | 0,91 | 1,77E-11 | 0,001543711 | 3,00E-05 | 0,002348046 |
| RZR62083.1 | beta-lactamase domain-containing protein | 4 | 7,18E-10 | 0,82 | 5,62E-10 | 0,004803632 | 0,000570214 | NA |
| RZR62528.1 | xylulokinase | 4 | 2,63E-06 | 0,73 | 3,07E-11 | 0,006333039 | 0,007739442 | NA |
| RZR62553.1 | Ppx/GppA phosphatase | 4 | 1,68E-07 | 0,81 | 7,26E-08 | 0,022330642 | 0,000936063 | 0,028158984 |
| RZR63790.1 | cyclin-like F-box | 4 | 1,32E-05 | 0,71 | 5,30E-14 | 0,019656586 | 0,0043378 | NA |
| RZR63863.1 | mitochondrial inheritance component MDM10 | 4 | 1,30E-09 | 0,85 | 1,39E-13 | 0,003525063 | 0,000241835 | 0,00394975 |
| RZR64014.1 | Amidase family protein | 4 | 4,87E-06 | 0,72 | 9,47E-12 | 0,011395445 | 0,004587036 | NA |
| RZR64754.1 | alcohol dehydrogenase superfamily, zinc-containing | 4 | 1,16E-08 | 0,80 | 1,13E-14 | 0,008594593 | 0,000720393 | NA |
| RZR65419.1 | DUF4419 domain protein | 4 | 4,18E-25 | 0,94 | 1,72E-09 | 0,002504289 | 2,47E-05 | 0,021626505 |
| RZR65430.1 | LEA domain protein | 4 | 1,58E-05 | 0,71 | 5,28E-11 | 0,015557264 | 0,008531131 | NA |
| RZR65521.1 | alcohol dehydrogenase | 4 | 3,13E-06 | 0,76 | 1,15E-09 | 0,005604757 | 0,003447143 | 0,038507816 |
| RZR65936.1 | CND01770-like protein | 4 | 2,07E-07 | 0,78 | 1,31E-08 | 0,024241445 | 0,000662837 | 0,018418582 |
| RZR66600.1 | uncharacterized protein I1G 00003465 | 4 | 2,21E-14 | 0,89 | 1,14E-07 | 0,000665242 | 0,000291518 | 0,003320057 |
| RZR66738.1 | glutamate decarboxylase | 4 | 7,25E-81 | 0,98 | 8,93E-14 | 7,38E-07 | 1,75E-06 | 0,000329095 |
| RZR67311.1 | phosphoglycerate mutase family protein | 4 | 2,62E-08 | 0,77 | 2,48E-06 | 0,02900596 | 0,001160843 | NA |
| RZR68363.1 | glutamyl-tRNA(Gln) amidotransferase subunit A | 4 | 3,67E-06 | 0,73 | 1,63E-14 | 0,0165851 | 0,002913831 | NA |
| RZR69039.1 | DUF1929 multi-domain protein | 4 | 2,72E-13 | 0,87 | 1,10E-07 | 0,0040288 | 0,000341637 | 0,010930265 |
| RZR69040.1 | double-stranded RNA binding motif domain-containing protein | 4 | 1,30E-11 | 0,83 | 3,40E-08 | 0,004836196 | 0,000209456 | 0,00544994 |
| RZR69492.1 | NADP-dependent leukotriene B4 12-hydroxydehydrogenase | 4 | 1,45E-08 | 0,83 | 4,17E-08 | 0,001595791 | 0,001191395 | 0,013467974 |
| RZR69559.1 | FAD dependent oxidoreductase superfamily protein | 4 | 9,48E-05 | 0,72 | 3,72E-10 | 0,009850179 | 0,003462301 | 0,018087979 |
| RZR69669.1 | mercuric reductase | 4 | 1,19E-09 | 0,82 | 1,39E-13 | 0,001397439 | 0,00196511 | NA |
| RZR59077.1 | hypothetical protein I1G 00005424 | 5 | 3,28E-10 | 0,85 | 2,27E-09 | 0,002244844 | 0,000378872 | 0,009535601 |
| RZR59322.1 | mitochondrial integral membrane protein | 5 | 1,15E-09 | 0,86 | 6,32E-10 | 0,004896231 | 0,000493169 | 0,018917485 |
| RZR60003.1 | cell wall glycoprotein | 5 | 8,42E-08 | 0,79 | 1,80E-11 | 0,002057138 | 0,005764511 | NA |
| RZR60844.1 | glycerol-3-phosphate dehydrogenase | 5 | 3,70E-07 | 0,80 | 5,08E-10 | 0,004697231 | 0,001213655 | 0,016531009 |
| RZR61002.1 | FAD dependent oxidoreductase | 5 | 6,29E-06 | 0,73 | 4,29E-08 | 0,041319933 | 0,001246404 | 0,02576741 |
| RZR61102.1 | heat shock protein 30 | 5 | 4,04E-09 | 0,84 | 5,58E-12 | 0,01537689 | 0,000463346 | 0,03453692 |
| RZR61435.1 | repetitive proline-rich cell wall protein | 5 | 2,86E-08 | 0,80 | 6,66E-08 | 0,006270455 | 0,002138404 | NA |
| RZR62414.1 | hypothetical protein I1G 00009714 | 5 | 6,32E-14 | 0,89 | 9,19E-09 | 0,000400537 | 0,000369653 | 0,004796235 |
| RZR62422.1 | related to short-chain alcohol dehydrogenase | 5 | 1,67E-09 | 0,83 | 5,35E-08 | 0,010667213 | 0,000490136 | 0,019383474 |
| RZR62495.1 | related to pathway-specific regulatory protein nit-4 | 5 | 2,02E-06 | 0,79 | 3,25E-11 | 0,006107343 | 0,002833256 | 0,046631061 |
| RZR63007.1 | hypothetical protein I1G 00005595 | 5 | 6,65E-05 | 0,74 | 1,58E-08 | 0,007716095 | 0,007346364 | 0,036972216 |
| RZR63798.1 | hypothetical protein I1G 00010076 | 5 | 1,16E-05 | 0,72 | 6,62E-12 | 0,024585702 | 0,003599344 | NA |
| RZR63930.1 | Nitrogen assimilation transcription factor nirA | 5 | 1,15E-06 | 0,76 | 2,61E-08 | 0,047324353 | 0,000909734 | 0,029841387 |
| RZR64134.1 | surfeit 1 | 5 | 1,38E-10 | 0,86 | 3,17E-13 | 0,0005831 | 0,001037567 | 0,01449137 |
| RZR64310.1 | hypothetical protein I1G 00000070 | 5 | 7,85E-08 | 0,75 | 2,59E-07 | 0,01880738 | 0,000979996 | 0,020389611 |
| RZR64989.1 | related to mfs-multidrug-resistance transporter | 5 | 3,24E-11 | 0,85 | 3,02E-08 | 0,002979335 | 0,00034621 | 0,00798439 |
| RZR65028.1 | farnesyl pyrophosphate synthetase 1 | 5 | 1,14E-05 | 0,73 | 9,28E-08 | 0,016317561 | 0,002835535 | 0,041157397 |
| RZR65196.1 | transcription factor Cys6 | 5 | 5,36E-06 | 0,77 | 1,63E-11 | 0,020472355 | 0,00141977 | 0,034282715 |
| RZR65938.1 | HHE domain containing protein | 5 | 3,81E-06 | 0,74 | 3,34E-07 | 0,018149104 | 0,000538493 | 0,00211137 |
| RZR66003.1 | potassium channel | 5 | 2,62E-16 | 0,82 | 6,78E-07 | 0,001539915 | 0,000334061 | 0,013853958 |
| RZR66182.1 | major facilitator superfamily transporter | 5 | 9,02E-05 | 0,74 | 1,56E-07 | 0,01781534 | 0,002687858 | 0,016711991 |
| RZR67249.1 | hypothetical protein I1G 00007967 | 5 | 9,59E-07 | 0,79 | 3,12E-08 | 0,012092074 | 0,001655056 | 0,033786592 |
| RZR67605.1 | alpha beta hydrolase fold-1 protein | 5 | 2,45E-11 | 0,86 | 7,78E-08 | 0,007465191 | 0,00040229 | 0,049071913 |
| RZR68130.1 | DNA-binding WRKY domain-containing protein | 5 | 0,00051638 | 0,70 | 1,28E-07 | 0,016904711 | 0,005863166 | 0,015640265 |
| RZR69481.1 | hypothetical protein I1G 00002298 | 5 | 0,00022206 | 0,71 | 6,17E-10 | 0,026996982 | 0,002823382 | 0,015084795 |
| RZR69976.1 | phosphotransferase enzyme family protein | 5 | 8,51E-07 | 0,78 | 2,04E-10 | 0,006501031 | 0,001070404 | 0,01133496 |
| RZR70243.1 | MUS38-like protein | 5 | 4,62E-19 | 0,90 | 2,25E-07 | 0,000340414 | 0,000148234 | 0,001995497 |
| RZR59365.1 | SUR7 protein | 6 | 2,04E-07 | 0,70 | 1,05E-08 | 0,014492484 | 0,002642931 | NA |
| RZR59815.1 | C6 transcription factor | 6 | 0,00058418 | 0,70 | 1,11E-07 | 0,023428286 | 0,005564733 | 0,004718679 |
| RZR60184.1 | clr5 domain-containing protein | 6 | 7,71E-06 | 0,71 | 7,28E-09 | NA | 0,00140622 | NA |
| RZR60358.1 | PDZ-binding protein, CRIPT | 6 | 3,22E-05 | 0,75 | 3,59E-08 | 0,005578125 | 0,005748265 | 0,024854601 |
| RZR61033.1 | glucose repressible protein Grg1 | 6 | 3,90E-17 | 0,90 | 3,04E-10 | 0,000580236 | 7,06E-05 | 0,002091176 |
| RZR61034.1 | thioredoxin domain-containing protein | 6 | 1,54E-11 | 0,84 | 2,75E-08 | 0,001738377 | 0,000314649 | 0,004457657 |
| RZR61602.1 | glucose repressible protein Grg1 | 6 | 0,00019573 | 0,70 | 1,20E-08 | 0,047560863 | 0,003335153 | 0,042443624 |
| RZR61787.1 | hypothetical protein I1G 00007802 | 6 | 0,00018511 | 0,70 | 1,39E-08 | 0,006143371 | 0,006669899 | 0,01545835 |
| RZR62047.1 | cell surface flocculin, putative | 6 | 9,74E-07 | 0,76 | 3,13E-07 | 0,018165253 | 0,001186945 | 0,020835052 |
| RZR62271.1 | FAD dependent oxidoreductase | 6 | 0,0001126 | 0,71 | 6,67E-08 | 0,036725469 | 0,002262657 | 0,008241529 |
| RZR62287.1 | membrane-associating domain-containing protein | 6 | 5,94E-05 | 0,74 | 5,62E-10 | 0,008030018 | 0,003093568 | 0,011206789 |
| RZR62487.1 | PQ loop repeat protein | 6 | 9,50E-05 | 0,73 | 9,89E-10 | 0,018972491 | 0,00296979 | 0,023619344 |
| RZR62546.1 | tetratricopeptide repeat domain protein | 6 | 3,85E-06 | 0,72 | 1,63E-09 | 0,049539711 | 0,000999596 | 0,030208776 |
| RZR62583.1 | gluconate 5-dehydrogenase | 6 | 0,00032324 | 0,71 | 3,67E-07 | 0,025222439 | 0,003998302 | 0,006929759 |
| RZR62837.1 | short chain dehydrogenase | 6 | 7,12E-07 | 0,80 | 3,12E-08 | 0,011438849 | 0,000988194 | 0,010695506 |
| RZR63193.1 | ATPase type 13A2 | 6 | 5,29E-05 | 0,74 | 4,17E-10 | 0,017484262 | 0,003584565 | 0,0492203 |
| RZR63453.1 | protein bli-3 | 6 | 2,74E-10 | 0,83 | 3,06E-07 | 0,010673626 | 0,000652369 | 0,018667617 |
| RZR64929.1 | FMN-dependent alpha-hydroxy acid dehydrogenase | 6 | 1,14E-07 | 0,81 | 1,08E-06 | 0,01317432 | 0,001957678 | 0,030921237 |
| RZR64973.1 | ---NA--- | 6 | 1,54E-06 | 0,77 | 1,53E-06 | 0,04011468 | 0,002835951 | 0,039168198 |
| RZR65012.1 | mannose-6-phosphate isomerase | 6 | 4,21E-08 | 0,79 | 1,37E-08 | 0,011303937 | 0,000761702 | 0,023968829 |
| RZR65027.1 | short-chain dehydrogenase | 6 | 1,45E-07 | 0,78 | 1,36E-08 | 0,043237812 | 0,000636453 | 0,033714864 |
| RZR65332.1 | hypothetical protein I1G 00001300 | 6 | 6,26E-13 | 0,86 | 4,49E-09 | 0,002341786 | 0,000136664 | 0,003819107 |
| RZR65455.1 | hypothetical protein I1G 00002240 | 6 | 2,12E-07 | 0,81 | 1,10E-08 | 0,006952244 | 0,001109049 | 0,01618378 |
| RZR65839.1 | diacylglycerol o-acyltransferase | 6 | 2,45E-06 | 0,78 | 5,03E-12 | 0,0054237 | 0,001987836 | 0,023205429 |
| RZR65976.1 | DEAD-2 domain-containing protein | 6 | 1,13E-06 | 0,77 | 1,11E-08 | 0,0216627 | 0,000995168 | 0,02157004 |
| RZR66043.1 | pyruvate decarboxylase | 6 | 1,75E-08 | 0,82 | 7,09E-11 | 0,008838851 | 0,000524316 | 0,017245018 |
| RZR66454.1 | hypothetical protein I1G 00004264 | 6 | 3,84E-07 | 0,78 | 1,32E-07 | 0,043189078 | 0,000886837 | 0,038001734 |
| RZR66613.1 | oxidoreductase | 6 | 2,07E-07 | 0,78 | 4,00E-08 | 0,030046384 | 0,000719725 | 0,027452599 |
| RZR66872.1 | Acid phosphatase | 6 | 1,41E-05 | 0,73 | 2,10E-09 | 0,017330013 | 0,002238087 | 0,030417433 |
| RZR67229.1 | 3-dehydroshikimate dehydratase protein | 6 | 1,47E-08 | 0,80 | 1,41E-07 | 0,023133983 | 0,000662042 | 0,026501044 |
| RZR67230.1 | NADP-dependent alcohol dehydrogenase C | 6 | 2,97E-08 | 0,81 | 8,21E-08 | 0,009105295 | 0,00075629 | 0,014139609 |
| RZR67257.1 | putative phosphatidylinositol phosphate kinase | 6 | 1,12E-11 | 0,86 | 1,65E-07 | 0,007494304 | 0,000457264 | 0,013224033 |
| RZR67654.1 | hypothetical protein I1G 00000537 | 6 | 2,52E-05 | 0,75 | 9,15E-09 | 0,006544466 | 0,002278417 | 0,004780362 |
| RZR68164.1 | MARVEL-like domain protein | 6 | 3,15E-07 | 0,80 | 6,58E-08 | 0,015612069 | 0,001090854 | 0,034007718 |
| RZR69146.1 | VOC family protein | 6 | 3,91E-07 | 0,79 | 6,44E-09 | 0,006072567 | 0,000884438 | 0,007013868 |
| RZR69535.1 | NAD(P)-binding domain protein | 6 | 1,92E-09 | 0,82 | 2,29E-07 | 0,018392411 | 0,000694933 | 0,022515751 |
| RZR70187.1 | lysophospholipase Plb1 | 6 | 0,00045843 | 0,71 | 3,24E-06 | 0,048020132 | 0,00624425 | 0,039966362 |
| RZR70238.1 | predicted protein | 6 | 2,12E-12 | 0,86 | 1,15E-09 | 0,00736826 | 0,000110942 | 0,006600753 |
| RZR60847.1 | DDHD domain protein | 7 | 1,39E-07 | 0,74 | 1,29E-17 | NA | 0,000363716 | NA |
| RZR61362.1 | thioesterase family protein | 7 | 1,18E-06 | 0,70 | 8,43E-15 | NA | 0,00066287 | NA |
| RZR62622.1 | MFS multidrug transporter | 7 | 3,32E-06 | 0,71 | 4,34E-09 | NA | 0,001026608 | NA |
| RZR63094.1 | maltose permease | 7 | 1,22E-08 | 0,70 | 8,67E-08 | NA | 0,000274674 | NA |
| RZR63110.1 | Major facilitator superfamily domain, general substrate transporter | 7 | 3,45E-07 | 0,71 | 9,08E-15 | NA | 0,000459648 | NA |
| RZR64573.1 | serine/threonine-protein kinase Sgk2 | 7 | 7,12E-08 | 0,74 | 1,34E-17 | NA | 0,000305999 | NA |
| RZR65638.1 | alpha/beta fold hydrolase | 7 | 6,30E-08 | 0,74 | 3,03E-12 | NA | 0,000304124 | NA |
| RZR66350.1 | twin-arginine translocation pathway signal | 7 | 6,83E-12 | 0,85 | 1,36E-09 | 0,01124647 | 0,000139448 | NA |
| RZR66560.1 | DEAD-2 domain-containing protein | 7 | 4,54E-06 | 0,72 | 4,73E-10 | NA | 0,000692769 | 0,048931884 |
| RZR67506.1 | ycfA-like protein domain-containing protein | 7 | 3,87E-07 | 0,71 | 4,74E-10 | NA | 0,000514538 | NA |
| RZR59706.1 | Major facilitator superfamily domain, general substrate transporter | 8 | 7,12E-11 | 0,83 | 1,08E-09 | 0,000118405 | NA | 0,001775982 |
| RZR59707.1 | salicylate hydroxylase | 8 | 5,83E-15 | 0,90 | 3,27E-11 | 4,79E-05 | 0,045600642 | 0,001342184 |
| RZR68233.1 | het domain-containing protein | 8 | 7,71E-08 | 0,74 | 3,98E-12 | 0,000317945 | NA | NA |
| RZR68254.1 | rRNA maturation protein | 8 | 6,83E-07 | 0,76 | 2,25E-13 | 0,001019943 | NA | 0,001472681 |
| RZR59485.1 | Longevity assurance, LAG1/LAC1 | 9 | 8,16E-06 | 0,71 | 1,05E-13 | NA | 0,001264818 | 0,007487858 |
| RZR59733.1 | heme peroxidase | 9 | 1,90E-10 | 0,86 | 6,11E-10 | 0,004232821 | 0,000137623 | 0,008152997 |
| RZR59927.1 | dynamin family domain-containing protein | 9 | 2,16E-17 | 0,89 | 3,55E-12 | NA | 6,46E-06 | NA |
| RZR60129.1 | ankyrin repeat domain-containing protein 52 | 9 | 2,68E-10 | 0,85 | 1,43E-10 | 0,003401443 | 0,000208315 | 0,008448365 |
| RZR60694.1 | glycoside hydrolase family 47 protein | 9 | 4,01E-09 | 0,81 | 9,71E-12 | NA | 0,0001628 | 0,014471496 |
| RZR60922.1 | phospho-2-dehydro-3-deoxyheptonate aldolase | 9 | 7,53E-08 | 0,77 | 3,22E-13 | NA | 0,000606673 | 0,00117797 |
| RZR61136.1 | two-component osmosensing histidine kinase (Bos1) | 9 | 2,24E-05 | 0,73 | 4,86E-09 | 0,003004114 | 0,001941621 | 0,002129485 |
| RZR61142.1 | Major facilitator superfamily domain, general substrate transporter | 9 | 3,81E-06 | 0,72 | 3,90E-05 | NA | 0,002136576 | 0,002496491 |
| RZR61414.1 | Zinc finger, C2H2-type/integrase, DNA-binding protein | 9 | 6,39E-05 | 0,73 | 9,10E-09 | 0,004847125 | 0,002417458 | 0,002663699 |
| RZR61829.1 | catalytic protein | 9 | 2,09E-07 | 0,72 | 1,89E-07 | NA | 0,000454093 | NA |
| RZR63227.1 | alcohol dehydrogenase | 9 | 2,50E-09 | 0,77 | 3,84E-14 | NA | 0,000132553 | NA |
| RZR64079.1 | 3-beta hydroxysteroid dehydrogenase/isomerase | 9 | 2,24E-32 | 0,95 | 3,75E-09 | 0,002294886 | 3,14E-06 | 0,00058865 |
| RZR64444.1 | GMC oxidoreductase | 9 | 1,00E-14 | 0,89 | 3,60E-08 | 0,017822822 | 5,38E-05 | 0,000593688 |
| RZR64639.1 | hypothetical protein I1G 00010772 | 9 | 1,94E-07 | 0,79 | 0,000286709 | 0,027473957 | 0,000615657 | 0,01393205 |
| RZR65223.1 | WSC domain-containing protein | 9 | 4,02E-08 | 0,76 | 0,000716949 | NA | 0,00023488 | NA |
| RZR66542.1 | RTA-like protein | 9 | 1,15E-05 | 0,71 | 1,60E-12 | NA | 0,001110323 | 0,030723066 |
| RZR66821.1 | pathway-specific nitrogen regulator | 9 | 0,00016921 | 0,71 | 7,55E-09 | 0,011026213 | 0,002469118 | 0,00572117 |
| RZR67695.1 | elastinolytic metalloproteinase Mep | 9 | 2,43E-07 | 0,78 | 5,48E-11 | NA | 0,000673354 | 0,005547493 |
| RZR68291.1 | cystathionine gamma-synthase | 9 | 9,01E-05 | 0,73 | 4,51E-10 | 0,043084834 | 0,00171668 | 0,016503009 |
| RZR68721.1 | kinesin | 9 | 6,25E-07 | 0,79 | 1,25E-09 | 0,005946067 | 0,000585887 | 0,008241086 |
| RZR68738.1 | methyltransferase, putative | 9 | 1,31E-17 | 0,91 | 9,68E-11 | 0,00131373 | 1,81E-05 | 0,000243314 |
| RZR68751.1 | Carbohydrate-binding WSC | 9 | 2,02E-06 | 0,76 | 2,08E-09 | 0,003922552 | 0,000560885 | 0,004403837 |
| RZR69156.1 | hypothetical protein I1G 00011621 | 9 | 1,18E-05 | 0,77 | 2,65E-07 | 0,002948306 | 0,003027961 | 0,001333901 |
| RZR69865.1 | cytochrome P450 oxidoreductase | 9 | 3,99E-07 | 0,77 | 2,53E-05 | 0,017992344 | 0,000464635 | 0,007093501 |
| RZR69874.1 | related to tenascin X precursor | 9 | 3,32E-08 | 0,71 | 9,30E-11 | NA | 0,000211997 | NA |
