# Supplementary material for: Chitosan modulates Pochonia chlamydosporia gene expression during nematode egg parasitism
Source: Environ Microbiol. 2021 Feb 5;23(9):4980–97. doi: 10.1111/1462-2920.15408 (PMC8518118; doi:10.1111/1462-2920.15408)
Supplement: Supplementary file 10 — Supplementary Table 4. Quality of RNA extracted. All samples were sent to Macrogen to perform RNA‐seq analyses. [file EMI-23-4980-s002.docx]

**Supplementary Table 4.** Quality of RNA extracted. All samples were sent to Macrogen Inc. to perform RNAseq analyses.

| **Sample name** | **Concentration (ng/ul)** | **RIN** | **rRNA ratio** |
| --- | --- | --- | --- |
| Pc_1 | 298.629 | 9.7 | 1.7 |
| PcQ_1 | 138.043 | 9.2 | 1.7 |
| PcRKN_1 | 106.785 | 9.2 | 1.4 |
| PcRKNQ_1 | 65.785 | 9 | 1.4 |
| Pc_2 | 194.360 | 8.4 | 1.1 |
| PcQ_2 | 23.556 | 9 | 1.3 |
| PcRKN_2 | 82.458 | 9 | 1.5 |
| PcRKNQ_2 | 109.236 | 9.4 | 1.7 |
| Pc_3 | 76.174 | 9.6 | 1.7 |
| PcQ_3 | 89.336 | 9.6 | 1.8 |
| PcRKN_3 | 68.447 | 8.4 | 1.4 |
| PcRKNQ_3 | 106.669 | 8.3 | 1.6 |
