# Supplementary material for: Chitosan modulates Pochonia chlamydosporia gene expression during nematode egg parasitism
Source: Environ Microbiol. 2021 Feb 5;23(9):4980–97. doi: 10.1111/1462-2920.15408 (PMC8518118; doi:10.1111/1462-2920.15408)
Supplement: Supplementary file 11 — Supplementary Table 5. Primers used in all gene expression analyses. [file EMI-23-4980-s005.docx]

**Supplementary Table 5.** Primers used in all gene expression analyses.

| **Name** | **Primer Sequence 5’-3’** | **Protein ID** | **Length** | **Putative Function** |
| --- | --- | --- | --- | --- |
| csn5_F | GCAGCAACGTCTTACAAGG | RZR63795.1 | 149 bp | Glycoside hydrolase family 75 (*csn5*)^1^ |
| csn5_R | ACCGTCACAATCAATGTCCA |  |  |  |
| csn6_F | CTGTCATGGCAGTTGTTTGT | RZR64854.1 | 72 bp | Glycoside hydrolase family 75 (*csn6*)^1^ |
| csn6_R | TGGTTGGCTCTTGATTCGT |  |  |  |
| csn3_F | TCGTTTTGCCTCTTCTTGCC | RZR62940.1 | 235 bp | Glycoside hydrolase family 75 (*csn3*)^1^ |
| csn3_R | TTGACCAGGTTCCCATGTGT |  |  |  |
| csn10_F | CGTTTAACCCACAGGACCAT | RZR70313.1 | 153 bp | Chitosanase CSN10 (*csn10*)^1^ |
| csn10_R | CATTCGGAAAGCACAGCTTC |  |  |  |
| CDA1_F | CGACCTTCTTCGTCATAGGC | RZR66914.1 | 227 bp | Cystathionine beta-lyase (*cda1*)^1^ |
| CDA1_R | AGACCCAGGGCGGTAGTAGT |  |  |  |
| CDA2_F | ACAACCGAGTCAAGGGACAT | RZR69799.1 | 296 bp | Methyltransferase type 11 (*cda2*)^1^ |
| CDA2_R | GTCCAAGTTGGTGGAGATGG |  |  |  |
| Btub_F | TCCCTCGTCTGCACTTCTTCA | RZR65128.1 | 254 bp | ß-tubulin (Housekeeping Gene; HK)^2^ |
| Btub_R | CCATTCGACAAAGTAGGTCGAGTT |  |  |  |
| AllPerm_F | TCGGCATCAACATCATCCTA | RZR69578.1 | 94 bp | Major facilitator superfamily transporter, Allantoate Permease (HK)^3^ |
| AllPerm_R | CCCAGGATGAACCTGACAGT |  |  |  |
| GADPH_F | GCAACACCAACTCCTCCATC | RZR61537.1 | 79 bp | Glyceraldehyde-3-phosphate dehydrogenase (HK)^4^ |
| GADPH_R | TACCAGGAGACCAGCTTGAC |  |  |  |
| Fre2_F | CGGATAGAAAATGGGGGAAT | RZR64658.1 | 92 bp | Ferric-chelate reductase ^5^ |
| Fre2_R | CTGAGTCCGCAGCTCTCTCT |  |  |  |
| MO450_F | GACAGCGAAATACGCCAAAT | RZR62256.1 | 99 bp | Benzoate 4-monooxygenase cytochrome P450 ^5^ |
| MO450_R | GGGAACTTCCTTGGACATGA |  |  |  |
| Halog_F | GTGAGGGCAATGATGTTGTG | RZR68662.1 | 100 pb | Halogenase ^5^ |
| Halog_R | ATGAAGCGCAAGAGTGGTCT |  |  |  |
| MFS61846_F | GGTTCACAATGCGGAGAAAT | RZR61846.1 | 75 bp | Major Facilitator Substrate transpor t^5^ |
| MFS61846_R | GCATCTCCCTTCTTGTCTGC |  |  |  |
| glucok_F | CTGCGCTCATTATCCAGTCA | RZR61856.1 | 75 bp | Glucokinase ^5^ |
| glucok_R | ATTCATTGGCCCTGAACAAG |  |  |  |
| MOFAD_F | TCGCATGACTCTACGACAGG | RZR62842.1 | 96 pb | FAD-dependent monooxygenase ^5^ |
| MOFAD_R | GTGCCATCTTCGTTGGAAGT |  |  |  |
| 66026qF | GACCTCGACAGCAAAAGACC | RZR66026.1 | 179 bp | Secreted aspartic proteinase precursor ^5^ |
| 66026qR | GCTCCAGAAACCTTCACTGC |  |  |  |
| 62042qF | AGTGGGACATCTACGCCATC | RZR62042.1 | 165 bp | Helix-loop-helix DNA-binding protein ^5^ |
| 62042qR | TCACTACCCCATTGCACAGA |  |  |  |
| 63158qF | TCGTGTGAAGCCACAGAGTC | RZR63158.1 | 184 bp | Peptidase A1 ^5^ |
| 63158qR | CCAGTATCCGCGACTACCAT |  |  |  |
| 64159qF | GTAAACCAGCCCTCATTCCA | RZR64159.1 | 173 bp | General substrate transporter ^5^ |
| 64159qR | TGGCATGATTCTCAGACCAG |  |  |  |
| 62046qF | CACATGAAAGTGCCCATCAA | RZR62046.1 | 182 bp | Polyketide synthase ^5^ |
| 62046qR | ATTTCAGGGTGTTGCTCCAG |  |  |  |
| 61625qF | AAGAGTCACCCCTGACGATG | RZR61625.1 | 197 bp | Cytochrome P450 ClCP1 ^5^ |
| 61625qR | CGAGATGGAAGGGCTGTAAA |  |  |  |
| 69240qF | GTTCCACCAAGAGCCACATT | RZR69240.1 | 143 bp | APSES transcription factor ^5^ |
| 69240qR | CGCCTTCTTTTGGCTATGAG |  |  |  |
| 61845qF | GGTATCGAGCCGTTGTCAAT | RZR61845.1 | 106 bp | Glycoside hydrolase family 75 (csn2)^1^ |
| 61845qR | CCTCACCGATAAGTGGAGGA |  |  |  |

^1^ Aranda-Martínez *et al.,* 2016; ^2^ Ward *et al.,* 2012; ^3^ Rosso *et al.,* 2014; ^4^ Escudero *et al.,* 2016; ^5^ this work
